# Supplementary figures and images for: The Plasmodium NOT1-G paralogue is an essential regulator of sexual stage maturation and parasite transmission
Source: PLoS Biol. 2021 Oct 21;19(10):e3001434. doi: 10.1371/journal.pbio.3001434 (PMC8562791; doi:10.1371/journal.pbio.3001434)

Hart *et al.* Figure S1

A.

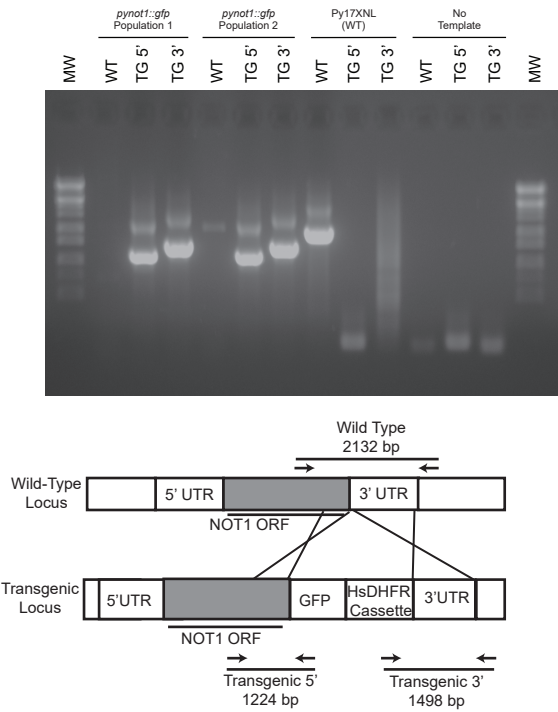

B.

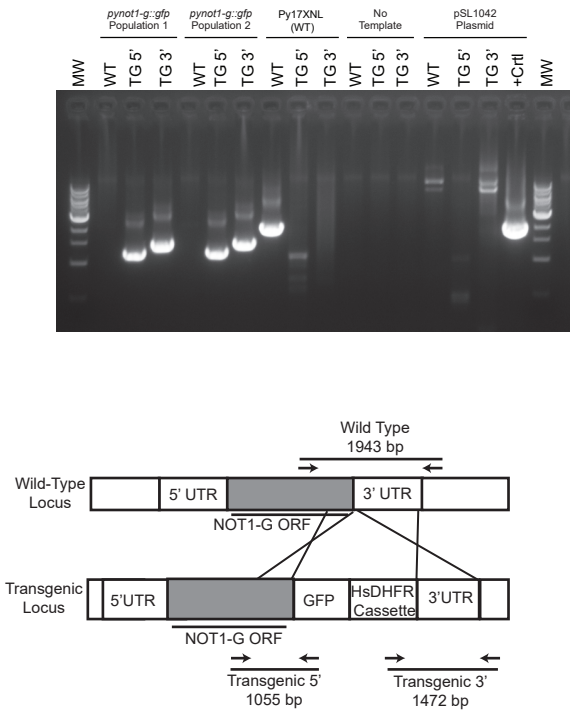

Supplement: S1 Fig — Genomic DNA from transgenic parasites was compared to that of Py17XNL wild-type parasites or no template controls by genotyping PCR. A schematic of the wild-type and designed transgenic loci are provided below each gel image. Primers used are identified in S6 Table for assessing (A) PyNOT1::GFP and (B) PyNOT1-G::GFP parasites. (A) The PSU 1 kb MW ladder [61] or the NEB 1 kb+ MW ladder (B) flanks all experimental lanes. MW, molecular weight. (PDF) [file pbio.3001434.s001.pdf]

Hart *et al.* Figure S2

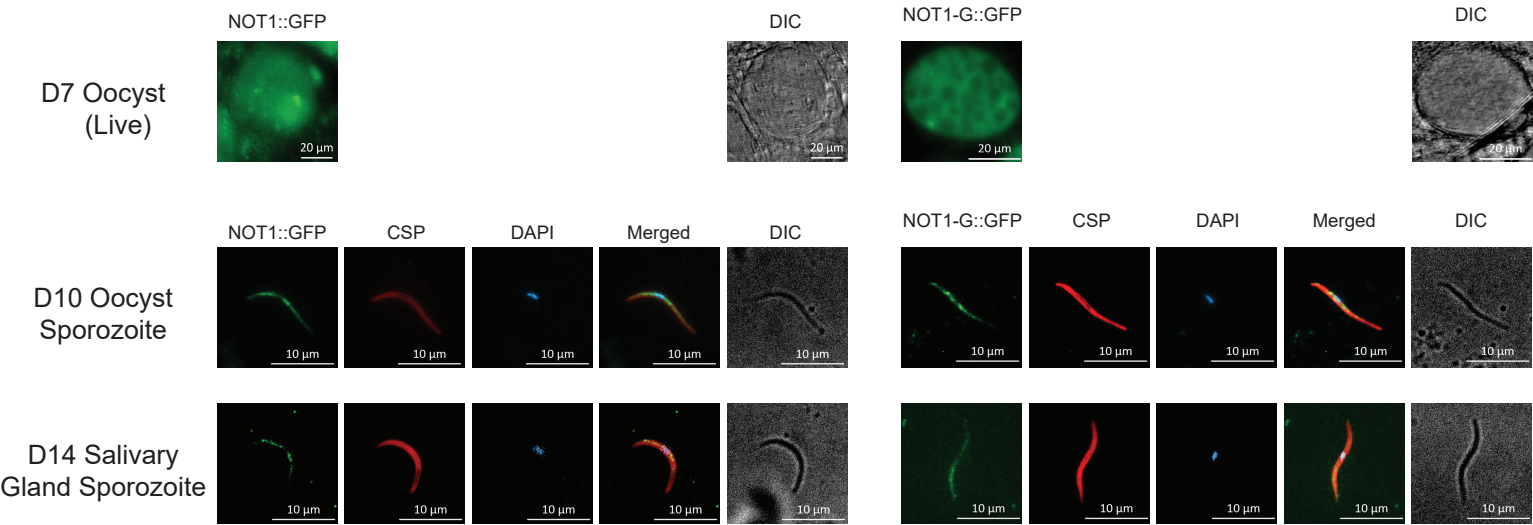

Supplement: S2 Fig — The expression of PyNOT1::GFP and PyNOT1-G::GFP in mosquito stage were assessed by live fluorescence (day 7 oocysts) or by IFA (day 10 oocyst sporozoites, day 14 salivary gland sporozoites). Sporozoites were counterstained with anti-PyCSP and DAPI. Scale bar is 20 μm (oocyst) or 10 μm (sporozoites). DIC, differential interference contrast; IFA, immunofluorescence assay. (PDF) [file pbio.3001434.s002.pdf]

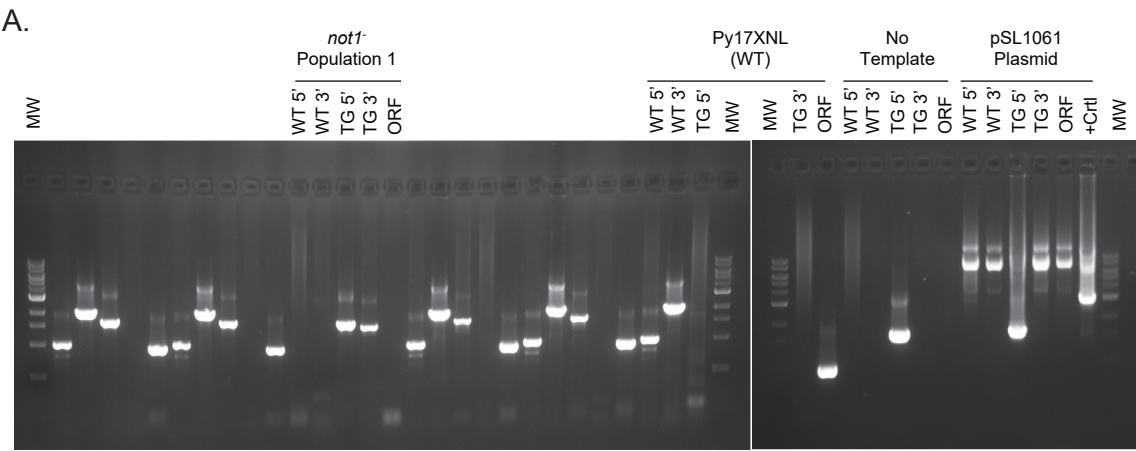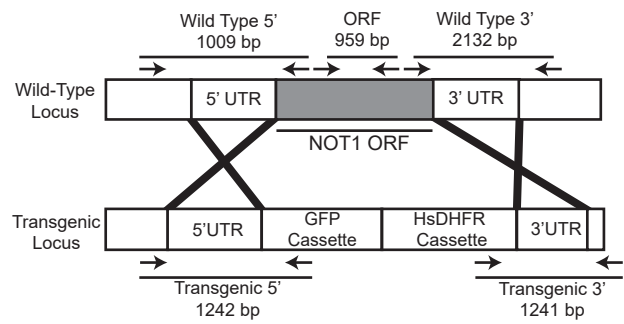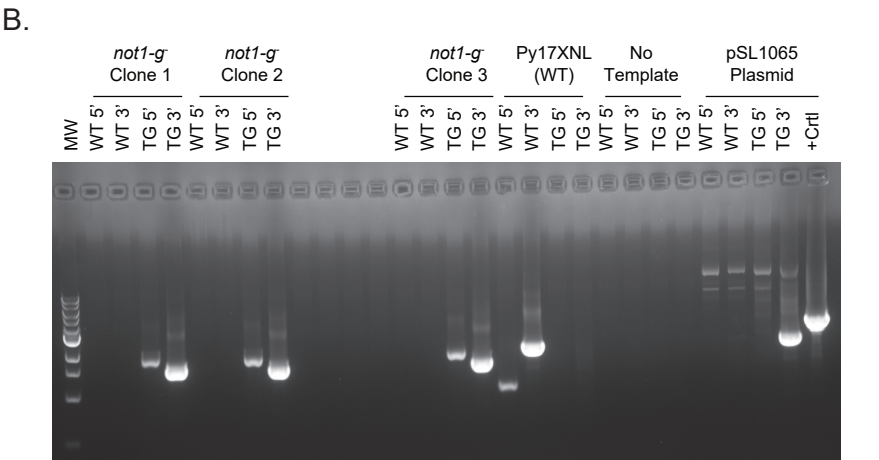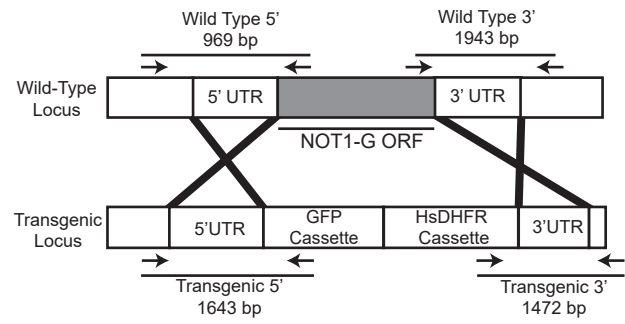

Supplement: S3 Fig — Genomic DNA from (A) pynot1−or (B) pynot1-g− transgenic parasites was compared to that of Py17XNL wild-type parasites or no template controls by genotyping PCR. A schematic of the wild-type and designed transgenic loci are provided below each gel image. Primers used are identified in S6 Table. (A) The NEB 1 kb+ MW ladder (B) flanks all experimental lanes. MW, molecular weight. (PDF) [file pbio.3001434.s003.pdf]

Hart *et al.* Figure S4

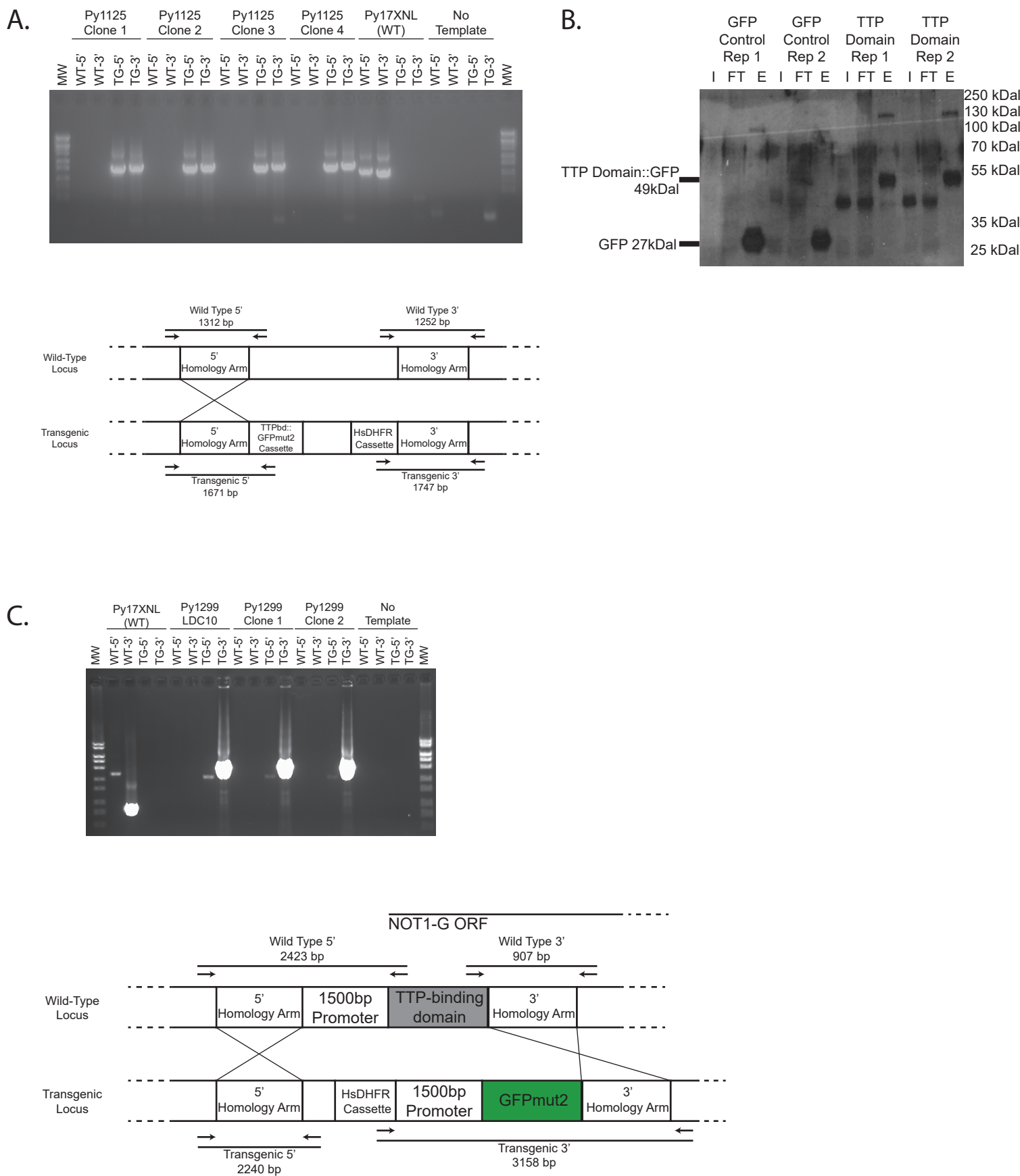

Supplement: S4 Fig — (A, C) Genomic DNA from transgenic parasites was compared to that of Py17XNL wild-type parasites or no template controls by genotyping PCR. A schematic of the wild-type and designed transgenic loci are provided below each gel image. (B) Western blotting of TTPbd::GFP vs Py17XNL WT-GFP parasite lysate enriched by immunoprecipitation with anti-GFP is shown (I = Input, FT = Flow Through, E = Elution). Primers used are identified in S6 Table for assessing (A) PyNOT1-G TTPbd::GFP at the p230p genomic locus and (B) PyNOT1-G::GFP parasites. (A) The PSU 1 kb MW ladder [61] or the NEB 1 kb+ MW ladder (C) flanks all experimental lanes. MW, molecular weight; TTP, tristetraprolin. (PDF) [file pbio.3001434.s004.pdf]

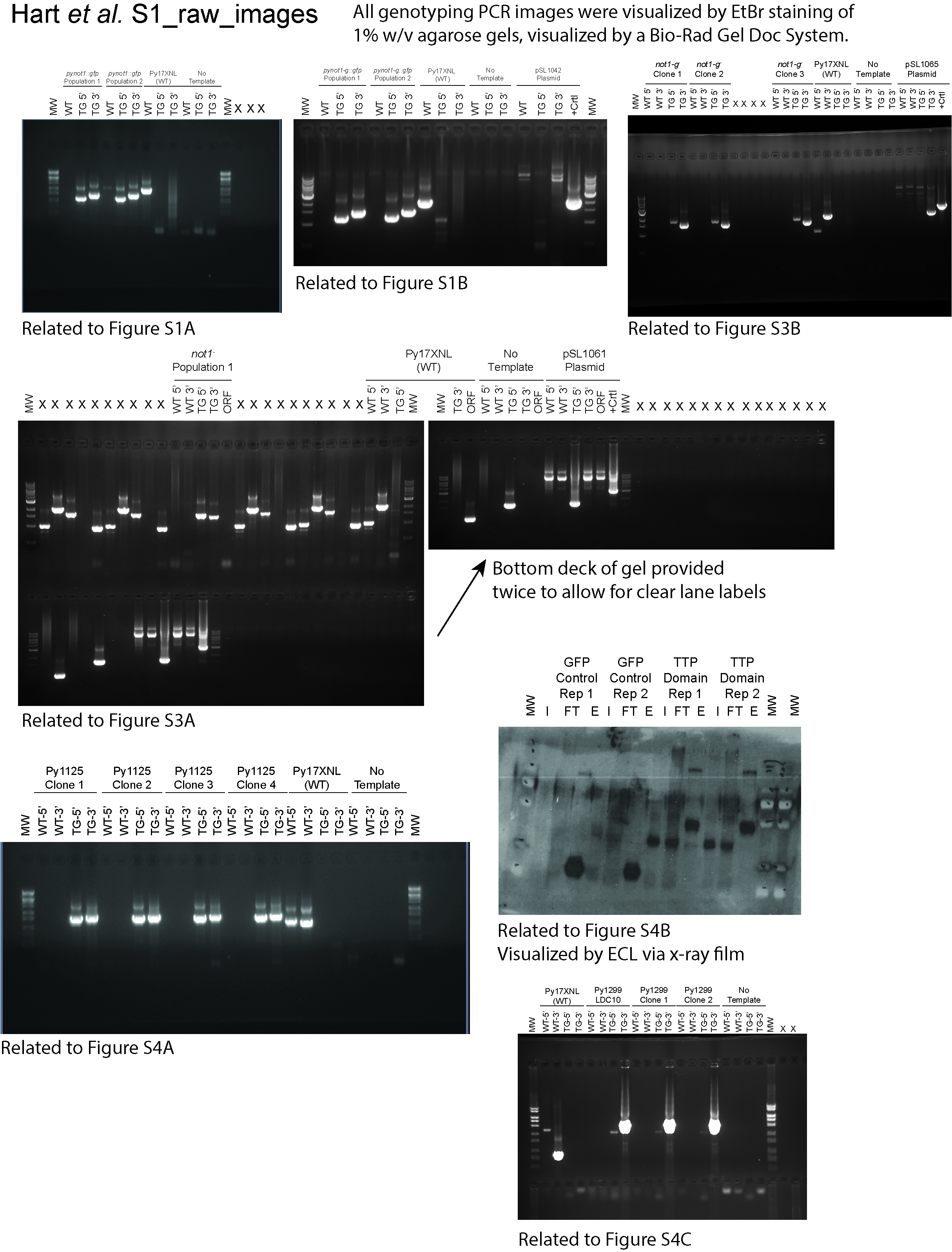

Supplement: S1 Raw Images — (TIF) [file pbio.3001434.s015.tif]
