# Supplementary material for: The Plasmodium NOT1-G paralogue is an essential regulator of sexual stage maturation and parasite transmission
Source: PLoS Biol. 2021 Oct 21;19(10):e3001434. doi: 10.1371/journal.pbio.3001434 (PMC8562791; doi:10.1371/journal.pbio.3001434)
Supplement: S1 File — (DOCX) [file pbio.3001434.s011.docx]

**Hart et al. Supplemental File 1 - Complete Plasmid Sequences**

>Py0489_PyWT-GFPmut2

LOCUS pSL0489__final_ 7758 bp DNA circular 21-APR-2021

SOURCE

ORGANISM

COMMENT Lindner Lab - used to create Py0489 = Py17XNL WT-GFP

COMMENT ApEinfo:methylated:1

FEATURES Location/Qualifiers

exon 6735..7298

/vntifkey="61"

/locus_tag="hDHFR"

/label="hDHFR"

/ApEinfo_label="hDHFR"

/ApEinfo_fwdcolor="pink"

/ApEinfo_revcolor="pink"

/ApEinfo_graphicformat="arrow_data {{0 0.5 0 1 2 0 0 -1 0

-0.5} {} 0} width 5 offset 0"

misc_feature 1898..2471

/locus_tag="PbEF1a promoter"

/label="PbEF1a promoter"

/ApEinfo_label="PbEF1a promoter"

/ApEinfo_fwdcolor="cyan"

/ApEinfo_revcolor="green"

/ApEinfo_graphicformat="arrow_data {{0 0.5 0 1 2 0 0 -1 0

-0.5} {} 0} width 5 offset 0"

misc_feature 2516..3194

/locus_tag="GFPmut2"

/label="GFPmut2"

/ApEinfo_label="GFPmut2"

/ApEinfo_fwdcolor="cyan"

/ApEinfo_revcolor="green"

/ApEinfo_graphicformat="arrow_data {{0 0.5 0 1 2 0 0 -1 0

-0.5} {} 0} width 5 offset 0"

misc_feature 3201..3648

/locus_tag="PbDHFR/TS\3'UTR"

/label="PbDHFR/TS\3'UTR"

/ApEinfo_label="PbDHFR/TS\3'UTR"

/ApEinfo_fwdcolor="#ff0000"

/ApEinfo_revcolor="green"

/ApEinfo_graphicformat="arrow_data {{0 0.5 0 1 2 0 0 -1 0

-0.5} {} 0} width 5 offset 0"

rep_origin 5045..5727

/locus_tag="ColE1 origin"

/label="ColE1 origin"

/ApEinfo_label="ColE1 origin"

/ApEinfo_fwdcolor="gray50"

/ApEinfo_revcolor="gray50"

/ApEinfo_graphicformat="arrow_data {{0 0.5 0 1 2 0 0 -1 0

-0.5} {} 0} width 5 offset 0"

CDS 4288..4947

/locus_tag="AmpR"

/label="AmpR"

/ApEinfo_label="AmpR"

/ApEinfo_fwdcolor="yellow"

/ApEinfo_revcolor="yellow"

/ApEinfo_graphicformat="arrow_data {{0 0.5 0 1 2 0 0 -1 0

-0.5} {} 0} width 5 offset 0"

misc_feature 7305..7755

/locus_tag="PbDHFR-TS 3'UTR"

/label="PbDHFR-TS 3'UTR"

/ApEinfo_label="PbDHFR-TS 3'UTR"

/ApEinfo_fwdcolor="cyan"

/ApEinfo_revcolor="green"

/ApEinfo_graphicformat="arrow_data {{0 0.5 0 1 2 0 0 -1 0

-0.5} {} 0} width 5 offset 0"

misc_feature join(6131..6440,6445..6729)

/locus_tag="PbEF1a 5'UTR"

/label="PbEF1a 5'UTR"

/ApEinfo_label="PbEF1a 5'UTR"

/ApEinfo_fwdcolor="#804040"

/ApEinfo_revcolor="#804040"

/ApEinfo_graphicformat="arrow_data {{0 0.5 0 1 2 0 0 -1 0

-0.5} {} 0} width 5 offset 0"

misc_feature 7..918

/locus_tag="py p230p Homology Arm 1"

/label="py p230p Homology Arm 1"

/ApEinfo_label="py p230p Homology Arm 1"

/ApEinfo_fwdcolor="cyan"

/ApEinfo_revcolor="green"

/ApEinfo_graphicformat="arrow_data {{0 0.5 0 1 2 0 0 -1 0

-0.5} {} 0} width 5 offset 0"

misc_feature 925..1873

/locus_tag="py p230p Homology Arm 2"

/label="py p230p Homology Arm 2"

/ApEinfo_label="py p230p Homology Arm 2"

/ApEinfo_fwdcolor="cyan"

/ApEinfo_revcolor="green"

/ApEinfo_graphicformat="arrow_data {{0 0.5 0 1 2 0 0 -1 0

-0.5} {} 0} width 5 offset 0"

ORIGIN

1 ggtaccAGCT TTGTGTTTTA TTTGGATGTG CAACAATAGA TTTATGATTA ACCAATGGTA

61 AATCATTATA ATCATATATT TCATCTTCAT TTACATATTT TACTAAAACA GTTTTTACAA

121 TTTGTTCTGT TTCTTTATCT ACACAATGAC ATTCAAATTG ATATGATTTT GTTAATGTAT

181 AATTAAAACC AGCTAATGCA TATTCTGGAT TTTCAAATGA ATAGTTAATA ATATTTTTAG

241 TATTAATTTT ATAGGAATCA TGTAGATAGC CTTTTTCATA TACTTGAGTA AAACATTTTT

301 CTGGCATTTT TTTTGTATTT TCGGGGCATC TAAAACCAAA ACTTGTATTT GATTTGGAAA

361 TTATTATACA TAGATCTCCT TCACTATAAG CATTTAAATA ATTATATAAT TCACCTTTTC

421 TAAAATCACA AAATGAAACT GGTCTATCTT TTAAATCTGT TCGAACATGT AATAAAATAA

481 TTGCTCTTCT ATAATCAGTC CCACCATTTG TTACACCACA TTCTATGGTT AGTGGATATG

541 ATCCATGATT TATAAATGAG GTCATAAAAA ATTTAAAACC TTTAAATTGT TCATTTATTT

601 CTGGTATATC TATATCTTTT GTATTAAAAA ATAAATCTGT AAATTTATTT ATAGGAAATA

661 CATTACTTAA ATATTTTATG GTATTAATAG GTTCTTTACC ATATACGATT CTATTATCTG

721 TTATTTGCTC ACAATTATAC ATGAATAAAC TAAATTCATT TATTGTTTTT TCACATATTT

781 GTATTTCACT TAATTTGCTT AAATTATAAT CAAAATTACA CATCTGATAA GGTATATCGT

841 TAAAATATTT AATCGTTTTA CTATCATCAT CAACTATTTT TAAAATGTGA TCAGATGTTG

901 CCATTCCATC ACCAAATGgg gcccTATGGA ACTACATCTA TATAAGAGAT TTTTTTATTT

961 TTATATATTG GTTTTGAATC TAGATATTCA TAATTTTTAA TATATATTGT TCCTATATTT

1021 TTGGTTTTAT AATCTTCACA TGTACAATAA AAATCAAATT CTTTTTTTGT AATTTTTGAA

1081 ATCATTAAAA ATGTTGATGA ATTATTTATA AAATAATCTG CATCTATAAC TTTTATTTCA

1141 TTAAATATAT TTTCGATATT TTCAGTTATT TGTTCATTCA TGTTACTTTT TATATATACC

1201 TTTCTAAAAC AATTTTTTGG ATTAATTTTT CCAGTTGGAC AATTTAAGCT AACTACTATA

1261 TTTTCTTTTA ATTCGATTTC ACAAAATTCA TTTTGTTTTT CTAAATCATA AACATTGGAA

1321 TAGTAAAATT TTTTTTTAGA ATTACCTGAA AAATCACATC CATATAATTT ATTTTTTGTT

1381 GTATTTTGAG AAACCCTTAT CACTGTAATA CCTTTTTGAC CTTTCATATT ATTAATTATT

1441 GTTTTAGTAT TATCACAATA TATTTTCAAT ATTAATACTT TTTCTACAAT TAATGGAGTA

1501 ACAAAAGTGA AATTTCCTTT CTCTTTGAAC ATTTTATTTT TATCTAATAT GATAACACCT

1561 GGTAATATAT CATCTAATTT TTCTTTTTTG TCTAGATACA TTACTTCATC ATTTTCTGGT

1621 ATGTATTCTA TATTTTTATA TTTTTCTAAT TCTTCTGGTT CAACATTTGC ATTTTCTAAA

1681 TAAAAATTAT TTTTTTGACT ACTACGTTGT GATGTTTTCT TATTATTTAT TATGATATTA

1741 CTATCAAAAA TGTCATCATC ACTTTTTACA ATTTTGGGAC ATTTAACTTG AACCATATTC

1801 AGATTCTGTT CTAGATGTAT ATAACATGAA TGAACTGTAT CTACTTCATT AACGGGTTCA

1861 AGAGAATTCG GTAgccgcgg tggcggccgc TctcgagAGC TTAATTCTTT TCGAGCTCTT

1921 TATGCTTAAG TTTACAATTT AATATTCATA CTTTAAGTAT TTTTTGTAGT ATCCTAGATA

1981 TTGTGCTTTA AATGCTCACC CCTCAAAGCA CCAGTAATAT TTTCATCCAC TGAAATACCA

2041 TTAAATTTTC AAAAAAATAC TATGCATATA ATGTTATACA TATAAACATA AAACGCCATG

2101 TAAATCAAAA AATATATAAA AATATGTATA AAAATAAATA TGCACTAAAT ATAAGCTAAT

2161 TATGCATAAA AATTAAAGTG CCCTTTATTA ACTAGctagT CGTAATTATT TATATTTCTA

2221 TGTTATAAAA AAATCCTCAT ATAATAATAT AATTAATATA TGTAATGTTT TTTTTATTTT

2281 ATAATTTTAA TATAAAATAA TATGTAAATT AATTCAAAAA ATAAATATAA TTGTTGTGAA

2341 ACAAAAAACG TAATTTTTTC ATTTGCCTTC AAAATTTAAA TTTATTTTAA TATTTCCTAA

2401 AATATATATA CTTTGTGTAT AAATATATAA AAATATATAT TTGCTTATAA ATAAATAAAA

2461 ATTTTATAAA Aatgactagt agtaaaggag aagaactttt cactggagtt gtcccAATTC

2521 TTGTTGAATT AGATGGTGAT GTTAATGGGC ACAAATTTTC TGTCAGTGGA GAGGGTGAAG

2581 GTGATGCAAC ATACGGAAAA CTTACCCTTA AATTTATTTG CACTACTGGA AAACTACCTG

2641 TTCCATGGCC AACACTTGTC ACTACTTTCG CGTATGGTCT TCAATGCTTT GCGAGATACC

2701 CAGATCATAT GAAACAGCAT GACTTTTTCA AGAGTGCCAT GCCCGAAGGT TATGTACAGG

2761 AAAGAACTAT ATTTTTCAAA GATGACGGGA ACTACAAGAC ACGTGCTGAA GTCAAGTTTG

2821 AAGGTGATAC CCTTGTTAAT AGAATCGAGT TAAAAGGTAT TGATTTTAAA GAAGATGGAA

2881 ACATTCTTGG ACACAAATTG GAATACAACT ATAACTCACA CAATGTATAC ATCATGGCAG

2941 ACAAACAAAA GAATGGAATC AAAGTTAACT TCAAAATTAG ACACAACATT GAAGATGGAA

3001 GCGTTCAACT AGCAGACCAT TATCAACAAA ATACTCCAAT TGGCGATGGC CCTGTCCTTT

3061 TACCAGACAA CCATTACCTG TCCACACAAT CTGCCCTTTC GAAAGATCCC AACGAAAAGA

3121 GAGACCACAT GGTCCTTCTT GAGTTTGTAA CAGCTGCTGG GATTACACAT GGCATGGATG

3181 AACTATACAA ATAAggatcc GTTTTTCTTA CTTATATATT TATACCAATT GATTGTATTT

3241 ATAACTGTAA AAATGTGTAT GTTGTGTGCA TATTTTTTTT TGTGCATGCA CATGCATGTA

3301 AATAGCTAAA ATTATGAACA TTTTATTTTT TGTTCAGAAA AAAAAAACTT TACACACATA

3361 AAATGGCTAG TATGAATAGC CATATTTTAT ATAAATTAAA TCCTATGAAT TTATGACCAT

3421 ATTAAAAATT TAGATATTTA TGGAACATAA TATGTTTGAA ACAATAAGAC AAAATTATTA

3481 TTATTATTAT TATTTTTACT GTTATAATTA TGTTGTCTCT TCAATGATTC ATAAATAGTT

3541 GGACTTGATT TTTAAAATGT TTATAATATG ATTAGCATAG TTAAATAAAA AAAGTTGAAA

3601 AATTAAAAAA AAACATATAA ACACAAATGA TGTTTTTTCC TTCAATTTcg gcgcctgatg

3661 cggtattttc tccttacgca tctgtgcggt atttcacacc gcatatggtg cactctcagt

3721 acaatctgct ctgatgccgc atagttaagc cagccccgac acccgccaac acccgctgac

3781 gcgccctgac gggcttgtct gctcccggca tccgcttaca gacaagctgt gaccgtctcc

3841 gggagctgca tgtgtcagag gttttcaccg tcatcaccga aacgcgcgag acgaaagggc

3901 ctcgtgatac gcctattttt ataggttaat gtcatgataa taatggtttc ttagacgtca

3961 ggtggcactt ttcggggaaa tgtgcgcgga acccctattt gtttattttt ctaaatacat

4021 tcaaatatgt atccgctcat gagacaataa ccctgataaa tgcttcaata atattgaaaa

4081 aggaagagta tgagtattca acatttccgt gtcgccctta ttcccttttt tgcggcattt

4141 tgccttcctg tttttgctca cccagaaacg ctggtgaaag taaaagatgc tgaagatcag

4201 ttgggtgcac gagtgggtta catcgaactg gatctcaaca gcggtaagat ccttgagagt

4261 tttcgccccg aagaacgttt tccaatgatg agcactttta aagttctgct atgtggcgcg

4321 gtattatccc gtattgacgc cgggcaagag caactcggtc gccgcataca ctattctcag

4381 aatgacttgg ttgagtactc accagtcaca gaaaagcatc ttacggatgg catgacagta

4441 agagaattat gcagtgctgc cataaccatg agtgataaca ctgcggccaa cttacttctg

4501 acaacgatcg gaggaccgaa ggagctaacc gcttttttgc acaacatggg ggatcatgta

4561 actcgccttg atcgttggga accggagctg aatgaagcca taccaaacga cgagcgtgac

4621 accacgatgc ctgtagcaat ggcaacaacg ttgcgcaaac tattaactgg cgaactactt

4681 actctagctt cccggcaaca attaatagac tggatggagg cggataaagt tgcaggacca

4741 cttctgcgct cggcccttcc ggctggctgg tttattgctg ataaatctgg agccggtgag

4801 cgtgggtctc gcggtatcat tgcagcactg gggccagatg gtaagccctc ccgtatcgta

4861 gttatctaca cgacggggag tcaggcaact atggatgaac gaaatagaca gatcgctgag

4921 ataggtgcct cactgattaa gcattggtaa ctgtcagacc aagtttactc atatatactt

4981 tagattgatt taaaacttca tttttaattt aaaaggatct aggtgaagat cctttttgat

5041 aatctcatga ccaaaatccc ttaacgtgag ttttcgttcc actgagcgtc agaccccgta

5101 gaaaagatca aaggatcttc ttgagatcct ttttttctgc gcgtaatctg ctgcttgcaa

5161 acaaaaaaac caccgctacc agcggtggtt tgtttgccgg atcaagagct accaactctt

5221 tttccgaagg taactggctt cagcagagcg cagataccaa atactgtcct tctagtgtag

5281 ccgtagttag gccaccactt caagaactct gtagcaccgc ctacatacct cgctctgcta

5341 atcctgttac cagtggctgc tgccagtggc gataagtcgt gtcttaccgg gttggactca

5401 agacgatagt taccggataa ggcgcagcgg tcgggctgaa cggggggttc gtgcacacag

5461 cccagcttgg agcgaacgac ctacaccgaa ctgagatacc tacagcgtga gcattgagaa

5521 agcgccacgc ttcccgaagg gagaaaggcg gacaggtatc cggtaagcgg cagggtcgga

5581 acaggagagc gcacgaggga gcttccaggg ggaaacgcct ggtatcttta tagtcctgtc

5641 gggtttcgcc acctctgact tgagcgtcga tttttgtgat gctcgtcagg ggggcggagc

5701 ctatggaaaa acgccagcaa cgcggccttt ttacggttcc tggccttttg ctggcctttt

5761 gctcacatgt tctttcctgc gttatcccct gattctgtgg ataaccgtat taccgccttt

5821 gagtgagctg ataccgctcg ccgcagccga acgaccgagc gcagcgagtc agtgagcgag

5881 gaagcggaag agcgcccaat acgcaaaccg cctctccccg cgcgttggcc gattcattaa

5941 tgcagctggc acgacaggtt tcccgactgg aaagcgggca gtgagcgcaa cgcaattaat

6001 gtgagttagc tcactcatta ggcaccccag gctttacact ttatgcttcc ggctcgtatg

6061 ttgtgtggaa ttgtgagcgg ataacaattt cacacaggaa acagctatga ccatgattac

6121 gccaagcttg ataattcctg cagcccagct taattctttt cgagctcttt atgcttaagt

6181 ttacaattta atattcatac tttaagtatt ttttgtagta tcctagatat tgtgctttaa

6241 atgctcaccc ctcaaagcac cagtaatatt ttcatccact gaaataccat taaattttca

6301 aaaaaatact atgcatataa tgttatacat ataaacataa aacgccatgt aaatcaaaaa

6361 atatataaaa atatgtataa aaataaatat gcactaaata taagctaatt atgcataaaa

6421 attaaagtgc cctttattaa ctagctagtc gtaattattt atatttctat gttataaaaa

6481 aatcctcata taataatata attaatatat gtaatgtttt ttttatttta taattttaat

6541 ataaaataat atgtaaatta attcaaaaaa taaatataat tgttgtgaaa caaaaaacgt

6601 aattttttca tttgccttca aaatttaaat ttattttaat atttcctaaa atatatatac

6661 tttgtgtata aatatataaa aatatatatt tgcttataaa taaataaaaa attttataaa

6721 acataggggg atccatggtt ggttcgctaa actgcatcgt cgctgtgtcc cagaacatgg

6781 gcatcggcaa gaacggggac ctgccctggc caccgctcag gaacgaattt agatatttcc

6841 agagaatgac cacaacctct tcagtagaag gtaaacagaa tctggtgatt atgggtaaga

6901 agacctggtt ctccattcct gagaagaatc gacctttaaa gggtagaatt aatttagttc

6961 tcagcagaga actcaaggaa cctccacaag gagctcattt tctttccaga agtctagatg

7021 atgccttaaa acttactgaa caaccagaat tagcaaataa agtagacatg gtctggatag

7081 ttggtggcag ttctgtttat aaggaagcca tgaatcaccc aggccatctt aaactatttg

7141 tgacaaggat catgcaagac tttgaaagtg acacgttttt tccagaaatt gatttggaga

7201 aatataaact tctgccagaa tacccaggtg ttctctctga tgtccaggag gagaaaggca

7261 ttaagtacaa atttgaagta tatgagaaga atgattaagg atcccgtttt tcttacttat

7321 atatttatac caattgattg tatttataac tgtaaaaatg tgtatgttgt gtgcatattt

7381 ttttttgtgc atgcacatgc atgtaaatag ctaaaattat gaacatttta ttttttgttc

7441 agaaaaaaaa aactttacac acataaaatg gctagtatga atagccatat tttatataaa

7501 ttaaatccta tgaatttatg accatattaa aaatttagat atttatggaa cataatatgt

7561 ttgaaacaat aagacaaaat tattattatt attattattt ttactgttat aattatgttg

7621 tctcttcaat gattcataaa tagttggact tgatttttaa aatgtttata atatgattag

7681 catagttaaa taaaaaaagt tgaaaaatta aaaaaaaaca tataaacaca aatgatgttt

7741 tttccttcaa tttcgatg

//

>Py1115_PyWT-mScarlet

LOCUS pSL1115__final_ 7837 bp DNA circular 21-APR-2021

SOURCE

ORGANISM

COMMENT Lindner Lab - used to create Py1115 = Py17XNL WT-mScarlet

COMMENT ApEinfo:methylated:1

FEATURES Location/Qualifiers

exon 6814..7377

/vntifkey="61"

/locus_tag="hDHFR"

/label="hDHFR"

/ApEinfo_label="hDHFR"

/ApEinfo_fwdcolor="pink"

/ApEinfo_revcolor="pink"

/ApEinfo_graphicformat="arrow_data {{0 0.5 0 1 2 0 0 -1 0

-0.5} {} 0} width 5 offset 0"

misc_feature 1898..2471

/locus_tag="PbEF1a promoter"

/label="PbEF1a promoter"

/ApEinfo_label="PbEF1a promoter"

/ApEinfo_fwdcolor="cyan"

/ApEinfo_revcolor="green"

/ApEinfo_graphicformat="arrow_data {{0 0.5 0 1 2 0 0 -1 0

-0.5} {} 0} width 5 offset 0"

misc_feature 3280..3727

/locus_tag="PbDHFR/TS\3'UTR"

/label="PbDHFR/TS\3'UTR"

/ApEinfo_label="PbDHFR/TS\3'UTR"

/ApEinfo_fwdcolor="#ff0000"

/ApEinfo_revcolor="green"

/ApEinfo_graphicformat="arrow_data {{0 0.5 0 1 2 0 0 -1 0

-0.5} {} 0} width 5 offset 0"

rep_origin 5124..5806

/locus_tag="ColE1 origin"

/label="ColE1 origin"

/ApEinfo_label="ColE1 origin"

/ApEinfo_fwdcolor="gray50"

/ApEinfo_revcolor="gray50"

/ApEinfo_graphicformat="arrow_data {{0 0.5 0 1 2 0 0 -1 0

-0.5} {} 0} width 5 offset 0"

CDS 4367..5026

/locus_tag="AmpR"

/label="AmpR"

/ApEinfo_label="AmpR"

/ApEinfo_fwdcolor="yellow"

/ApEinfo_revcolor="yellow"

/ApEinfo_graphicformat="arrow_data {{0 0.5 0 1 2 0 0 -1 0

-0.5} {} 0} width 5 offset 0"

misc_feature 7384..7834

/locus_tag="PbDHFR-TS 3'UTR"

/label="PbDHFR-TS 3'UTR"

/ApEinfo_label="PbDHFR-TS 3'UTR"

/ApEinfo_fwdcolor="cyan"

/ApEinfo_revcolor="green"

/ApEinfo_graphicformat="arrow_data {{0 0.5 0 1 2 0 0 -1 0

-0.5} {} 0} width 5 offset 0"

misc_feature join(6210..6519,6524..6808)

/locus_tag="PbEF1a 5'UTR"

/label="PbEF1a 5'UTR"

/ApEinfo_label="PbEF1a 5'UTR"

/ApEinfo_fwdcolor="#804040"

/ApEinfo_revcolor="#804040"

/ApEinfo_graphicformat="arrow_data {{0 0.5 0 1 2 0 0 -1 0

-0.5} {} 0} width 5 offset 0"

misc_feature 7..918

/locus_tag="py p230p Homology Arm 1"

/label="py p230p Homology Arm 1"

/ApEinfo_label="py p230p Homology Arm 1"

/ApEinfo_fwdcolor="cyan"

/ApEinfo_revcolor="green"

/ApEinfo_graphicformat="arrow_data {{0 0.5 0 1 2 0 0 -1 0

-0.5} {} 0} width 5 offset 0"

misc_feature 925..1873

/locus_tag="py p230p Homology Arm 2"

/label="py p230p Homology Arm 2"

/ApEinfo_label="py p230p Homology Arm 2"

/ApEinfo_fwdcolor="cyan"

/ApEinfo_revcolor="green"

/ApEinfo_graphicformat="arrow_data {{0 0.5 0 1 2 0 0 -1 0

-0.5} {} 0} width 5 offset 0"

misc_feature 2481..3176

/locus_tag="mScarlet-N1"

/label="mScarlet-N1"

/ApEinfo_label="mScarlet-N1"

/ApEinfo_fwdcolor="cyan"

/ApEinfo_revcolor="green"

/ApEinfo_graphicformat="arrow_data {{0 1 2 0 0 -1} {} 0}

width 5 offset 0"

ORIGIN

1 ggtaccAGCT TTGTGTTTTA TTTGGATGTG CAACAATAGA TTTATGATTA ACCAATGGTA

61 AATCATTATA ATCATATATT TCATCTTCAT TTACATATTT TACTAAAACA GTTTTTACAA

121 TTTGTTCTGT TTCTTTATCT ACACAATGAC ATTCAAATTG ATATGATTTT GTTAATGTAT

181 AATTAAAACC AGCTAATGCA TATTCTGGAT TTTCAAATGA ATAGTTAATA ATATTTTTAG

241 TATTAATTTT ATAGGAATCA TGTAGATAGC CTTTTTCATA TACTTGAGTA AAACATTTTT

301 CTGGCATTTT TTTTGTATTT TCGGGGCATC TAAAACCAAA ACTTGTATTT GATTTGGAAA

361 TTATTATACA TAGATCTCCT TCACTATAAG CATTTAAATA ATTATATAAT TCACCTTTTC

421 TAAAATCACA AAATGAAACT GGTCTATCTT TTAAATCTGT TCGAACATGT AATAAAATAA

481 TTGCTCTTCT ATAATCAGTC CCACCATTTG TTACACCACA TTCTATGGTT AGTGGATATG

541 ATCCATGATT TATAAATGAG GTCATAAAAA ATTTAAAACC TTTAAATTGT TCATTTATTT

601 CTGGTATATC TATATCTTTT GTATTAAAAA ATAAATCTGT AAATTTATTT ATAGGAAATA

661 CATTACTTAA ATATTTTATG GTATTAATAG GTTCTTTACC ATATACGATT CTATTATCTG

721 TTATTTGCTC ACAATTATAC ATGAATAAAC TAAATTCATT TATTGTTTTT TCACATATTT

781 GTATTTCACT TAATTTGCTT AAATTATAAT CAAAATTACA CATCTGATAA GGTATATCGT

841 TAAAATATTT AATCGTTTTA CTATCATCAT CAACTATTTT TAAAATGTGA TCAGATGTTG

901 CCATTCCATC ACCAAATGgg gcccTATGGA ACTACATCTA TATAAGAGAT TTTTTTATTT

961 TTATATATTG GTTTTGAATC TAGATATTCA TAATTTTTAA TATATATTGT TCCTATATTT

1021 TTGGTTTTAT AATCTTCACA TGTACAATAA AAATCAAATT CTTTTTTTGT AATTTTTGAA

1081 ATCATTAAAA ATGTTGATGA ATTATTTATA AAATAATCTG CATCTATAAC TTTTATTTCA

1141 TTAAATATAT TTTCGATATT TTCAGTTATT TGTTCATTCA TGTTACTTTT TATATATACC

1201 TTTCTAAAAC AATTTTTTGG ATTAATTTTT CCAGTTGGAC AATTTAAGCT AACTACTATA

1261 TTTTCTTTTA ATTCGATTTC ACAAAATTCA TTTTGTTTTT CTAAATCATA AACATTGGAA

1321 TAGTAAAATT TTTTTTTAGA ATTACCTGAA AAATCACATC CATATAATTT ATTTTTTGTT

1381 GTATTTTGAG AAACCCTTAT CACTGTAATA CCTTTTTGAC CTTTCATATT ATTAATTATT

1441 GTTTTAGTAT TATCACAATA TATTTTCAAT ATTAATACTT TTTCTACAAT TAATGGAGTA

1501 ACAAAAGTGA AATTTCCTTT CTCTTTGAAC ATTTTATTTT TATCTAATAT GATAACACCT

1561 GGTAATATAT CATCTAATTT TTCTTTTTTG TCTAGATACA TTACTTCATC ATTTTCTGGT

1621 ATGTATTCTA TATTTTTATA TTTTTCTAAT TCTTCTGGTT CAACATTTGC ATTTTCTAAA

1681 TAAAAATTAT TTTTTTGACT ACTACGTTGT GATGTTTTCT TATTATTTAT TATGATATTA

1741 CTATCAAAAA TGTCATCATC ACTTTTTACA ATTTTGGGAC ATTTAACTTG AACCATATTC

1801 AGATTCTGTT CTAGATGTAT ATAACATGAA TGAACTGTAT CTACTTCATT AACGGGTTCA

1861 AGAGAATTCG GTAgccgcgg tggcggccgc TctcgagAGC TTAATTCTTT TCGAGCTCTT

1921 TATGCTTAAG TTTACAATTT AATATTCATA CTTTAAGTAT TTTTTGTAGT ATCCTAGATA

1981 TTGTGCTTTA AATGCTCACC CCTCAAAGCA CCAGTAATAT TTTCATCCAC TGAAATACCA

2041 TTAAATTTTC AAAAAAATAC TATGCATATA ATGTTATACA TATAAACATA AAACGCCATG

2101 TAAATCAAAA AATATATAAA AATATGTATA AAAATAAATA TGCACTAAAT ATAAGCTAAT

2161 TATGCATAAA AATTAAAGTG CCCTTTATTA ACTAGctagT CGTAATTATT TATATTTCTA

2221 TGTTATAAAA AAATCCTCAT ATAATAATAT AATTAATATA TGTAATGTTT TTTTTATTTT

2281 ATAATTTTAA TATAAAATAA TATGTAAATT AATTCAAAAA ATAAATATAA TTGTTGTGAA

2341 ACAAAAAACG TAATTTTTTC ATTTGCCTTC AAAATTTAAA TTTATTTTAA TATTTCCTAA

2401 AATATATATA CTTTGTGTAT AAATATATAA AAATATATAT TTGCTTATAA ATAAATAAAA

2461 ATTTTATAAA Aatgactagt GTGAGCAAGG GCGAGGCAGT GATCAAGGAG TTCATGCGGT

2521 TCAAGGTGCA CATGGAGGGC TCCATGAACG GCCACGAGTT CGAGATCGAG GGCGAGGGCG

2581 AGGGCCGCCC CTACGAGGGC ACCCAGACCG CCAAGCTGAA GGTGACCAAG GGTGGCCCCC

2641 TGCCCTTCTC CTGGGACATC CTGTCCCCTC AGTTCATGTA CGGCTCCAGG GCCTTCACCA

2701 AGCACCCCGC CGACATCCCC GACTACTATA AGCAGTCCTT CCCCGAGGGC TTCAAGTGGG

2761 AGCGCGTGAT GAACTTCGAG GACGGCGGCG CCGTGACCGT GACCCAGGAC ACCTCCCTGG

2821 AGGACGGCAC CCTGATCTAC AAGGTGAAGC TtCGCGGCAC CAACTTCCCT CCTGACGGCC

2881 CCGTAATGCA GAAGAAGACA ATGGGCTGGG AAGCATCCAC CGAGCGGTTG TACCCCGAGG

2941 ACGGCGTGCT GAAGGGCGAC ATTAAGATGG CCCTGCGCCT GAAGGACGGC GGtCGCTACC

3001 TGGCGGACTT CAAGACCACC TACAAGGCCA AGAAGCCCGT GCAGATGCCC GGCGCCTACA

3061 ACGTCGAtCG CAAGTTGGAC ATCACCTCCC ACAACGAGGA CTACACCGTG GTGGAACAGT

3121 ACGAACGCTC CGAGGGCCGC CACTCCACCG GCGGCATGGA CGAGCTGTAC AAGTAATTCG

3181 AAAGATCCCA ACGAAAAGAG AGACCACATG GTCCTTCTTG AGTTTGTAAC AGCTGCTGGG

3241 ATTACACATG GCATGGATGA ACTATACAAA TAAggatccG TTTTTCTTAC TTATATATTT

3301 ATACCAATTG ATTGTATTTA TAACTGTAAA AATGTGTATG TTGTGTGCAT ATTTTTTTTT

3361 GTGCATGCAC ATGCATGTAA ATAGCTAAAA TTATGAACAT TTTATTTTTT GTTCAGAAAA

3421 AAAAAACTTT ACACACATAA AATGGCTAGT ATGAATAGCC ATATTTTATA TAAATTAAAT

3481 CCTATGAATT TATGACCATA TTAAAAATTT AGATATTTAT GGAACATAAT ATGTTTGAAA

3541 CAATAAGACA AAATTATTAT TATTATTATT ATTTTTACTG TTATAATTAT GTTGTCTCTT

3601 CAATGATTCA TAAATAGTTG GACTTGATTT TTAAAATGTT TATAATATGA TTAGCATAGT

3661 TAAATAAAAA AAGTTGAAAA ATTAAAAAAA AACATATAAA CACAAATGAT GTTTTTTCCT

3721 TCAATTTcgg cgcctgatgc ggtattttct ccttacgcat ctgtgcggta tttcacaccg

3781 catatggtgc actctcagta caatctgctc tgatgccgca tagttaagcc agccccgaca

3841 cccgccaaca cccgctgacg cgccctgacg ggcttgtctg ctcccggcat ccgcttacag

3901 acaagctgtg accgtctccg ggagctgcat gtgtcagagg ttttcaccgt catcaccgaa

3961 acgcgcgaga cgaaagggcc tcgtgatacg cctattttta taggttaatg tcatgataat

4021 aatggtttct tagacgtcag gtggcacttt tcggggaaat gtgcgcggaa cccctatttg

4081 tttatttttc taaatacatt caaatatgta tccgctcatg agacaataac cctgataaat

4141 gcttcaataa tattgaaaaa ggaagagtat gagtattcaa catttccgtg tcgcccttat

4201 tccctttttt gcggcatttt gccttcctgt ttttgctcac ccagaaacgc tggtgaaagt

4261 aaaagatgct gaagatcagt tgggtgcacg agtgggttac atcgaactgg atctcaacag

4321 cggtaagatc cttgagagtt ttcgccccga agaacgtttt ccaatgatga gcacttttaa

4381 agttctgcta tgtggcgcgg tattatcccg tattgacgcc gggcaagagc aactcggtcg

4441 ccgcatacac tattctcaga atgacttggt tgagtactca ccagtcacag aaaagcatct

4501 tacggatggc atgacagtaa gagaattatg cagtgctgcc ataaccatga gtgataacac

4561 tgcggccaac ttacttctga caacgatcgg aggaccgaag gagctaaccg cttttttgca

4621 caacatgggg gatcatgtaa ctcgccttga tcgttgggaa ccggagctga atgaagccat

4681 accaaacgac gagcgtgaca ccacgatgcc tgtagcaatg gcaacaacgt tgcgcaaact

4741 attaactggc gaactactta ctctagcttc ccggcaacaa ttaatagact ggatggaggc

4801 ggataaagtt gcaggaccac ttctgcgctc ggcccttccg gctggctggt ttattgctga

4861 taaatctgga gccggtgagc gtgggtctcg cggtatcatt gcagcactgg ggccagatgg

4921 taagccctcc cgtatcgtag ttatctacac gacggggagt caggcaacta tggatgaacg

4981 aaatagacag atcgctgaga taggtgcctc actgattaag cattggtaac tgtcagacca

5041 agtttactca tatatacttt agattgattt aaaacttcat ttttaattta aaaggatcta

5101 ggtgaagatc ctttttgata atctcatgac caaaatccct taacgtgagt tttcgttcca

5161 ctgagcgtca gaccccgtag aaaagatcaa aggatcttct tgagatcctt tttttctgcg

5221 cgtaatctgc tgcttgcaaa caaaaaaacc accgctacca gcggtggttt gtttgccgga

5281 tcaagagcta ccaactcttt ttccgaaggt aactggcttc agcagagcgc agataccaaa

5341 tactgtcctt ctagtgtagc cgtagttagg ccaccacttc aagaactctg tagcaccgcc

5401 tacatacctc gctctgctaa tcctgttacc agtggctgct gccagtggcg ataagtcgtg

5461 tcttaccggg ttggactcaa gacgatagtt accggataag gcgcagcggt cgggctgaac

5521 ggggggttcg tgcacacagc ccagcttgga gcgaacgacc tacaccgaac tgagatacct

5581 acagcgtgag cattgagaaa gcgccacgct tcccgaaggg agaaaggcgg acaggtatcc

5641 ggtaagcggc agggtcggaa caggagagcg cacgagggag cttccagggg gaaacgcctg

5701 gtatctttat agtcctgtcg ggtttcgcca cctctgactt gagcgtcgat ttttgtgatg

5761 ctcgtcaggg gggcggagcc tatggaaaaa cgccagcaac gcggcctttt tacggttcct

5821 ggccttttgc tggccttttg ctcacatgtt ctttcctgcg ttatcccctg attctgtgga

5881 taaccgtatt accgcctttg agtgagctga taccgctcgc cgcagccgaa cgaccgagcg

5941 cagcgagtca gtgagcgagg aagcggaaga gcgcccaata cgcaaaccgc ctctccccgc

6001 gcgttggccg attcattaat gcagctggca cgacaggttt cccgactgga aagcgggcag

6061 tgagcgcaac gcaattaatg tgagttagct cactcattag gcaccccagg ctttacactt

6121 tatgcttccg gctcgtatgt tgtgtggaat tgtgagcgga taacaatttc acacaggaaa

6181 cagctatgac catgattacg ccaagcttga taattcctgc agcccagctt aattcttttc

6241 gagctcttta tgcttaagtt tacaatttaa tattcatact ttaagtattt tttgtagtat

6301 cctagatatt gtgctttaaa tgctcacccc tcaaagcacc agtaatattt tcatccactg

6361 aaataccatt aaattttcaa aaaaatacta tgcatataat gttatacata taaacataaa

6421 acgccatgta aatcaaaaaa tatataaaaa tatgtataaa aataaatatg cactaaatat

6481 aagctaatta tgcataaaaa ttaaagtgcc ctttattaac tagctagtcg taattattta

6541 tatttctatg ttataaaaaa atcctcatat aataatataa ttaatatatg taatgttttt

6601 tttattttat aattttaata taaaataata tgtaaattaa ttcaaaaaat aaatataatt

6661 gttgtgaaac aaaaaacgta attttttcat ttgccttcaa aatttaaatt tattttaata

6721 tttcctaaaa tatatatact ttgtgtataa atatataaaa atatatattt gcttataaat

6781 aaataaaaaa ttttataaaa cataggggga tccatggttg gttcgctaaa ctgcatcgtc

6841 gctgtgtccc agaacatggg catcggcaag aacggggacc tgccctggcc accgctcagg

6901 aacgaattta gatatttcca gagaatgacc acaacctctt cagtagaagg taaacagaat

6961 ctggtgatta tgggtaagaa gacctggttc tccattcctg agaagaatcg acctttaaag

7021 ggtagaatta atttagttct cagcagagaa ctcaaggaac ctccacaagg agctcatttt

7081 ctttccagaa gtctagatga tgccttaaaa cttactgaac aaccagaatt agcaaataaa

7141 gtagacatgg tctggatagt tggtggcagt tctgtttata aggaagccat gaatcaccca

7201 ggccatctta aactatttgt gacaaggatc atgcaagact ttgaaagtga cacgtttttt

7261 ccagaaattg atttggagaa atataaactt ctgccagaat acccaggtgt tctctctgat

7321 gtccaggagg agaaaggcat taagtacaaa tttgaagtat atgagaagaa tgattaagga

7381 tcccgttttt cttacttata tatttatacc aattgattgt atttataact gtaaaaatgt

7441 gtatgttgtg tgcatatttt tttttgtgca tgcacatgca tgtaaatagc taaaattatg

7501 aacattttat tttttgttca gaaaaaaaaa actttacaca cataaaatgg ctagtatgaa

7561 tagccatatt ttatataaat taaatcctat gaatttatga ccatattaaa aatttagata

7621 tttatggaac ataatatgtt tgaaacaata agacaaaatt attattatta ttattatttt

7681 tactgttata attatgttgt ctcttcaatg attcataaat agttggactt gatttttaaa

7741 atgtttataa tatgattagc atagttaaat aaaaaaagtt gaaaaattaa aaaaaaacat

7801 ataaacacaa atgatgtttt ttccttcaat ttcgatg

//

>Py1065_*pynot1-g^-^*

LOCUS pSL1065 6892 bp DNA circular 21-APR-2021

SOURCE

ORGANISM

COMMENT Lindner Lab - used to create Py1065 = Py17XNL deletion of pynot1-g

COMMENT ApEinfo:methylated:1

FEATURES Location/Qualifiers

exon 5869..6432

/vntifkey="61"

/locus_tag="hDHFR"

/label="hDHFR"

/ApEinfo_label="hDHFR"

/ApEinfo_fwdcolor="pink"

/ApEinfo_revcolor="pink"

/ApEinfo_graphicformat="arrow_data {{0 1 2 0 0 -1} {} 0}

width 5 offset 0"

misc_feature 1650..2328

/locus_tag="GFPmut2"

/label="GFPmut2"

/ApEinfo_label="GFPmut2"

/ApEinfo_fwdcolor="cyan"

/ApEinfo_revcolor="green"

/ApEinfo_graphicformat="arrow_data {{0 1 2 0 0 -1} {} 0}

width 5 offset 0"

misc_feature 2335..2782

/locus_tag="PbDHFR/TS\3'UTR"

/label="PbDHFR/TS\3'UTR"

/ApEinfo_label="PbDHFR/TS\3'UTR"

/ApEinfo_fwdcolor="#ff0000"

/ApEinfo_revcolor="green"

/ApEinfo_graphicformat="arrow_data {{0 1 2 0 0 -1} {} 0}

width 5 offset 0"

rep_origin 4179..4861

/locus_tag="ColE1 origin"

/label="ColE1 origin"

/ApEinfo_label="ColE1 origin"

/ApEinfo_fwdcolor="gray50"

/ApEinfo_revcolor="gray50"

/ApEinfo_graphicformat="arrow_data {{0 1 2 0 0 -1} {} 0}

width 5 offset 0"

CDS 3422..4081

/locus_tag="AmpR"

/label="AmpR"

/ApEinfo_label="AmpR"

/ApEinfo_fwdcolor="yellow"

/ApEinfo_revcolor="yellow"

/ApEinfo_graphicformat="arrow_data {{0 1 2 0 0 -1} {} 0}

width 5 offset 0"

misc_feature join(5265..5574,5579..5863)

/locus_tag="PbEF1a 5'UTR"

/label="PbEF1a 5'UTR"

/ApEinfo_label="PbEF1a 5'UTR"

/ApEinfo_fwdcolor="#804040"

/ApEinfo_revcolor="#804040"

/ApEinfo_graphicformat="arrow_data {{0 1 2 0 0 -1} {} 0}

width 5 offset 0"

misc_feature 6440..6887

/locus_tag="PbDHFR-TS 3'UTR"

/label="PbDHFR-TS 3'UTR"

/ApEinfo_label="PbDHFR-TS 3'UTR"

/ApEinfo_fwdcolor="cyan"

/ApEinfo_revcolor="green"

/ApEinfo_graphicformat="arrow_data {{0 1 2 0 0 -1} {} 0}

width 5 offset 0"

misc_feature 748..1608

/locus_tag="PyNOT1-G 5' Homology Arm"

/label="PyNOT1-G 5' Homology Arm"

/ApEinfo_label="PyNOT1-G 5' Homology Arm"

/ApEinfo_fwdcolor="cyan"

/ApEinfo_revcolor="green"

/ApEinfo_graphicformat="arrow_data {{0 0.5 0 1 2 0 0 -1 0

-0.5} {} 0} width 5 offset 0"

misc_feature 7..730

/locus_tag="PyNOT1-G 3' Homology Arm"

/label="PyNOT1-G 3' Homology Arm"

/ApEinfo_label="PyNOT1-G 3' Homology Arm"

/ApEinfo_fwdcolor="cyan"

/ApEinfo_revcolor="green"

/ApEinfo_graphicformat="arrow_data {{0 0.5 0 1 2 0 0 -1 0

-0.5} {} 0} width 5 offset 0"

ORIGIN

1 ggtaccCAAC TCATACAAAC TCTTCTTAGA ATGTAATATA TATGTACATG TATATATATT

61 ATTATTATTA TTATTTTATT TTTTTTTTTT TTTTTTTTTA CTTACACATG TATGTATGCA

121 CATACATATA TTACAATTTT ATACCATTTT AAAATTAGTC TTATTCATCA TCGAAGAAAA

181 ATTTATTATC CTTTGCATAC ATACATACAT ACATATATAT ATATATATAT ACATATATAT

241 ATACATGCAT TTATTTTACT ATTTTTTTAC AATTTATAAT TGTATTTTTA AAATAGCAGT

301 TTTGTAATTT TTTTCAAATT CATATTTTTT TTTTCCCATT TTTTTGTTAA ACTTTTAAAA

361 CACAACAATA GTATCGATAG TACTAGTAGT ATATAATTAT AAAATGCGTT TATTTATTTT

421 TTCTACATTT CTACAATTTT TCTACAATTT TCCTACAATT TTAAAATACC ACATATAAAC

481 CTCAATACAC ACATCCATTC CGTTTTAATA AAAAAAAAAA TATATATTTC CGAAATAACG

541 CTAGGAGGAA AATAAAATTC CCCTGATTTT TACTATCATT TTAATGTTTT CATTATTCTA

601 TATCTTTAAT AATATACATG AAATGTAATT TTTATGTGTG CAAAAATATT TCAAGTTTAT

661 TATTAAGCAA AAAATTTACA AAAATATATA CACATATGAG AAATATGAAT CCCGCTTTGT

721 CTATCCATAC ggcggccgcg cgggcccCCT AGTTTATTTA TCTACAAACG TATGCCCACA

781 ATTTCAACTA TACAATGTGT TATATGTATA ATAAATATTT GTATACAATA TATACTTATT

841 AATATAATAC AATATATATA TATATATACA TAATTAATAT ATTTTTAGTG CATATAAATG

901 TACTTATTTA ATTCGTAAAA ATATTGCTAA TTAAACCCCA ATATTGTATA CATATACTAA

961 GGACATAAAT TCCTCTACGG TTTTATTCAC ATGCATTAAA AAAATATAAC TTAAGTATAT

1021 TTTTTCTGTA TATCCCCAAA AAAAAAAATG TGGTCAAAAA AAATATTATA TATATATAAT

1081 TAATTTTATA GGAAAAAAAC ATTCCCAATA AAATTAAAAA AAATTCTCAA TTTTTTTAAT

1141 ATTATTTTCC TTTTTGGTGA TATTCATATA TAGTTAATGA ATATAACATA ACACCAATTT

1201 ATAATCCTGC GTTTACTTTA TATGTAAAGT TTTCATAAAA TCATAATAAC TACGCAGTTA

1261 TAATAAGTTA AAAAGGGAAA ATGAAAAGAA AAAAAATATA TTTTTCTATA TGTGTGTGCA

1321 TATATATTGT ATATATATAT TTATAATCAT AAACAAAATA AGGCAAAAAA AAATACCAAA

1381 AAAAAATAAT AAAAAATAAA ATAAATAAAT AACCTCCAAA AAAATAGAAA ACAATCCAAA

1441 CAAGCCAAAC ATACCAAACA TACCAAACTT AGCAATCACA GCTGAAATTA AAAAAATAAC

1501 AAACACACCA AATCGTGTAA ACGCAAAAAA TACCACATAA CAAAACACAG TCAAATACCC

1561 ATATATATTG TTCATACCAT ATTGAGGTAA TTACAATAAA ACATGTGCcc tagtagtaaa

1621 ggagaagaac ttttcactgg agttgtcccA ATTCTTGTTG AATTAGATGG TGATGTTAAT

1681 GGGCACAAAT TTTCTGTCAG TGGAGAGGGT GAAGGTGATG CAACATACGG AAAACTTACC

1741 CTTAAATTTA TTTGCACTAC TGGAAAACTA CCTGTTCCAT GGCCAACACT TGTCACTACT

1801 TTCGCGTATG GTCTTCAATG CTTTGCGAGA TACCCAGATC ATATGAAACA GCATGACTTT

1861 TTCAAGAGTG CCATGCCCGA AGGTTATGTA CAGGAAAGAA CTATATTTTT CAAAGATGAC

1921 GGGAACTACA AGACACGTGC TGAAGTCAAG TTTGAAGGTG ATACCCTTGT TAATAGAATC

1981 GAGTTAAAAG GTATTGATTT TAAAGAAGAT GGAAACATTC TTGGACACAA ATTGGAATAC

2041 AACTATAACT CACACAATGT ATACATCATG GCAGACAAAC AAAAGAATGG AATCAAAGTT

2101 AACTTCAAAA TTAGACACAA CATTGAAGAT GGAAGCGTTC AACTAGCAGA CCATTATCAA

2161 CAAAATACTC CAATTGGCGA TGGCCCTGTC CTTTTACCAG ACAACCATTA CCTGTCCACA

2221 CAATCTGCCC TTTCGAAAGA TCCCAACGAA AAGAGAGACC ACATGGTCCT TCTTGAGTTT

2281 GTAACAGCTG CTGGGATTAC ACATGGCATG GATGAACTAT ACAAATAAgg atccGTTTTT

2341 CTTACTTATA TATTTATACC AATTGATTGT ATTTATAACT GTAAAAATGT GTATGTTGTG

2401 TGCATATTTT TTTTTGTGCA TGCACATGCA TGTAAATAGC TAAAATTATG AACATTTTAT

2461 TTTTTGTTCA GAAAAAAAAA ACTTTACACA CATAAAATGG CTAGTATGAA TAGCCATATT

2521 TTATATAAAT TAAATCCTAT GAATTTATGA CCATATTAAA AATTTAGATA TTTATGGAAC

2581 ATAATATGTT TGAAACAATA AGACAAAATT ATTATTATTA TTATTATTTT TACTGTTATA

2641 ATTATGTTGT CTCTTCAATG ATTCATAAAT AGTTGGACTT GATTTTTAAA ATGTTTATAA

2701 TATGATTAGC ATAGTTAAAT AAAAAAAGTT GAAAAATTAA AAAAAAACAT ATAAACACAA

2761 ATGATGTTTT TTCCTTCAAT TTcggcgcct gatgcggtat tttctcctta cgcatctgtg

2821 cggtatttca caccgcatat ggtgcactct cagtacaatc tgctctgatg ccgcatagtt

2881 aagccagccc cgacacccgc caacacccgc tgacgcgccc tgacgggctt gtctgctccc

2941 ggcatccgct tacagacaag ctgtgaccgt ctccgggagc tgcatgtgtc agaggttttc

3001 accgtcatca ccgaaacgcg cgagacgaaa gggcctcgtg atacgcctat ttttataggt

3061 taatgtcatg ataataatgg tttcttagac gtcaggtggc acttttcggg gaaatgtgcg

3121 cggaacccct atttgtttat ttttctaaat acattcaaat atgtatccgc tcatgagaca

3181 ataaccctga taaatgcttc aataatattg aaaaaggaag agtatgagta ttcaacattt

3241 ccgtgtcgcc cttattccct tttttgcggc attttgcctt cctgtttttg ctcacccaga

3301 aacgctggtg aaagtaaaag atgctgaaga tcagttgggt gcacgagtgg gttacatcga

3361 actggatctc aacagcggta agatccttga gagttttcgc cccgaagaac gttttccaat

3421 gatgagcact tttaaagttc tgctatgtgg cgcggtatta tcccgtattg acgccgggca

3481 agagcaactc ggtcgccgca tacactattc tcagaatgac ttggttgagt actcaccagt

3541 cacagaaaag catcttacgg atggcatgac agtaagagaa ttatgcagtg ctgccataac

3601 catgagtgat aacactgcgg ccaacttact tctgacaacg atcggaggac cgaaggagct

3661 aaccgctttt ttgcacaaca tgggggatca tgtaactcgc cttgatcgtt gggaaccgga

3721 gctgaatgaa gccataccaa acgacgagcg tgacaccacg atgcctgtag caatggcaac

3781 aacgttgcgc aaactattaa ctggcgaact acttactcta gcttcccggc aacaattaat

3841 agactggatg gaggcggata aagttgcagg accacttctg cgctcggccc ttccggctgg

3901 ctggtttatt gctgataaat ctggagccgg tgagcgtggg tctcgcggta tcattgcagc

3961 actggggcca gatggtaagc cctcccgtat cgtagttatc tacacgacgg ggagtcaggc

4021 aactatggat gaacgaaata gacagatcgc tgagataggt gcctcactga ttaagcattg

4081 gtaactgtca gaccaagttt actcatatat actttagatt gatttaaaac ttcattttta

4141 atttaaaagg atctaggtga agatcctttt tgataatctc atgaccaaaa tcccttaacg

4201 tgagttttcg ttccactgag cgtcagaccc cgtagaaaag atcaaaggat cttcttgaga

4261 tccttttttt ctgcgcgtaa tctgctgctt gcaaacaaaa aaaccaccgc taccagcggt

4321 ggtttgtttg ccggatcaag agctaccaac tctttttccg aaggtaactg gcttcagcag

4381 agcgcagata ccaaatactg tccttctagt gtagccgtag ttaggccacc acttcaagaa

4441 ctctgtagca ccgcctacat acctcgctct gctaatcctg ttaccagtgg ctgctgccag

4501 tggcgataag tcgtgtctta ccgggttgga ctcaagacga tagttaccgg ataaggcgca

4561 gcggtcgggc tgaacggggg gttcgtgcac acagcccagc ttggagcgaa cgacctacac

4621 cgaactgaga tacctacagc gtgagcattg agaaagcgcc acgcttcccg aagggagaaa

4681 ggcggacagg tatccggtaa gcggcagggt cggaacagga gagcgcacga gggagcttcc

4741 agggggaaac gcctggtatc tttatagtcc tgtcgggttt cgccacctct gacttgagcg

4801 tcgatttttg tgatgctcgt caggggggcg gagcctatgg aaaaacgcca gcaacgcggc

4861 ctttttacgg ttcctggcct tttgctggcc ttttgctcac atgttctttc ctgcgttatc

4921 ccctgattct gtggataacc gtattaccgc ctttgagtga gctgataccg ctcgccgcag

4981 ccgaacgacc gagcgcagcg agtcagtgag cgaggaagcg gaagagcgcc caatacgcaa

5041 accgcctctc cccgcgcgtt ggccgattca ttaatgcagc tggcacgaca ggtttcccga

5101 ctggaaagcg ggcagtgagc gcaacgcaat taatgtgagt tagctcactc attaggcacc

5161 ccaggcttta cactttatgc ttccggctcg tatgttgtgt ggaattgtga gcggataaca

5221 atttcacaca ggaaacagct atgaccatga ttacgccaag cttgataatt cctgcagccc

5281 agcttaattc ttttcgagct ctttatgctt aagtttacaa tttaatattc atactttaag

5341 tattttttgt agtatcctag atattgtgct ttaaatgctc acccctcaaa gcaccagtaa

5401 tattttcatc cactgaaata ccattaaatt ttcaaaaaaa tactatgcat ataatgttat

5461 acatataaac ataaaacgcc atgtaaatca aaaaatatat aaaaatatgt ataaaaataa

5521 atatgcacta aatataagct aattatgcat aaaaattaaa gtgcccttta ttaactagct

5581 agtcgtaatt atttatattt ctatgttata aaaaaatcct catataataa tataattaat

5641 atatgtaatg ttttttttat tttataattt taatataaaa taatatgtaa attaattcaa

5701 aaaataaata taattgttgt gaaacaaaaa acgtaatttt ttcatttgcc ttcaaaattt

5761 aaatttattt taatatttcc taaaatatat atactttgtg tataaatata taaaaatata

5821 tatttgctta taaataaata aaaaatttta taaaacatag ggggatccat ggttggttcg

5881 ctaaactgca tcgtcgctgt gtcccagaac atgggcatcg gcaagaacgg ggacctgccc

5941 tggccaccgc tcaggaacga atttagatat ttccagagaa tgaccacaac ctcttcagta

6001 gaaggtaaac agaatctggt gattatgggt aagaagacct ggttctccat tcctgagaag

6061 aatcgacctt taaagggtag aattaattta gttctcagca gagaactcaa ggaacctcca

6121 caaggagctc attttctttc cagaagtcta gatgatgcct taaaacttac tgaacaacca

6181 gaattagcaa ataaagtaga catggtctgg atagttggtg gcagttctgt ttataaggaa

6241 gccatgaatc acccaggcca tcttaaacta tttgtgacaa ggatcatgca agactttgaa

6301 agtgacacgt tttttccaga aattgatttg gagaaatata aacttctgcc agaataccca

6361 ggtgttctct ctgatgtcca ggaggagaaa ggcattaagt acaaatttga agtatatgag

6421 aagaatgatt aaggatcccg tttttcttac ttatatattt ataccaattg attgtattta

6481 taactgtaaa aatgtgtatg ttgtgtgcat attttttttt gtgcatgcac atgcatgtaa

6541 atagctaaaa ttatgaacat tttatttttt gttcagaaaa aaaaaacttt acacacataa

6601 aatggctagt atgaatagcc atattttata taaattaaat cctatgaatt tatgaccata

6661 ttaaaaattt agatatttat ggaacataat atgtttgaaa caataagaca aaattattat

6721 tattattatt atttttactg ttataattat gttgtctctt caatgattca taaatagttg

6781 gacttgattt ttaaaatgtt tataatatga ttagcatagt taaataaaaa aagttgaaaa

6841 attaaaaaaa aacatataaa cacaaatgat gttttttcct tcaatttcga tg

//

>Py1061_*pynot1^-^*

LOCUS pSL1061__final_ 6929 bp DNA circular 21-APR-2021

SOURCE

ORGANISM

COMMENT Lindner Lab - used to create Py1061 = Py17XNL deletion of pynot1

COMMENT ApEinfo:methylated:1

FEATURES Location/Qualifiers

exon 5163..5726

/vntifkey="61"

/locus_tag="hDHFR"

/label="hDHFR"

/ApEinfo_label="hDHFR"

/ApEinfo_fwdcolor="pink"

/ApEinfo_revcolor="pink"

/ApEinfo_graphicformat="arrow_data {{0 1 2 0 0 -1} {} 0}

width 5 offset 0"

misc_feature 944..1622

/locus_tag="GFPmut2"

/label="GFPmut2"

/ApEinfo_label="GFPmut2"

/ApEinfo_fwdcolor="cyan"

/ApEinfo_revcolor="green"

/ApEinfo_graphicformat="arrow_data {{0 1 2 0 0 -1} {} 0}

width 5 offset 0"

misc_feature 5733..6183

/vntifkey="21"

/locus_tag="PbDHFR/TS\3'UTR"

/label="PbDHFR/TS\3'UTR"

/ApEinfo_label="PbDHFR/TS\3'UTR"

/ApEinfo_fwdcolor="#eb0214"

/ApEinfo_revcolor="#eb0214"

/ApEinfo_graphicformat="arrow_data {{0 1 2 0 0 -1} {} 0}

width 5 offset 0"

misc_feature 1629..2076

/locus_tag="PbDHFR/TS\3'UTR(1)"

/label="PbDHFR/TS\3'UTR(1)"

/ApEinfo_label="PbDHFR/TS\3'UTR"

/ApEinfo_fwdcolor="#ff0000"

/ApEinfo_revcolor="green"

/ApEinfo_graphicformat="arrow_data {{0 1 2 0 0 -1} {} 0}

width 5 offset 0"

misc_feature 45..878

/locus_tag="pynot1 - 5' Homology Arm (KO)"

/label="pynot1 - 5' Homology Arm (KO)"

/ApEinfo_label="pynot1 - 5' Homology Arm (KO)"

/ApEinfo_fwdcolor="cyan"

/ApEinfo_revcolor="green"

/ApEinfo_graphicformat="arrow_data {{0 1 2 0 0 -1} {} 0}

width 5 offset 0"

rep_origin 3473..4155

/locus_tag="ColE1 origin"

/label="ColE1 origin"

/ApEinfo_label="ColE1 origin"

/ApEinfo_fwdcolor="gray50"

/ApEinfo_revcolor="gray50"

/ApEinfo_graphicformat="arrow_data {{0 1 2 0 0 -1} {} 0}

width 5 offset 0"

CDS 2716..3375

/locus_tag="AmpR"

/label="AmpR"

/ApEinfo_label="AmpR"

/ApEinfo_fwdcolor="yellow"

/ApEinfo_revcolor="yellow"

/ApEinfo_graphicformat="arrow_data {{0 1 2 0 0 -1} {} 0}

width 5 offset 0"

misc_feature join(4559..4868,4873..5157)

/locus_tag="PbEF1a 5'UTR"

/label="PbEF1a 5'UTR"

/ApEinfo_label="PbEF1a 5'UTR"

/ApEinfo_fwdcolor="#804040"

/ApEinfo_revcolor="#804040"

/ApEinfo_graphicformat="arrow_data {{0 1 2 0 0 -1} {} 0}

width 5 offset 0"

misc_feature 6193..6912

/locus_tag="pynot1 - 3' homology arm (KO)"

/label="pynot1 - 3' homology arm (KO)"

/ApEinfo_label="pynot1 - 3' homology arm (KO)"

/ApEinfo_fwdcolor="cyan"

/ApEinfo_revcolor="green"

/ApEinfo_graphicformat="arrow_data {{0 0.5 0 1 2 0 0 -1 0

-0.5} {} 0} width 5 offset 0"

ORIGIN

1 CCAACTTGCC CCCCCAATAA CAGTTATATA TATGTACCAA AAAACAACCT AGAATTTACT

61 TAAATTTCCA TACCAATTTA ATATATTGTA TAAATGAATA CAAAGAAAAA ATGGAAAAAA

121 ATACGACTTA AAAGGTATAG AAACGCCGAA TTCTCAAACT GTATATATGG ATATTATGCA

181 CATACAATTA TTATACTATG AACCAATATA TTATATATAT ATATATATAT AATATATATA

241 TATAATGAGT GCATATGATA AATATACTTA ATAAACATAA CCAGAAAACA TATTCTCTTA

301 CCATTTTAAA GAAATTCACA CATTAAATTT GTATATATAT TAAATATATT ATATAATATA

361 TTATATTATA TATATTAATA TTGTAAATAT TTATTTTGCC ATATATTATA TTAGCTTATA

421 TGTAATTACG TACAGTATAG CGCAACGAGT ATTTGAATAA ATTTTTCTTT TAATCAACCT

481 TATTATATTT TTTCATAGAT AATTTTTTTA TTTTTTATTT CCTTTTCATT GATATATTTT

541 ATTATTTCAT TTTTATGTAT GTATATATAT ATATAATAAA TAGTTTAAAC AAAAATTGTA

601 TTCAATATAT ATGCATATAT AATAATTATT TATAATTTTT TTTTAACAAG TTTTTAAACA

661 GTTAAATCCG TATTTAGTAT ATATGTTTTT TTAATATATA TAAGTATATG TATGTGTTAT

721 GTATATATAT ATTTTAAATA TGTCACAATA TTTTTAAAAC ACACAATTAT AGGATTTATA

781 TAAAAATAAA ATAAGTAACA AAAGAGAAGA TATAACGCAT TTATGTACAT ATAATATATT

841 TATCGTTGTA CAATTGTAAA TATGAATTTT TACATATCAA AATATGAGAA TGGATTACCA

901 TGcctagtag taaaggagaa gaacttttca ctggagttgt cccAATTCTT GTTGAATTAG

961 ATGGTGATGT TAATGGGCAC AAATTTTCTG TCAGTGGAGA GGGTGAAGGT GATGCAACAT

1021 ACGGAAAACT TACCCTTAAA TTTATTTGCA CTACTGGAAA ACTACCTGTT CCATGGCCAA

1081 CACTTGTCAC TACTTTCGCG TATGGTCTTC AATGCTTTGC GAGATACCCA GATCATATGA

1141 AACAGCATGA CTTTTTCAAG AGTGCCATGC CCGAAGGTTA TGTACAGGAA AGAACTATAT

1201 TTTTCAAAGA TGACGGGAAC TACAAGACAC GTGCTGAAGT CAAGTTTGAA GGTGATACCC

1261 TTGTTAATAG AATCGAGTTA AAAGGTATTG ATTTTAAAGA AGATGGAAAC ATTCTTGGAC

1321 ACAAATTGGA ATACAACTAT AACTCACACA ATGTATACAT CATGGCAGAC AAACAAAAGA

1381 ATGGAATCAA AGTTAACTTC AAAATTAGAC ACAACATTGA AGATGGAAGC GTTCAACTAG

1441 CAGACCATTA TCAACAAAAT ACTCCAATTG GCGATGGCCC TGTCCTTTTA CCAGACAACC

1501 ATTACCTGTC CACACAATCT GCCCTTTCGA AAGATCCCAA CGAAAAGAGA GACCACATGG

1561 TCCTTCTTGA GTTTGTAACA GCTGCTGGGA TTACACATGG CATGGATGAA CTATACAAAT

1621 AAggatccGT TTTTCTTACT TATATATTTA TACCAATTGA TTGTATTTAT AACTGTAAAA

1681 ATGTGTATGT TGTGTGCATA TTTTTTTTTG TGCATGCACA TGCATGTAAA TAGCTAAAAT

1741 TATGAACATT TTATTTTTTG TTCAGAAAAA AAAAACTTTA CACACATAAA ATGGCTAGTA

1801 TGAATAGCCA TATTTTATAT AAATTAAATC CTATGAATTT ATGACCATAT TAAAAATTTA

1861 GATATTTATG GAACATAATA TGTTTGAAAC AATAAGACAA AATTATTATT ATTATTATTA

1921 TTTTTACTGT TATAATTATG TTGTCTCTTC AATGATTCAT AAATAGTTGG ACTTGATTTT

1981 TAAAATGTTT ATAATATGAT TAGCATAGTT AAATAAAAAA AGTTGAAAAA TTAAAAAAAA

2041 ACATATAAAC ACAAATGATG TTTTTTCCTT CAATTTcggc gcctgatgcg gtattttctc

2101 cttacgcatc tgtgcggtat ttcacaccgc atatggtgca ctctcagtac aatctgctct

2161 gatgccgcat agttaagcca gccccgacac ccgccaacac ccgctgacgc gccctgacgg

2221 gcttgtctgc tcccggcatc cgcttacaga caagctgtga ccgtctccgg gagctgcatg

2281 tgtcagaggt tttcaccgtc atcaccgaaa cgcgcgagac gaaagggcct cgtgatacgc

2341 ctatttttat aggttaatgt catgataata atggtttctt agacgtcagg tggcactttt

2401 cggggaaatg tgcgcggaac ccctatttgt ttatttttct aaatacattc aaatatgtat

2461 ccgctcatga gacaataacc ctgataaatg cttcaataat attgaaaaag gaagagtatg

2521 agtattcaac atttccgtgt cgcccttatt cccttttttg cggcattttg ccttcctgtt

2581 tttgctcacc cagaaacgct ggtgaaagta aaagatgctg aagatcagtt gggtgcacga

2641 gtgggttaca tcgaactgga tctcaacagc ggtaagatcc ttgagagttt tcgccccgaa

2701 gaacgttttc caatgatgag cacttttaaa gttctgctat gtggcgcggt attatcccgt

2761 attgacgccg ggcaagagca actcggtcgc cgcatacact attctcagaa tgacttggtt

2821 gagtactcac cagtcacaga aaagcatctt acggatggca tgacagtaag agaattatgc

2881 agtgctgcca taaccatgag tgataacact gcggccaact tacttctgac aacgatcgga

2941 ggaccgaagg agctaaccgc ttttttgcac aacatggggg atcatgtaac tcgccttgat

3001 cgttgggaac cggagctgaa tgaagccata ccaaacgacg agcgtgacac cacgatgcct

3061 gtagcaatgg caacaacgtt gcgcaaacta ttaactggcg aactacttac tctagcttcc

3121 cggcaacaat taatagactg gatggaggcg gataaagttg caggaccact tctgcgctcg

3181 gcccttccgg ctggctggtt tattgctgat aaatctggag ccggtgagcg tgggtctcgc

3241 ggtatcattg cagcactggg gccagatggt aagccctccc gtatcgtagt tatctacacg

3301 acggggagtc aggcaactat ggatgaacga aatagacaga tcgctgagat aggtgcctca

3361 ctgattaagc attggtaact gtcagaccaa gtttactcat atatacttta gattgattta

3421 aaacttcatt tttaatttaa aaggatctag gtgaagatcc tttttgataa tctcatgacc

3481 aaaatccctt aacgtgagtt ttcgttccac tgagcgtcag accccgtaga aaagatcaaa

3541 ggatcttctt gagatccttt ttttctgcgc gtaatctgct gcttgcaaac aaaaaaacca

3601 ccgctaccag cggtggtttg tttgccggat caagagctac caactctttt tccgaaggta

3661 actggcttca gcagagcgca gataccaaat actgtccttc tagtgtagcc gtagttaggc

3721 caccacttca agaactctgt agcaccgcct acatacctcg ctctgctaat cctgttacca

3781 gtggctgctg ccagtggcga taagtcgtgt cttaccgggt tggactcaag acgatagtta

3841 ccggataagg cgcagcggtc gggctgaacg gggggttcgt gcacacagcc cagcttggag

3901 cgaacgacct acaccgaact gagataccta cagcgtgagc attgagaaag cgccacgctt

3961 cccgaaggga gaaaggcgga caggtatccg gtaagcggca gggtcggaac aggagagcgc

4021 acgagggagc ttccaggggg aaacgcctgg tatctttata gtcctgtcgg gtttcgccac

4081 ctctgacttg agcgtcgatt tttgtgatgc tcgtcagggg ggcggagcct atggaaaaac

4141 gccagcaacg cggccttttt acggttcctg gccttttgct ggccttttgc tcacatgttc

4201 tttcctgcgt tatcccctga ttctgtggat aaccgtatta ccgcctttga gtgagctgat

4261 accgctcgcc gcagccgaac gaccgagcgc agcgagtcag tgagcgagga agcggaagag

4321 cgcccaatac gcaaaccgcc tctccccgcg cgttggccga ttcattaatg cagctggcac

4381 gacaggtttc ccgactggaa agcgggcagt gagcgcaacg caattaatgt gagttagctc

4441 actcattagg caccccaggc tttacacttt atgcttccgg ctcgtatgtt gtgtggaatt

4501 gtgagcggat aacaatttca cacaggaaac agctatgacc atgattacgc caagcttgat

4561 aattcctgca gcccagctta attcttttcg agctctttat gcttaagttt acaatttaat

4621 attcatactt taagtatttt ttgtagtatc ctagatattg tgctttaaat gctcacccct

4681 caaagcacca gtaatatttt catccactga aataccatta aattttcaaa aaaatactat

4741 gcatataatg ttatacatat aaacataaaa cgccatgtaa atcaaaaaat atataaaaat

4801 atgtataaaa ataaatatgc actaaatata agctaattat gcataaaaat taaagtgccc

4861 tttattaact agctagtcgt aattatttat atttctatgt tataaaaaaa tcctcatata

4921 ataatataat taatatatgt aatgtttttt ttattttata attttaatat aaaataatat

4981 gtaaattaat tcaaaaaata aatataattg ttgtgaaaca aaaaacgtaa ttttttcatt

5041 tgccttcaaa atttaaattt attttaatat ttcctaaaat atatatactt tgtgtataaa

5101 tatataaaaa tatatatttg cttataaata aataaaaaat tttataaaac atagggggat

5161 ccatggttgg ttcgctaaac tgcatcgtcg ctgtgtccca gaacatgggc atcggcaaga

5221 acggggacct gccctggcca ccgctcagga acgaatttag atatttccag agaatgacca

5281 caacctcttc agtagaaggt aaacagaatc tggtgattat gggtaagaag acctggttct

5341 ccattcctga gaagaatcga cctttaaagg gtagaattaa tttagttctc agcagagaac

5401 tcaaggaacc tccacaagga gctcattttc tttccagaag tctagatgat gccttaaaac

5461 ttactgaaca accagaatta gcaaataaag tagacatggt ctggatagtt ggtggcagtt

5521 ctgtttataa ggaagccatg aatcacccag gccatcttaa actatttgtg acaaggatca

5581 tgcaagactt tgaaagtgac acgttttttc cagaaattga tttggagaaa tataaacttc

5641 tgccagaata cccaggtgtt ctctctgatg tccaggagga gaaaggcatt aagtacaaat

5701 ttgaagtata tgagaagaat gattaaggat cccgtttttc ttacttatat atttatacca

5761 attgattgta tttataactg taaaaatgtg tatgttgtgt gcatattttt ttttgtgcat

5821 gcacatgcat gtaaatagct aaaattatga acattttatt ttttgttcag aaaaaaaaaa

5881 ctttacacac ataaaatggc tagtatgaat agccatattt tatataaatt aaatcctatg

5941 aatttatgac catattaaaa atttagatat ttatggaaca taatatgttt gaaacaataa

6001 gacaaaatta ttattattat tattattttt actgttataa ttatgttgtc tcttcaatga

6061 ttcataaata gttggacttg atttttaaaa tgtttataat atgattagca tagttaaata

6121 aaaaaagttg aaaaattaaa aaaaaacata taaacacaaa tgatgttttt tccttcaatt

6181 tcgatgggta ccCATATTGT TATTTTTAGA ATATTTATAA GTTCGTTTTT ATTATTTTTG

6241 TTGTAAATAA AAGGTATATA TATTATGTGT ATATAAGCGC GTCTATATGT ACTTACATTA

6301 TATACATATG CGCGCATACC ATATTTGTGG GATTTTGTTT TTTTCCTCAT AATTAATTTT

6361 TTAAAGAATA AGCTACACTT TTTCAGTTAA AATTTTATGT ACTACCATCA TTTTTTTTTA

6421 AATACTTCAA AAGAGAAATA AAAGCAAAAT AATATAAATC CTATAATTGT GTTGTTAGTT

6481 TGCCGTTTAC ATTTTCATAA ATATGTTTTT GAAATTTCAA TAAATATTGT GTACACATAT

6541 ATGTAGTATG TATGTATGCA TATGAGAATG AAGTATATTT GTATCATATG CTATGTTTTT

6601 TTATTTTTTT TTAAGTACAA TTTAATTTTT TTTTTCATTA TATTGTTACA ATAATTTTGG

6661 ATTTTTATGA AACTTTTAAA CGAAAAGTTA TGCATTTTTC TTTTATTTTT TTAACATCAA

6721 TTTTTATTAT ATATATATAA GATAAAATTC TTTACTAACA CAATAACATT ATTAAGCATA

6781 TAAAAATACC AAATTAAATT GAATTAAATT AAATTAAATT AAATTACTAG TTTATAATTT

6841 CCCAAAATGA AAATACTAAG GGTTTTATTT TTTTAATTAT ACGATTGGCA TATGGATACT

6901 TAGCATAGAG TGggcggccg cgcgggccc

//

> Py1042_PyNOT1-G::GFP

LOCUS pSL1042__final_ 6754 bp DNA circular 21-APR-2021

SOURCE

ORGANISM

COMMENT Lindner Lab - used to create Py1042 = Py17XNL PyNOT1-G::GFP

COMMENT

COMMENT ApEinfo:methylated:1

FEATURES Location/Qualifiers

exon 5731..6294

/vntifkey="61"

/locus_tag="hDHFR"

/label="hDHFR"

/ApEinfo_label="hDHFR"

/ApEinfo_fwdcolor="pink"

/ApEinfo_revcolor="pink"

/ApEinfo_graphicformat="arrow_data {{0 1 2 0 0 -1} {} 0}

width 5 offset 0"

misc_feature 1512..2190

/locus_tag="GFPmut2"

/label="GFPmut2"

/ApEinfo_label="GFPmut2"

/ApEinfo_fwdcolor="#8080c0"

/ApEinfo_revcolor="#ff8000"

/ApEinfo_graphicformat="arrow_data {{0 1 2 0 0 -1} {} 0}

width 5 offset 0"

misc_feature 2197..2644

/locus_tag="PbDHFR/TS\3'UTR(1)"

/label="PbDHFR/TS\3'UTR(1)"

/ApEinfo_label="PbDHFR/TS\3'UTR(1)"

/ApEinfo_fwdcolor="#8080c0"

/ApEinfo_revcolor="#ff8000"

/ApEinfo_graphicformat="arrow_data {{0 1 2 0 0 -1} {} 0}

width 5 offset 0"

misc_feature 748..1470

/locus_tag="PyNOT1-G ORF Homology Arm"

/label="PyNOT1-G ORF Homology Arm"

/ApEinfo_label="PyNOT1-G ORF Homology Arm"

/ApEinfo_fwdcolor="cyan"

/ApEinfo_revcolor="green"

/ApEinfo_graphicformat="arrow_data {{0 1 2 0 0 -1} {} 0}

width 5 offset 0"

rep_origin 4041..4723

/locus_tag="ColE1 origin"

/label="ColE1 origin"

/ApEinfo_label="ColE1 origin"

/ApEinfo_fwdcolor="pink"

/ApEinfo_revcolor="pink"

/ApEinfo_graphicformat="arrow_data {{0 1 2 0 0 -1} {} 0}

width 5 offset 0"

CDS 3284..3943

/locus_tag="AmpR"

/label="AmpR"

/ApEinfo_label="AmpR"

/ApEinfo_fwdcolor="pink"

/ApEinfo_revcolor="pink"

/ApEinfo_graphicformat="arrow_data {{0 1 2 0 0 -1} {} 0}

width 5 offset 0"

misc_feature join(5127..5436,5441..5725)

/locus_tag="PbEF1a 5'UTR"

/label="PbEF1a 5'UTR"

/ApEinfo_label="PbEF1a 5'UTR"

/ApEinfo_fwdcolor="#8080c0"

/ApEinfo_revcolor="#ff8000"

/ApEinfo_graphicformat="arrow_data {{0 1 2 0 0 -1} {} 0}

width 5 offset 0"

misc_feature 6302..6749

/locus_tag="PbDHFR-TS 3'UTR(1)"

/label="PbDHFR-TS 3'UTR(1)"

/ApEinfo_label="PbDHFR-TS 3'UTR(1)"

/ApEinfo_fwdcolor="#8080c0"

/ApEinfo_revcolor="#ff8000"

/ApEinfo_graphicformat="arrow_data {{0 1 2 0 0 -1} {} 0}

width 5 offset 0"

misc_feature 7..730

/locus_tag="PyNOT1-G 3' Homology Arm"

/label="PyNOT1-G 3' Homology Arm"

/ApEinfo_label="PyNOT1-G 3' Homology Arm"

/ApEinfo_fwdcolor="cyan"

/ApEinfo_revcolor="green"

/ApEinfo_graphicformat="arrow_data {{0 0.5 0 1 2 0 0 -1 0

-0.5} {} 0} width 5 offset 0"

ORIGIN

1 ggtaccCAAC TCATACAAAC TCTTCTTAGA ATGTAATATA TATGTACATG TATATATATT

61 ATTATTATTA TTATTTTATT TTTTTTTTTT TTTTTTTTTA CTTACACATG TATGTATGCA

121 CATACATATA TTACAATTTT ATACCATTTT AAAATTAGTC TTATTCATCA TCGAAGAAAA

181 ATTTATTATC CTTTGCATAC ATACATACAT ACATATATAT ATATATATAT ACATATATAT

241 ATACATGCAT TTATTTTACT ATTTTTTTAC AATTTATAAT TGTATTTTTA AAATAGCAGT

301 TTTGTAATTT TTTTCAAATT CATATTTTTT TTTTCCCATT TTTTTGTTAA ACTTTTAAAA

361 CACAACAATA GTATCGATAG TACTAGTAGT ATATAATTAT AAAATGCGTT TATTTATTTT

421 TTCTACATTT CTACAATTTT TCTACAATTT TCCTACAATT TTAAAATACC ACATATAAAC

481 CTCAATACAC ACATCCATTC CGTTTTAATA AAAAAAAAAA TATATATTTC CGAAATAACG

541 CTAGGAGGAA AATAAAATTC CCCTGATTTT TACTATCATT TTAATGTTTT CATTATTCTA

601 TATCTTTAAT AATATACATG AAATGTAATT TTTATGTGTG CAAAAATATT TCAAGTTTAT

661 TATTAAGCAA AAAATTTACA AAAATATATA CACATATGAG AAATATGAAT CCCGCTTTGT

721 CTATCCATAC ggcggccgcg cgggcccCTC TCAAATATTT CGAATTCCAT ACAAAATAAC

781 AAACAACCAA TTTTAGATGA AAATAAAGAT AAAATAAAAG AATTTTATAA TAATATAAAT

841 ATGAATCAAT TATTGCAAAA TTCTAACGAG AAAAATAATA TTAATAATAC TGATTTAATT

901 AGGCAAATTC TAAATTCAGA AAATGTTAAT TTAAAAAATT ATACTACAAC TAATGGGCCT

961 ACTATTACTC CTCATGTTAA ATTCACTAAC CAATATGATA CCAATTCACA CCCAAATGAC

1021 ATAACAAATA ATATAAAACA AAAACAAAAT GTTATTAATA ATTTACACAC ATATAATATA

1081 AATTCGAATA ATAACAATAG TAGCAATAAC AATAACAATC ACATTTTGAA TGCTTCGTTT

1141 TATAGAGATA TTGGAAATTT GAGTGGAATA AAAACAAATA ATAATTCACA AAACAATATC

1201 GAATTATCAC AAAATAATCT ATTCATTAAT AACCCCTACT ATTTTCAGGA TAAAAATATT

1261 ATGTTAGATT CACATTTACA AAATAATATT AATCAAATTA AAAGAAATCC AAGATTAAAT

1321 AACTCACAAA TACATGAAAA TAATGAAAAT GATTCAAATA ATATTCAAAA ACATTTTAAT

1381 TTCGAAATAA ATAATAAATT AACAGATCAA AATAATAGAG ATTTTTTCAA TTCAAATCAT

1441 TTTTATGACT CAAAACCTGA TTACTATAAC cctagtagta aaggagaaga acttttcact

1501 ggagttgtcc cAATTCTTGT TGAATTAGAT GGTGATGTTA ATGGGCACAA ATTTTCTGTC

1561 AGTGGAGAGG GTGAAGGTGA TGCAACATAC GGAAAACTTA CCCTTAAATT TATTTGCACT

1621 ACTGGAAAAC TACCTGTTCC ATGGCCAACA CTTGTCACTA CTTTCGCGTA TGGTCTTCAA

1681 TGCTTTGCGA GATACCCAGA TCATATGAAA CAGCATGACT TTTTCAAGAG TGCCATGCCC

1741 GAAGGTTATG TACAGGAAAG AACTATATTT TTCAAAGATG ACGGGAACTA CAAGACACGT

1801 GCTGAAGTCA AGTTTGAAGG TGATACCCTT GTTAATAGAA TCGAGTTAAA AGGTATTGAT

1861 TTTAAAGAAG ATGGAAACAT TCTTGGACAC AAATTGGAAT ACAACTATAA CTCACACAAT

1921 GTATACATCA TGGCAGACAA ACAAAAGAAT GGAATCAAAG TTAACTTCAA AATTAGACAC

1981 AACATTGAAG ATGGAAGCGT TCAACTAGCA GACCATTATC AACAAAATAC TCCAATTGGC

2041 GATGGCCCTG TCCTTTTACC AGACAACCAT TACCTGTCCA CACAATCTGC CCTTTCGAAA

2101 GATCCCAACG AAAAGAGAGA CCACATGGTC CTTCTTGAGT TTGTAACAGC TGCTGGGATT

2161 ACACATGGCA TGGATGAACT ATACAAATAA ggatccGTTT TTCTTACTTA TATATTTATA

2221 CCAATTGATT GTATTTATAA CTGTAAAAAT GTGTATGTTG TGTGCATATT TTTTTTTGTG

2281 CATGCACATG CATGTAAATA GCTAAAATTA TGAACATTTT ATTTTTTGTT CAGAAAAAAA

2341 AAACTTTACA CACATAAAAT GGCTAGTATG AATAGCCATA TTTTATATAA ATTAAATCCT

2401 ATGAATTTAT GACCATATTA AAAATTTAGA TATTTATGGA ACATAATATG TTTGAAACAA

2461 TAAGACAAAA TTATTATTAT TATTATTATT TTTACTGTTA TAATTATGTT GTCTCTTCAA

2521 TGATTCATAA ATAGTTGGAC TTGATTTTTA AAATGTTTAT AATATGATTA GCATAGTTAA

2581 ATAAAAAAAG TTGAAAAATT AAAAAAAAAC ATATAAACAC AAATGATGTT TTTTCCTTCA

2641 ATTTcggcgc ctgatgcggt attttctcct tacgcatctg tgcggtattt cacaccgcat

2701 atggtgcact ctcagtacaa tctgctctga tgccgcatag ttaagccagc cccgacaccc

2761 gccaacaccc gctgacgcgc cctgacgggc ttgtctgctc ccggcatccg cttacagaca

2821 agctgtgacc gtctccggga gctgcatgtg tcagaggttt tcaccgtcat caccgaaacg

2881 cgcgagacga aagggcctcg tgatacgcct atttttatag gttaatgtca tgataataat

2941 ggtttcttag acgtcaggtg gcacttttcg gggaaatgtg cgcggaaccc ctatttgttt

3001 atttttctaa atacattcaa atatgtatcc gctcatgaga caataaccct gataaatgct

3061 tcaataatat tgaaaaagga agagtatgag tattcaacat ttccgtgtcg cccttattcc

3121 cttttttgcg gcattttgcc ttcctgtttt tgctcaccca gaaacgctgg tgaaagtaaa

3181 agatgctgaa gatcagttgg gtgcacgagt gggttacatc gaactggatc tcaacagcgg

3241 taagatcctt gagagttttc gccccgaaga acgttttcca atgatgagca cttttaaagt

3301 tctgctatgt ggcgcggtat tatcccgtat tgacgccggg caagagcaac tcggtcgccg

3361 catacactat tctcagaatg acttggttga gtactcacca gtcacagaaa agcatcttac

3421 ggatggcatg acagtaagag aattatgcag tgctgccata accatgagtg ataacactgc

3481 ggccaactta cttctgacaa cgatcggagg accgaaggag ctaaccgctt ttttgcacaa

3541 catgggggat catgtaactc gccttgatcg ttgggaaccg gagctgaatg aagccatacc

3601 aaacgacgag cgtgacacca cgatgcctgt agcaatggca acaacgttgc gcaaactatt

3661 aactggcgaa ctacttactc tagcttcccg gcaacaatta atagactgga tggaggcgga

3721 taaagttgca ggaccacttc tgcgctcggc ccttccggct ggctggttta ttgctgataa

3781 atctggagcc ggtgagcgtg ggtctcgcgg tatcattgca gcactggggc cagatggtaa

3841 gccctcccgt atcgtagtta tctacacgac ggggagtcag gcaactatgg atgaacgaaa

3901 tagacagatc gctgagatag gtgcctcact gattaagcat tggtaactgt cagaccaagt

3961 ttactcatat atactttaga ttgatttaaa acttcatttt taatttaaaa ggatctaggt

4021 gaagatcctt tttgataatc tcatgaccaa aatcccttaa cgtgagtttt cgttccactg

4081 agcgtcagac cccgtagaaa agatcaaagg atcttcttga gatccttttt ttctgcgcgt

4141 aatctgctgc ttgcaaacaa aaaaaccacc gctaccagcg gtggtttgtt tgccggatca

4201 agagctacca actctttttc cgaaggtaac tggcttcagc agagcgcaga taccaaatac

4261 tgtccttcta gtgtagccgt agttaggcca ccacttcaag aactctgtag caccgcctac

4321 atacctcgct ctgctaatcc tgttaccagt ggctgctgcc agtggcgata agtcgtgtct

4381 taccgggttg gactcaagac gatagttacc ggataaggcg cagcggtcgg gctgaacggg

4441 gggttcgtgc acacagccca gcttggagcg aacgacctac accgaactga gatacctaca

4501 gcgtgagcat tgagaaagcg ccacgcttcc cgaagggaga aaggcggaca ggtatccggt

4561 aagcggcagg gtcggaacag gagagcgcac gagggagctt ccagggggaa acgcctggta

4621 tctttatagt cctgtcgggt ttcgccacct ctgacttgag cgtcgatttt tgtgatgctc

4681 gtcagggggg cggagcctat ggaaaaacgc cagcaacgcg gcctttttac ggttcctggc

4741 cttttgctgg ccttttgctc acatgttctt tcctgcgtta tcccctgatt ctgtggataa

4801 ccgtattacc gcctttgagt gagctgatac cgctcgccgc agccgaacga ccgagcgcag

4861 cgagtcagtg agcgaggaag cggaagagcg cccaatacgc aaaccgcctc tccccgcgcg

4921 ttggccgatt cattaatgca gctggcacga caggtttccc gactggaaag cgggcagtga

4981 gcgcaacgca attaatgtga gttagctcac tcattaggca ccccaggctt tacactttat

5041 gcttccggct cgtatgttgt gtggaattgt gagcggataa caatttcaca caggaaacag

5101 ctatgaccat gattacgcca agcttgataa ttcctgcagc ccagcttaat tcttttcgag

5161 ctctttatgc ttaagtttac aatttaatat tcatacttta agtatttttt gtagtatcct

5221 agatattgtg ctttaaatgc tcacccctca aagcaccagt aatattttca tccactgaaa

5281 taccattaaa ttttcaaaaa aatactatgc atataatgtt atacatataa acataaaacg

5341 ccatgtaaat caaaaaatat ataaaaatat gtataaaaat aaatatgcac taaatataag

5401 ctaattatgc ataaaaatta aagtgccctt tattaactag ctagtcgtaa ttatttatat

5461 ttctatgtta taaaaaaatc ctcatataat aatataatta atatatgtaa tgtttttttt

5521 attttataat tttaatataa aataatatgt aaattaattc aaaaaataaa tataattgtt

5581 gtgaaacaaa aaacgtaatt ttttcatttg ccttcaaaat ttaaatttat tttaatattt

5641 cctaaaatat atatactttg tgtataaata tataaaaata tatatttgct tataaataaa

5701 taaaaaattt tataaaacat agggggatcc atggttggtt cgctaaactg catcgtcgct

5761 gtgtcccaga acatgggcat cggcaagaac ggggacctgc cctggccacc gctcaggaac

5821 gaatttagat atttccagag aatgaccaca acctcttcag tagaaggtaa acagaatctg

5881 gtgattatgg gtaagaagac ctggttctcc attcctgaga agaatcgacc tttaaagggt

5941 agaattaatt tagttctcag cagagaactc aaggaacctc cacaaggagc tcattttctt

6001 tccagaagtc tagatgatgc cttaaaactt actgaacaac cagaattagc aaataaagta

6061 gacatggtct ggatagttgg tggcagttct gtttataagg aagccatgaa tcacccaggc

6121 catcttaaac tatttgtgac aaggatcatg caagactttg aaagtgacac gttttttcca

6181 gaaattgatt tggagaaata taaacttctg ccagaatacc caggtgttct ctctgatgtc

6241 caggaggaga aaggcattaa gtacaaattt gaagtatatg agaagaatga ttaaggatcc

6301 cgtttttctt acttatatat ttataccaat tgattgtatt tataactgta aaaatgtgta

6361 tgttgtgtgc atattttttt ttgtgcatgc acatgcatgt aaatagctaa aattatgaac

6421 attttatttt ttgttcagaa aaaaaaaact ttacacacat aaaatggcta gtatgaatag

6481 ccatatttta tataaattaa atcctatgaa tttatgacca tattaaaaat ttagatattt

6541 atggaacata atatgtttga aacaataaga caaaattatt attattatta ttatttttac

6601 tgttataatt atgttgtctc ttcaatgatt cataaatagt tggacttgat ttttaaaatg

6661 tttataatat gattagcata gttaaataaa aaaagttgaa aaattaaaaa aaaacatata

6721 aacacaaatg atgttttttc cttcaatttc gatg

//

> Py1023_PyNOT1::GFP

LOCUS pSL1023__final_ 6767 bp DNA circular 21-APR-2021

SOURCE

ORGANISM

COMMENT Lindner Lab - used to create Py1023 = Py17XNL PyNOT1::GFP

COMMENT

COMMENT ApEinfo:methylated:1

FEATURES Location/Qualifiers

exon 5744..6307

/vntifkey="61"

/locus_tag="hDHFR"

/label="hDHFR"

/ApEinfo_label="hDHFR"

/ApEinfo_fwdcolor="pink"

/ApEinfo_revcolor="pink"

/ApEinfo_graphicformat="arrow_data {{0 1 2 0 0 -1} {} 0}

width 5 offset 0"

misc_feature 1525..2203

/locus_tag="GFPmut2"

/label="GFPmut2"

/ApEinfo_label="GFPmut2"

/ApEinfo_fwdcolor="#8080c0"

/ApEinfo_revcolor="#ff8000"

/ApEinfo_graphicformat="arrow_data {{0 1 2 0 0 -1} {} 0}

width 5 offset 0"

misc_feature 2210..2657

/locus_tag="PbDHFR/TS\3'UTR"

/label="PbDHFR/TS\3'UTR"

/ApEinfo_label="PbDHFR/TS\3'UTR"

/ApEinfo_fwdcolor="#8080c0"

/ApEinfo_revcolor="#ff8000"

/ApEinfo_graphicformat="arrow_data {{0 1 2 0 0 -1} {} 0}

width 5 offset 0"

misc_feature 744..1478

/locus_tag="PyNOT1 ORF Homology Arm"

/label="PyNOT1 ORF Homology Arm"

/ApEinfo_label="PyNOT1 ORF Homology Arm"

/ApEinfo_fwdcolor="cyan"

/ApEinfo_revcolor="green"

/ApEinfo_graphicformat="arrow_data {{0 1 2 0 0 -1} {} 0}

width 5 offset 0"

rep_origin 4054..4736

/locus_tag="ColE1 origin"

/label="ColE1 origin"

/ApEinfo_label="ColE1 origin"

/ApEinfo_fwdcolor="pink"

/ApEinfo_revcolor="pink"

/ApEinfo_graphicformat="arrow_data {{0 1 2 0 0 -1} {} 0}

width 5 offset 0"

CDS 3297..3956

/locus_tag="AmpR"

/label="AmpR"

/ApEinfo_label="AmpR"

/ApEinfo_fwdcolor="pink"

/ApEinfo_revcolor="pink"

/ApEinfo_graphicformat="arrow_data {{0 1 2 0 0 -1} {} 0}

width 5 offset 0"

misc_feature join(5140..5449,5454..5738)

/locus_tag="PbEF1a 5'UTR"

/label="PbEF1a 5'UTR"

/ApEinfo_label="PbEF1a 5'UTR"

/ApEinfo_fwdcolor="#8080c0"

/ApEinfo_revcolor="#ff8000"

/ApEinfo_graphicformat="arrow_data {{0 1 2 0 0 -1} {} 0}

width 5 offset 0"

misc_feature 6315..6762

/locus_tag="PbDHFR-TS 3'UTR"

/label="PbDHFR-TS 3'UTR"

/ApEinfo_label="PbDHFR-TS 3'UTR"

/ApEinfo_fwdcolor="#8080c0"

/ApEinfo_revcolor="#ff8000"

/ApEinfo_graphicformat="arrow_data {{0 1 2 0 0 -1} {} 0}

width 5 offset 0"

misc_feature 7..726

/locus_tag="PyNOT1 3' Homology Arm"

/label="PyNOT1 3' Homology Arm"

/ApEinfo_label="PyNOT1 3' Homology Arm"

/ApEinfo_fwdcolor="cyan"

/ApEinfo_revcolor="green"

/ApEinfo_graphicformat="arrow_data {{0 0.5 0 1 2 0 0 -1 0

-0.5} {} 0} width 5 offset 0"

ORIGIN

1 ggtaccCATA TTGTTATTTT TAGAATATTT ATAAGTTCGT TTTTATTATT TTTGTTGTAA

61 ATAAAAGGTA TATATATTAT GTGTATATAA GCGCGTCTAT ATGTACTTAC ATTATATACA

121 TATGCGCGCA TACCATATTT GTGGGATTTT GTTTTTTTCC TCATAATTAA TTTTTTAAAG

181 AATAAGCTAC ACTTTTTCAG TTAAAATTTT ATGTACTACC ATCATTTTTT TTTAAATACT

241 TCAAAAGAGA AATAAAAGCA AAATAATATA AATCCTATAA TTGTGTTGTT AGTTTGCCGT

301 TTACATTTTC ATAAATATGT TTTTGAAATT TCAATAAATA TTGTGTACAC ATATATGTAG

361 TATGTATGTA TGCATATGAG AATGAAGTAT ATTTGTATCA TATGCTATGT TTTTTTATTT

421 TTTTTTAAGT ACAATTTAAT TTTTTTTTTC ATTATATTGT TACAATAATT TTGGATTTTT

481 ATGAAACTTT TAAACGAAAA GTTATGCATT TTTCTTTTAT TTTTTTAACA TCAATTTTTA

541 TTATATATAT ATAAGATAAA ATTCTTTACT AACACAATAA CATTATTAAG CATATAAAAA

601 TACCAAATTA AATTGAATTA AATTAAATTA AATTAAATTA CTAGTTTATA ATTTCCCAAA

661 ATGAAAATAC TAAGGGTTTT ATTTTTTTAA TTATACGATT GGCATATGGA TACTTAGCAT

721 AGAGTGggcg gccgcgcggg cccCAAGGAG AAATTTTAAA ACGAAAAGAC ATAAAAGAAG

781 GAGATGAAAA ACAACCAAAT CAAGAAAATC AAAGAAATGG AGAACAAAAT AATAATTTGC

841 CTAACAAATA TGTAGATCTT GTGCAAGAAA ATAATATAGA AAATGAAATT TCATCATTTT

901 CAAATTGTGA TAAAACAAAT ACTAAAGTGT TAAAACAAAA TAGTGTAAAT AATAAAGAAG

961 ATAATGATGG CACAATAGAA ATTACTATTA TCAAAAAAAA TCTTGCTTAT ACATTATTTT

1021 TGTTTTTATT AAAAGAATTA GATATGGAAG GAAGATACTT ATTATTATTG AATATAGTTA

1081 ATCATATTAG ATATCCTAAT TCGCATACCC ATTATTTTTC ATGTCTCATT TTGTTTTTAT

1141 TTTCATATTC AAATGATATC GTTATTAAAG AACAAATAAT TCGGGTTTTG CTGGAAAGAA

1201 TTTTAGCTCA TAGGCCACAT CCATGGGGAT TATTAATTAC ATTTATTGAA TTAATCAAAA

1261 ATAAAAAGTA AGAGAATATT TATTTTTTAC ACAAACAAAA AGAGGATATA CATAAATAAG

1321 CATGTATATA ATGTATATAC TAAAATACAC ATTATTTACA TATTAATATA ATACCTTTTT

1381 TTTATAGATT CAAAATTTGG GAATATCCAT TTGTACATGC CACATCGGAG ATTAAAAAAA

1441 TATTCAAGTC AGTTTTTCAG ACATGCTTAG GAAATGTTcc taggctagta gtaaaggaga

1501 agaacttttc actggagttg tcccAATTCT TGTTGAATTA GATGGTGATG TTAATGGGCA

1561 CAAATTTTCT GTCAGTGGAG AGGGTGAAGG TGATGCAACA TACGGAAAAC TTACCCTTAA

1621 ATTTATTTGC ACTACTGGAA AACTACCTGT TCCATGGCCA ACACTTGTCA CTACTTTCGC

1681 GTATGGTCTT CAATGCTTTG CGAGATACCC AGATCATATG AAACAGCATG ACTTTTTCAA

1741 GAGTGCCATG CCCGAAGGTT ATGTACAGGA AAGAACTATA TTTTTCAAAG ATGACGGGAA

1801 CTACAAGACA CGTGCTGAAG TCAAGTTTGA AGGTGATACC CTTGTTAATA GAATCGAGTT

1861 AAAAGGTATT GATTTTAAAG AAGATGGAAA CATTCTTGGA CACAAATTGG AATACAACTA

1921 TAACTCACAC AATGTATACA TCATGGCAGA CAAACAAAAG AATGGAATCA AAGTTAACTT

1981 CAAAATTAGA CACAACATTG AAGATGGAAG CGTTCAACTA GCAGACCATT ATCAACAAAA

2041 TACTCCAATT GGCGATGGCC CTGTCCTTTT ACCAGACAAC CATTACCTGT CCACACAATC

2101 TGCCCTTTCG AAAGATCCCA ACGAAAAGAG AGACCACATG GTCCTTCTTG AGTTTGTAAC

2161 AGCTGCTGGG ATTACACATG GCATGGATGA ACTATACAAA TAAggatccG TTTTTCTTAC

2221 TTATATATTT ATACCAATTG ATTGTATTTA TAACTGTAAA AATGTGTATG TTGTGTGCAT

2281 ATTTTTTTTT GTGCATGCAC ATGCATGTAA ATAGCTAAAA TTATGAACAT TTTATTTTTT

2341 GTTCAGAAAA AAAAAACTTT ACACACATAA AATGGCTAGT ATGAATAGCC ATATTTTATA

2401 TAAATTAAAT CCTATGAATT TATGACCATA TTAAAAATTT AGATATTTAT GGAACATAAT

2461 ATGTTTGAAA CAATAAGACA AAATTATTAT TATTATTATT ATTTTTACTG TTATAATTAT

2521 GTTGTCTCTT CAATGATTCA TAAATAGTTG GACTTGATTT TTAAAATGTT TATAATATGA

2581 TTAGCATAGT TAAATAAAAA AAGTTGAAAA ATTAAAAAAA AACATATAAA CACAAATGAT

2641 GTTTTTTCCT TCAATTTcgg cgcctgatgc ggtattttct ccttacgcat ctgtgcggta

2701 tttcacaccg catatggtgc actctcagta caatctgctc tgatgccgca tagttaagcc

2761 agccccgaca cccgccaaca cccgctgacg cgccctgacg ggcttgtctg ctcccggcat

2821 ccgcttacag acaagctgtg accgtctccg ggagctgcat gtgtcagagg ttttcaccgt

2881 catcaccgaa acgcgcgaga cgaaagggcc tcgtgatacg cctattttta taggttaatg

2941 tcatgataat aatggtttct tagacgtcag gtggcacttt tcggggaaat gtgcgcggaa

3001 cccctatttg tttatttttc taaatacatt caaatatgta tccgctcatg agacaataac

3061 cctgataaat gcttcaataa tattgaaaaa ggaagagtat gagtattcaa catttccgtg

3121 tcgcccttat tccctttttt gcggcatttt gccttcctgt ttttgctcac ccagaaacgc

3181 tggtgaaagt aaaagatgct gaagatcagt tgggtgcacg agtgggttac atcgaactgg

3241 atctcaacag cggtaagatc cttgagagtt ttcgccccga agaacgtttt ccaatgatga

3301 gcacttttaa agttctgcta tgtggcgcgg tattatcccg tattgacgcc gggcaagagc

3361 aactcggtcg ccgcatacac tattctcaga atgacttggt tgagtactca ccagtcacag

3421 aaaagcatct tacggatggc atgacagtaa gagaattatg cagtgctgcc ataaccatga

3481 gtgataacac tgcggccaac ttacttctga caacgatcgg aggaccgaag gagctaaccg

3541 cttttttgca caacatgggg gatcatgtaa ctcgccttga tcgttgggaa ccggagctga

3601 atgaagccat accaaacgac gagcgtgaca ccacgatgcc tgtagcaatg gcaacaacgt

3661 tgcgcaaact attaactggc gaactactta ctctagcttc ccggcaacaa ttaatagact

3721 ggatggaggc ggataaagtt gcaggaccac ttctgcgctc ggcccttccg gctggctggt

3781 ttattgctga taaatctgga gccggtgagc gtgggtctcg cggtatcatt gcagcactgg

3841 ggccagatgg taagccctcc cgtatcgtag ttatctacac gacggggagt caggcaacta

3901 tggatgaacg aaatagacag atcgctgaga taggtgcctc actgattaag cattggtaac

3961 tgtcagacca agtttactca tatatacttt agattgattt aaaacttcat ttttaattta

4021 aaaggatcta ggtgaagatc ctttttgata atctcatgac caaaatccct taacgtgagt

4081 tttcgttcca ctgagcgtca gaccccgtag aaaagatcaa aggatcttct tgagatcctt

4141 tttttctgcg cgtaatctgc tgcttgcaaa caaaaaaacc accgctacca gcggtggttt

4201 gtttgccgga tcaagagcta ccaactcttt ttccgaaggt aactggcttc agcagagcgc

4261 agataccaaa tactgtcctt ctagtgtagc cgtagttagg ccaccacttc aagaactctg

4321 tagcaccgcc tacatacctc gctctgctaa tcctgttacc agtggctgct gccagtggcg

4381 ataagtcgtg tcttaccggg ttggactcaa gacgatagtt accggataag gcgcagcggt

4441 cgggctgaac ggggggttcg tgcacacagc ccagcttgga gcgaacgacc tacaccgaac

4501 tgagatacct acagcgtgag cattgagaaa gcgccacgct tcccgaaggg agaaaggcgg

4561 acaggtatcc ggtaagcggc agggtcggaa caggagagcg cacgagggag cttccagggg

4621 gaaacgcctg gtatctttat agtcctgtcg ggtttcgcca cctctgactt gagcgtcgat

4681 ttttgtgatg ctcgtcaggg gggcggagcc tatggaaaaa cgccagcaac gcggcctttt

4741 tacggttcct ggccttttgc tggccttttg ctcacatgtt ctttcctgcg ttatcccctg

4801 attctgtgga taaccgtatt accgcctttg agtgagctga taccgctcgc cgcagccgaa

4861 cgaccgagcg cagcgagtca gtgagcgagg aagcggaaga gcgcccaata cgcaaaccgc

4921 ctctccccgc gcgttggccg attcattaat gcagctggca cgacaggttt cccgactgga

4981 aagcgggcag tgagcgcaac gcaattaatg tgagttagct cactcattag gcaccccagg

5041 ctttacactt tatgcttccg gctcgtatgt tgtgtggaat tgtgagcgga taacaatttc

5101 acacaggaaa cagctatgac catgattacg ccaagcttga taattcctgc agcccagctt

5161 aattcttttc gagctcttta tgcttaagtt tacaatttaa tattcatact ttaagtattt

5221 tttgtagtat cctagatatt gtgctttaaa tgctcacccc tcaaagcacc agtaatattt

5281 tcatccactg aaataccatt aaattttcaa aaaaatacta tgcatataat gttatacata

5341 taaacataaa acgccatgta aatcaaaaaa tatataaaaa tatgtataaa aataaatatg

5401 cactaaatat aagctaatta tgcataaaaa ttaaagtgcc ctttattaac tagctagtcg

5461 taattattta tatttctatg ttataaaaaa atcctcatat aataatataa ttaatatatg

5521 taatgttttt tttattttat aattttaata taaaataata tgtaaattaa ttcaaaaaat

5581 aaatataatt gttgtgaaac aaaaaacgta attttttcat ttgccttcaa aatttaaatt

5641 tattttaata tttcctaaaa tatatatact ttgtgtataa atatataaaa atatatattt

5701 gcttataaat aaataaaaaa ttttataaaa cataggggga tccatggttg gttcgctaaa

5761 ctgcatcgtc gctgtgtccc agaacatggg catcggcaag aacggggacc tgccctggcc

5821 accgctcagg aacgaattta gatatttcca gagaatgacc acaacctctt cagtagaagg

5881 taaacagaat ctggtgatta tgggtaagaa gacctggttc tccattcctg agaagaatcg

5941 acctttaaag ggtagaatta atttagttct cagcagagaa ctcaaggaac ctccacaagg

6001 agctcatttt ctttccagaa gtctagatga tgccttaaaa cttactgaac aaccagaatt

6061 agcaaataaa gtagacatgg tctggatagt tggtggcagt tctgtttata aggaagccat

6121 gaatcaccca ggccatctta aactatttgt gacaaggatc atgcaagact ttgaaagtga

6181 cacgtttttt ccagaaattg atttggagaa atataaactt ctgccagaat acccaggtgt

6241 tctctctgat gtccaggagg agaaaggcat taagtacaaa tttgaagtat atgagaagaa

6301 tgattaagga tcccgttttt cttacttata tatttatacc aattgattgt atttataact

6361 gtaaaaatgt gtatgttgtg tgcatatttt tttttgtgca tgcacatgca tgtaaatagc

6421 taaaattatg aacattttat tttttgttca gaaaaaaaaa actttacaca cataaaatgg

6481 ctagtatgaa tagccatatt ttatataaat taaatcctat gaatttatga ccatattaaa

6541 aatttagata tttatggaac ataatatgtt tgaaacaata agacaaaatt attattatta

6601 ttattatttt tactgttata attatgttgt ctcttcaatg attcataaat agttggactt

6661 gatttttaaa atgtttataa tatgattagc atagttaaat aaaaaaagtt gaaaaattaa

6721 aaaaaaacat ataaacacaa atgatgtttt ttccttcaat ttcgatg

//

>Py1125_PyNOT1-G_TTPbd::GFP_Overexpression

LOCUS pSL1125__final_ 8361 bp DNA circular 21-APR-2021

SOURCE

ORGANISM

COMMENT Lindner Lab - used to create Py1125 = Py17XNL Overexpression of

PyNOT1-G's TTP-binding domain fused to GFP, expressed from p230p

locus

COMMENT ApEinfo:methylated:1

FEATURES Location/Qualifiers

exon 7338..7901

/vntifkey="61"

/locus_tag="hDHFR"

/label="hDHFR"

/ApEinfo_label="hDHFR"

/ApEinfo_fwdcolor="pink"

/ApEinfo_revcolor="pink"

/ApEinfo_graphicformat="arrow_data {{0 1 2 0 0 -1} {} 0}

width 5 offset 0"

misc_feature 2481..3077

/locus_tag="PyNOT1-G AA1-199"

/label="PyNOT1-G AA1-199"

/ApEinfo_label="PyNOT1-G AA1-199"

/ApEinfo_fwdcolor="cyan"

/ApEinfo_revcolor="green"

/ApEinfo_graphicformat="arrow_data {{0 1 2 0 0 -1} {} 0}

width 5 offset 0"

misc_feature 1898..2471

/locus_tag="PbEF1a promoter"

/label="PbEF1a promoter"

/ApEinfo_label="PbEF1a promoter"

/ApEinfo_fwdcolor="cyan"

/ApEinfo_revcolor="green"

/ApEinfo_graphicformat="arrow_data {{0 1 2 0 0 -1} {} 0}

width 5 offset 0"

misc_feature 3119..3797

/locus_tag="GFPmut2"

/label="GFPmut2"

/ApEinfo_label="GFPmut2"

/ApEinfo_fwdcolor="cyan"

/ApEinfo_revcolor="green"

/ApEinfo_graphicformat="arrow_data {{0 1 2 0 0 -1} {} 0}

width 5 offset 0"

misc_feature 3804..4251

/locus_tag="PbDHFR/TS\3'UTR"

/label="PbDHFR/TS\3'UTR"

/ApEinfo_label="PbDHFR/TS\3'UTR"

/ApEinfo_fwdcolor="#ff0000"

/ApEinfo_revcolor="green"

/ApEinfo_graphicformat="arrow_data {{0 1 2 0 0 -1} {} 0}

width 5 offset 0"

rep_origin 5648..6330

/locus_tag="ColE1 origin"

/label="ColE1 origin"

/ApEinfo_label="ColE1 origin"

/ApEinfo_fwdcolor="gray50"

/ApEinfo_revcolor="gray50"

/ApEinfo_graphicformat="arrow_data {{0 1 2 0 0 -1} {} 0}

width 5 offset 0"

CDS 4891..5550

/locus_tag="AmpR"

/label="AmpR"

/ApEinfo_label="AmpR"

/ApEinfo_fwdcolor="yellow"

/ApEinfo_revcolor="yellow"

/ApEinfo_graphicformat="arrow_data {{0 1 2 0 0 -1} {} 0}

width 5 offset 0"

misc_feature join(6734..7043,7048..7332)

/locus_tag="PbEF1a 5'UTR"

/label="PbEF1a 5'UTR"

/ApEinfo_label="PbEF1a 5'UTR"

/ApEinfo_fwdcolor="#804040"

/ApEinfo_revcolor="#804040"

/ApEinfo_graphicformat="arrow_data {{0 1 2 0 0 -1} {} 0}

width 5 offset 0"

misc_feature 7909..8356

/locus_tag="PbDHFR-TS 3'UTR"

/label="PbDHFR-TS 3'UTR"

/ApEinfo_label="PbDHFR-TS 3'UTR"

/ApEinfo_fwdcolor="cyan"

/ApEinfo_revcolor="green"

/ApEinfo_graphicformat="arrow_data {{0 1 2 0 0 -1} {} 0}

width 5 offset 0"

misc_feature 7..918

/locus_tag="py p230p Homology Arm 1"

/label="py p230p Homology Arm 1"

/ApEinfo_label="py p230p Homology Arm 1"

/ApEinfo_fwdcolor="cyan"

/ApEinfo_revcolor="green"

/ApEinfo_graphicformat="arrow_data {{0 0.5 0 1 2 0 0 -1 0

-0.5} {} 0} width 5 offset 0"

misc_feature 925..1873

/locus_tag="py p230p Homology Arm 2"

/label="py p230p Homology Arm 2"

/ApEinfo_label="py p230p Homology Arm 2"

/ApEinfo_fwdcolor="cyan"

/ApEinfo_revcolor="green"

/ApEinfo_graphicformat="arrow_data {{0 0.5 0 1 2 0 0 -1 0

-0.5} {} 0} width 5 offset 0"

ORIGIN

1 ggtaccAGCT TTGTGTTTTA TTTGGATGTG CAACAATAGA TTTATGATTA ACCAATGGTA

61 AATCATTATA ATCATATATT TCATCTTCAT TTACATATTT TACTAAAACA GTTTTTACAA

121 TTTGTTCTGT TTCTTTATCT ACACAATGAC ATTCAAATTG ATATGATTTT GTTAATGTAT

181 AATTAAAACC AGCTAATGCA TATTCTGGAT TTTCAAATGA ATAGTTAATA ATATTTTTAG

241 TATTAATTTT ATAGGAATCA TGTAGATAGC CTTTTTCATA TACTTGAGTA AAACATTTTT

301 CTGGCATTTT TTTTGTATTT TCGGGGCATC TAAAACCAAA ACTTGTATTT GATTTGGAAA

361 TTATTATACA TAGATCTCCT TCACTATAAG CATTTAAATA ATTATATAAT TCACCTTTTC

421 TAAAATCACA AAATGAAACT GGTCTATCTT TTAAATCTGT TCGAACATGT AATAAAATAA

481 TTGCTCTTCT ATAATCAGTC CCACCATTTG TTACACCACA TTCTATGGTT AGTGGATATG

541 ATCCATGATT TATAAATGAG GTCATAAAAA ATTTAAAACC TTTAAATTGT TCATTTATTT

601 CTGGTATATC TATATCTTTT GTATTAAAAA ATAAATCTGT AAATTTATTT ATAGGAAATA

661 CATTACTTAA ATATTTTATG GTATTAATAG GTTCTTTACC ATATACGATT CTATTATCTG

721 TTATTTGCTC ACAATTATAC ATGAATAAAC TAAATTCATT TATTGTTTTT TCACATATTT

781 GTATTTCACT TAATTTGCTT AAATTATAAT CAAAATTACA CATCTGATAA GGTATATCGT

841 TAAAATATTT AATCGTTTTA CTATCATCAT CAACTATTTT TAAAATGTGA TCAGATGTTG

901 CCATTCCATC ACCAAATGgg gcccTATGGA ACTACATCTA TATAAGAGAT TTTTTTATTT

961 TTATATATTG GTTTTGAATC TAGATATTCA TAATTTTTAA TATATATTGT TCCTATATTT

1021 TTGGTTTTAT AATCTTCACA TGTACAATAA AAATCAAATT CTTTTTTTGT AATTTTTGAA

1081 ATCATTAAAA ATGTTGATGA ATTATTTATA AAATAATCTG CATCTATAAC TTTTATTTCA

1141 TTAAATATAT TTTCGATATT TTCAGTTATT TGTTCATTCA TGTTACTTTT TATATATACC

1201 TTTCTAAAAC AATTTTTTGG ATTAATTTTT CCAGTTGGAC AATTTAAGCT AACTACTATA

1261 TTTTCTTTTA ATTCGATTTC ACAAAATTCA TTTTGTTTTT CTAAATCATA AACATTGGAA

1321 TAGTAAAATT TTTTTTTAGA ATTACCTGAA AAATCACATC CATATAATTT ATTTTTTGTT

1381 GTATTTTGAG AAACCCTTAT CACTGTAATA CCTTTTTGAC CTTTCATATT ATTAATTATT

1441 GTTTTAGTAT TATCACAATA TATTTTCAAT ATTAATACTT TTTCTACAAT TAATGGAGTA

1501 ACAAAAGTGA AATTTCCTTT CTCTTTGAAC ATTTTATTTT TATCTAATAT GATAACACCT

1561 GGTAATATAT CATCTAATTT TTCTTTTTTG TCTAGATACA TTACTTCATC ATTTTCTGGT

1621 ATGTATTCTA TATTTTTATA TTTTTCTAAT TCTTCTGGTT CAACATTTGC ATTTTCTAAA

1681 TAAAAATTAT TTTTTTGACT ACTACGTTGT GATGTTTTCT TATTATTTAT TATGATATTA

1741 CTATCAAAAA TGTCATCATC ACTTTTTACA ATTTTGGGAC ATTTAACTTG AACCATATTC

1801 AGATTCTGTT CTAGATGTAT ATAACATGAA TGAACTGTAT CTACTTCATT AACGGGTTCA

1861 AGAGAATTCG GTAgccgcgg tggcggccgc TctcgagAGC TTAATTCTTT TCGAGCTCTT

1921 TATGCTTAAG TTTACAATTT AATATTCATA CTTTAAGTAT TTTTTGTAGT ATCCTAGATA

1981 TTGTGCTTTA AATGCTCACC CCTCAAAGCA CCAGTAATAT TTTCATCCAC TGAAATACCA

2041 TTAAATTTTC AAAAAAATAC TATGCATATA ATGTTATACA TATAAACATA AAACGCCATG

2101 TAAATCAAAA AATATATAAA AATATGTATA AAAATAAATA TGCACTAAAT ATAAGCTAAT

2161 TATGCATAAA AATTAAAGTG CCCTTTATTA ACTAGctagT CGTAATTATT TATATTTCTA

2221 TGTTATAAAA AAATCCTCAT ATAATAATAT AATTAATATA TGTAATGTTT TTTTTATTTT

2281 ATAATTTTAA TATAAAATAA TATGTAAATT AATTCAAAAA ATAAATATAA TTGTTGTGAA

2341 ACAAAAAACG TAATTTTTTC ATTTGCCTTC AAAATTTAAA TTTATTTTAA TATTTCCTAA

2401 AATATATATA CTTTGTGTAT AAATATATAA AAATATATAT TTGCTTATAA ATAAATAAAA

2461 ATTTTATAAA Aatgactagg ATGAATAACA ATTTTAACAT TAATCTTCAG ATCGAGGATG

2521 GAATCACCAA TAAATATGAA GCCGAAGTAA ATGGCTATTT TGCTAAATTG TACACAGGGG

2581 AAATAACAGT CAACACAATG ATAGATATCA TGAAAAATCT ATCATGCTCC CCTAAAGGAT

2641 CGAAAAATAA CGATATTTAT AAATCGATGC TATTAATCTT ATTTAATGAG TGCAAATTCT

2701 TTCCTAAATA TCCAGTAGAA GAATTAGATA TAACTGCACA ATTGTTTGGA AAATTAATTA

2761 AGCACAATTT ATTAATATCA TATGGTAATA CTTTATCTGT TGTATTAAAA TGTATTTTAG

2821 AAGCTTTAAA AAAAGGATCT GATTCTAAAG TTTTTAATTT TGGGATAACA GCATTAGAGC

2881 AATTTGAAGA CTCTTTAATA TGTTACCCTG CCTTTTTATC TTCCTTAATT CCATTACCTA

2941 CTTTAAGGCA ATATAATCCG CAATATATAA TCCATTGCAA TGAATTATTA AACACACTTC

3001 CAGAACAGTT TAGAACTCTT CCATATATAG ATGCTTCTAC AATACTTAAG ATAAAACACA

3061 TCTCTGAAAT TAGTTCTcct agtagtaaag gagaagaact tttcactgga gttgtcccAA

3121 TTCTTGTTGA ATTAGATGGT GATGTTAATG GGCACAAATT TTCTGTCAGT GGAGAGGGTG

3181 AAGGTGATGC AACATACGGA AAACTTACCC TTAAATTTAT TTGCACTACT GGAAAACTAC

3241 CTGTTCCATG GCCAACACTT GTCACTACTT TCGCGTATGG TCTTCAATGC TTTGCGAGAT

3301 ACCCAGATCA TATGAAACAG CATGACTTTT TCAAGAGTGC CATGCCCGAA GGTTATGTAC

3361 AGGAAAGAAC TATATTTTTC AAAGATGACG GGAACTACAA GACACGTGCT GAAGTCAAGT

3421 TTGAAGGTGA TACCCTTGTT AATAGAATCG AGTTAAAAGG TATTGATTTT AAAGAAGATG

3481 GAAACATTCT TGGACACAAA TTGGAATACA ACTATAACTC ACACAATGTA TACATCATGG

3541 CAGACAAACA AAAGAATGGA ATCAAAGTTA ACTTCAAAAT TAGACACAAC ATTGAAGATG

3601 GAAGCGTTCA ACTAGCAGAC CATTATCAAC AAAATACTCC AATTGGCGAT GGCCCTGTCC

3661 TTTTACCAGA CAACCATTAC CTGTCCACAC AATCTGCCCT TTCGAAAGAT CCCAACGAAA

3721 AGAGAGACCA CATGGTCCTT CTTGAGTTTG TAACAGCTGC TGGGATTACA CATGGCATGG

3781 ATGAACTATA CAAATAAgga tccGTTTTTC TTACTTATAT ATTTATACCA ATTGATTGTA

3841 TTTATAACTG TAAAAATGTG TATGTTGTGT GCATATTTTT TTTTGTGCAT GCACATGCAT

3901 GTAAATAGCT AAAATTATGA ACATTTTATT TTTTGTTCAG AAAAAAAAAA CTTTACACAC

3961 ATAAAATGGC TAGTATGAAT AGCCATATTT TATATAAATT AAATCCTATG AATTTATGAC

4021 CATATTAAAA ATTTAGATAT TTATGGAACA TAATATGTTT GAAACAATAA GACAAAATTA

4081 TTATTATTAT TATTATTTTT ACTGTTATAA TTATGTTGTC TCTTCAATGA TTCATAAATA

4141 GTTGGACTTG ATTTTTAAAA TGTTTATAAT ATGATTAGCA TAGTTAAATA AAAAAAGTTG

4201 AAAAATTAAA AAAAAACATA TAAACACAAA TGATGTTTTT TCCTTCAATT Tcggcgcctg

4261 atgcggtatt ttctccttac gcatctgtgc ggtatttcac accgcatatg gtgcactctc

4321 agtacaatct gctctgatgc cgcatagtta agccagcccc gacacccgcc aacacccgct

4381 gacgcgccct gacgggcttg tctgctcccg gcatccgctt acagacaagc tgtgaccgtc

4441 tccgggagct gcatgtgtca gaggttttca ccgtcatcac cgaaacgcgc gagacgaaag

4501 ggcctcgtga tacgcctatt tttataggtt aatgtcatga taataatggt ttcttagacg

4561 tcaggtggca cttttcgggg aaatgtgcgc ggaaccccta tttgtttatt tttctaaata

4621 cattcaaata tgtatccgct catgagacaa taaccctgat aaatgcttca ataatattga

4681 aaaaggaaga gtatgagtat tcaacatttc cgtgtcgccc ttattccctt ttttgcggca

4741 ttttgccttc ctgtttttgc tcacccagaa acgctggtga aagtaaaaga tgctgaagat

4801 cagttgggtg cacgagtggg ttacatcgaa ctggatctca acagcggtaa gatccttgag

4861 agttttcgcc ccgaagaacg ttttccaatg atgagcactt ttaaagttct gctatgtggc

4921 gcggtattat cccgtattga cgccgggcaa gagcaactcg gtcgccgcat acactattct

4981 cagaatgact tggttgagta ctcaccagtc acagaaaagc atcttacgga tggcatgaca

5041 gtaagagaat tatgcagtgc tgccataacc atgagtgata acactgcggc caacttactt

5101 ctgacaacga tcggaggacc gaaggagcta accgcttttt tgcacaacat gggggatcat

5161 gtaactcgcc ttgatcgttg ggaaccggag ctgaatgaag ccataccaaa cgacgagcgt

5221 gacaccacga tgcctgtagc aatggcaaca acgttgcgca aactattaac tggcgaacta

5281 cttactctag cttcccggca acaattaata gactggatgg aggcggataa agttgcagga

5341 ccacttctgc gctcggccct tccggctggc tggtttattg ctgataaatc tggagccggt

5401 gagcgtgggt ctcgcggtat cattgcagca ctggggccag atggtaagcc ctcccgtatc

5461 gtagttatct acacgacggg gagtcaggca actatggatg aacgaaatag acagatcgct

5521 gagataggtg cctcactgat taagcattgg taactgtcag accaagttta ctcatatata

5581 ctttagattg atttaaaact tcatttttaa tttaaaagga tctaggtgaa gatccttttt

5641 gataatctca tgaccaaaat cccttaacgt gagttttcgt tccactgagc gtcagacccc

5701 gtagaaaaga tcaaaggatc ttcttgagat cctttttttc tgcgcgtaat ctgctgcttg

5761 caaacaaaaa aaccaccgct accagcggtg gtttgtttgc cggatcaaga gctaccaact

5821 ctttttccga aggtaactgg cttcagcaga gcgcagatac caaatactgt ccttctagtg

5881 tagccgtagt taggccacca cttcaagaac tctgtagcac cgcctacata cctcgctctg

5941 ctaatcctgt taccagtggc tgctgccagt ggcgataagt cgtgtcttac cgggttggac

6001 tcaagacgat agttaccgga taaggcgcag cggtcgggct gaacgggggg ttcgtgcaca

6061 cagcccagct tggagcgaac gacctacacc gaactgagat acctacagcg tgagcattga

6121 gaaagcgcca cgcttcccga agggagaaag gcggacaggt atccggtaag cggcagggtc

6181 ggaacaggag agcgcacgag ggagcttcca gggggaaacg cctggtatct ttatagtcct

6241 gtcgggtttc gccacctctg acttgagcgt cgatttttgt gatgctcgtc aggggggcgg

6301 agcctatgga aaaacgccag caacgcggcc tttttacggt tcctggcctt ttgctggcct

6361 tttgctcaca tgttctttcc tgcgttatcc cctgattctg tggataaccg tattaccgcc

6421 tttgagtgag ctgataccgc tcgccgcagc cgaacgaccg agcgcagcga gtcagtgagc

6481 gaggaagcgg aagagcgccc aatacgcaaa ccgcctctcc ccgcgcgttg gccgattcat

6541 taatgcagct ggcacgacag gtttcccgac tggaaagcgg gcagtgagcg caacgcaatt

6601 aatgtgagtt agctcactca ttaggcaccc caggctttac actttatgct tccggctcgt

6661 atgttgtgtg gaattgtgag cggataacaa tttcacacag gaaacagcta tgaccatgat

6721 tacgccaagc ttgataattc ctgcagccca gcttaattct tttcgagctc tttatgctta

6781 agtttacaat ttaatattca tactttaagt attttttgta gtatcctaga tattgtgctt

6841 taaatgctca cccctcaaag caccagtaat attttcatcc actgaaatac cattaaattt

6901 tcaaaaaaat actatgcata taatgttata catataaaca taaaacgcca tgtaaatcaa

6961 aaaatatata aaaatatgta taaaaataaa tatgcactaa atataagcta attatgcata

7021 aaaattaaag tgccctttat taactagcta gtcgtaatta tttatatttc tatgttataa

7081 aaaaatcctc atataataat ataattaata tatgtaatgt tttttttatt ttataatttt

7141 aatataaaat aatatgtaaa ttaattcaaa aaataaatat aattgttgtg aaacaaaaaa

7201 cgtaattttt tcatttgcct tcaaaattta aatttatttt aatatttcct aaaatatata

7261 tactttgtgt ataaatatat aaaaatatat atttgcttat aaataaataa aaaattttat

7321 aaaacatagg gggatccatg gttggttcgc taaactgcat cgtcgctgtg tcccagaaca

7381 tgggcatcgg caagaacggg gacctgccct ggccaccgct caggaacgaa tttagatatt

7441 tccagagaat gaccacaacc tcttcagtag aaggtaaaca gaatctggtg attatgggta

7501 agaagacctg gttctccatt cctgagaaga atcgaccttt aaagggtaga attaatttag

7561 ttctcagcag agaactcaag gaacctccac aaggagctca ttttctttcc agaagtctag

7621 atgatgcctt aaaacttact gaacaaccag aattagcaaa taaagtagac atggtctgga

7681 tagttggtgg cagttctgtt tataaggaag ccatgaatca cccaggccat cttaaactat

7741 ttgtgacaag gatcatgcaa gactttgaaa gtgacacgtt ttttccagaa attgatttgg

7801 agaaatataa acttctgcca gaatacccag gtgttctctc tgatgtccag gaggagaaag

7861 gcattaagta caaatttgaa gtatatgaga agaatgatta aggatcccgt ttttcttact

7921 tatatattta taccaattga ttgtatttat aactgtaaaa atgtgtatgt tgtgtgcata

7981 tttttttttg tgcatgcaca tgcatgtaaa tagctaaaat tatgaacatt ttattttttg

8041 ttcagaaaaa aaaaacttta cacacataaa atggctagta tgaatagcca tattttatat

8101 aaattaaatc ctatgaattt atgaccatat taaaaattta gatatttatg gaacataata

8161 tgtttgaaac aataagacaa aattattatt attattatta tttttactgt tataattatg

8221 ttgtctcttc aatgattcat aaatagttgg acttgatttt taaaatgttt ataatatgat

8281 tagcatagtt aaataaaaaa agttgaaaaa ttaaaaaaaa acatataaac acaaatgatg

8341 ttttttcctt caatttcgat g

//

>Py1299_PyNOT1-G_DTTPbd

LOCUS pSL1299 9313 bp DNA circular 21-APR-2021

SOURCE

ORGANISM

COMMENT Lindner Lab - used to create Py1299 = Py17XNL PyNOT1-G replaces

TTP-binding domain with GFPmut2

COMMENT

COMMENT ApEinfo:methylated:1

FEATURES Location/Qualifiers

exon 5165..5728

/vntifkey="61"

/locus_tag="hDHFR"

/label="hDHFR"

/ApEinfo_label="hDHFR"

/ApEinfo_fwdcolor="pink"

/ApEinfo_revcolor="pink"

/ApEinfo_graphicformat="arrow_data {{0 1 2 0 0 -1} {} 0}

width 5 offset 0"

CDS 7791..8501

/vntifkey="4"

/locus_tag="GFPmut2"

/label="GFPmut2"

/ApEinfo_label="GFPmut2"

/ApEinfo_fwdcolor="pink"

/ApEinfo_revcolor="pink"

/ApEinfo_graphicformat="arrow_data {{0 1 2 0 0 -1} {} 0}

width 5 offset 0"

misc_feature 1631..2078

/locus_tag="PbDHFR/TS\3'UTR"

/label="PbDHFR/TS\3'UTR"

/ApEinfo_label="PbDHFR/TS\3'UTR"

/ApEinfo_fwdcolor="#ff0000"

/ApEinfo_revcolor="green"

/ApEinfo_graphicformat="arrow_data {{0 1 2 0 0 -1} {} 0}

width 5 offset 0"

rep_origin 3475..4157

/locus_tag="ColE1 origin"

/label="ColE1 origin"

/ApEinfo_label="ColE1 origin"

/ApEinfo_fwdcolor="gray50"

/ApEinfo_revcolor="gray50"

/ApEinfo_graphicformat="arrow_data {{0 1 2 0 0 -1} {} 0}

width 5 offset 0"

CDS 2718..3377

/locus_tag="AmpR"

/label="AmpR"

/ApEinfo_label="AmpR"

/ApEinfo_fwdcolor="yellow"

/ApEinfo_revcolor="yellow"

/ApEinfo_graphicformat="arrow_data {{0 1 2 0 0 -1} {} 0}

width 5 offset 0"

misc_feature join(4561..4870,4875..5159)

/locus_tag="PbEF1a 5'UTR"

/label="PbEF1a 5'UTR"

/ApEinfo_label="PbEF1a 5'UTR"

/ApEinfo_fwdcolor="#804040"

/ApEinfo_revcolor="#804040"

/ApEinfo_graphicformat="arrow_data {{0 1 2 0 0 -1} {} 0}

width 5 offset 0"

misc_feature 5736..6183

/locus_tag="PbDHFR-TS 3'UTR"

/label="PbDHFR-TS 3'UTR"

/ApEinfo_label="PbDHFR-TS 3'UTR"

/ApEinfo_fwdcolor="cyan"

/ApEinfo_revcolor="green"

/ApEinfo_graphicformat="arrow_data {{0 1 2 0 0 -1} {} 0}

width 5 offset 0"

misc_feature 8523..9300

/locus_tag="NOT1G 3' Homology Arm (Coding Seq for

AA200-462)"

/label="NOT1G 3' Homology Arm (Coding Seq for AA200-462)"

/ApEinfo_label="NOT1G 3' Homology Arm (Coding Seq for

AA200-462)"

/ApEinfo_fwdcolor="cyan"

/ApEinfo_revcolor="green"

/ApEinfo_graphicformat="arrow_data {{0 1 2 0 0 -1} {} 0}

width 5 offset 0"

misc_feature 15..742

/locus_tag="PyNOT1-G 5' Homology Arm"

/label="PyNOT1-G 5' Homology Arm"

/ApEinfo_label="PyNOT1-G 5' Homology Arm"

/ApEinfo_fwdcolor="cyan"

/ApEinfo_revcolor="green"

/ApEinfo_graphicformat="arrow_data {{0 0.5 0 1 2 0 0 -1 0

-0.5} {} 0} width 5 offset 0"

misc_feature 6218..7784

/locus_tag="PyNOT1-G Promoter and 5'UTR"

/label="PyNOT1-G Promoter and 5'UTR"

/ApEinfo_label="PyNOT1-G Promoter and 5'UTR"

/ApEinfo_fwdcolor="cyan"

/ApEinfo_revcolor="green"

/ApEinfo_graphicformat="arrow_data {{0 0.5 0 1 2 0 0 -1 0

-0.5} {} 0} width 5 offset 0"

ORIGIN

1 cctaggcgag atctCCCCCC TATAATAAAA AACCCCATAC ACTTATTTAG ATTTCTACAA

61 TATATCTTAT CATAACATTT ACTTTTTCTC ATTAACTTTG TTTATCAACT AACATTATTA

121 TATTTATAAA AATATATTAT CATTATTATT CTTAGTTCTA ATGTATATGC TTTTTAATTT

181 AATAATATGA GAATTATAAA AAAAACAAAT TCTAGGGGGG AAATTAAAGT GGATTCGCAT

241 AATCCTTAAA CGAAATATTC TCATAACATA AAACCATGGT AAAAGCCATT GATTATTCTT

301 ATAAAAATAT TAAGAAATAT ATTAACTTAA TCATTATGTC TGTACATTTA TATATATATA

361 ACTGCATATA TATGATAAGC ATTTTTTCTA CATAAAAATT GTGGTTTCTC GTTATTGTAT

421 TTGAGAATAA TATACCCCTT TTATTCCTTT TCATAAAATA TCTTATTACT AAAGTTCAGA

481 AAAATTATAT ATTTTTTAAT TTTAAAATAA AAGCATAACA AATTATATGT ATAGGCTAAC

541 GCAAAAAAAA TAATCTTTAA CATATGTATT AAAAAAATAA TAAGACTTAT ATTTATTTTA

601 AGTGATGAGT ATATATAATC ATAATTATAA TCAATTTTAA CTGTCCCTAT TTAAACAAAT

661 AAAATCACCG TAGTTGAGTA TAGGATAGAA CATTTGTTCA TTCTCTTACT GCTCACAAAA

721 AATATAAATA TAGAACAAAT ACacgcgtgg tggcggtctg aacgacatct tcgaggctca

781 gaaaatcgaa tggcacgaag gcggtggcct caatgatata tttgaagcgc agaagatcga

841 gtggcatgag ggtggcggtc tgaatgacat attcgaagcg cagaagatcg agtggcacga

901 gtaacctagt agtaaaggag aagaactttt cactggagtt gtcccAATTC TTGTTGAATT

961 AGATGGTGAT GTTAATGGGC ACAAATTTTC TGTCAGTGGA GAGGGTGAAG GTGATGCAAC

1021 ATACGGAAAA CTTACCCTTA AATTTATTTG CACTACTGGA AAACTACCTG TTCCATGGCC

1081 AACACTTGTC ACTACTTTCG CGTATGGTCT TCAATGCTTT GCGAGATACC CAGATCATAT

1141 GAAACAGCAT GACTTTTTCA AGAGTGCCAT GCCCGAAGGT TATGTACAGG AAAGAACTAT

1201 ATTTTTCAAA GATGACGGGA ACTACAAGAC ACGTGCTGAA GTCAAGTTTG AAGGTGATAC

1261 CCTTGTTAAT AGAATCGAGT TAAAAGGTAT TGATTTTAAA GAAGATGGAA ACATTCTTGG

1321 ACACAAATTG GAATACAACT ATAACTCACA CAATGTATAC ATCATGGCAG ACAAACAAAA

1381 GAATGGAATC AAAGTTAACT TCAAAATTAG ACACAACATT GAAGATGGAA GCGTTCAACT

1441 AGCAGACCAT TATCAACAAA ATACTCCAAT TGGCGATGGC CCTGTCCTTT TACCAGACAA

1501 CCATTACCTG TCCACACAAT CTGCCCTTTC GAAAGATCCC AACGAAAAGA GAGACCACAT

1561 GGTCCTTCTT GAGTTTGTAA CAGCTGCTGG GATTACACAT GGCATGGATG AACTATACAA

1621 ATAAggatcc GTTTTTCTTA CTTATATATT TATACCAATT GATTGTATTT ATAACTGTAA

1681 AAATGTGTAT GTTGTGTGCA TATTTTTTTT TGTGCATGCA CATGCATGTA AATAGCTAAA

1741 ATTATGAACA TTTTATTTTT TGTTCAGAAA AAAAAAACTT TACACACATA AAATGGCTAG

1801 TATGAATAGC CATATTTTAT ATAAATTAAA TCCTATGAAT TTATGACCAT ATTAAAAATT

1861 TAGATATTTA TGGAACATAA TATGTTTGAA ACAATAAGAC AAAATTATTA TTATTATTAT

1921 TATTTTTACT GTTATAATTA TGTTGTCTCT TCAATGATTC ATAAATAGTT GGACTTGATT

1981 TTTAAAATGT TTATAATATG ATTAGCATAG TTAAATAAAA AAAGTTGAAA AATTAAAAAA

2041 AAACATATAA ACACAAATGA TGTTTTTTCC TTCAATTTcg gcgcctgatg cggtattttc

2101 tccttacgca tctgtgcggt atttcacacc gcatatggtg cactctcagt acaatctgct

2161 ctgatgccgc atagttaagc cagccccgac acccgccaac acccgctgac gcgccctgac

2221 gggcttgtct gctcccggca tccgcttaca gacaagctgt gaccgtctcc gggagctgca

2281 tgtgtcagag gttttcaccg tcatcaccga aacgcgcgag acgaaagggc ctcgtgatac

2341 gcctattttt ataggttaat gtcatgataa taatggtttc ttagacgtca ggtggcactt

2401 ttcggggaaa tgtgcgcgga acccctattt gtttattttt ctaaatacat tcaaatatgt

2461 atccgctcat gagacaataa ccctgataaa tgcttcaata atattgaaaa aggaagagta

2521 tgagtattca acatttccgt gtcgccctta ttcccttttt tgcggcattt tgccttcctg

2581 tttttgctca cccagaaacg ctggtgaaag taaaagatgc tgaagatcag ttgggtgcac

2641 gagtgggtta catcgaactg gatctcaaca gcggtaagat ccttgagagt tttcgccccg

2701 aagaacgttt tccaatgatg agcactttta aagttctgct atgtggcgcg gtattatccc

2761 gtattgacgc cgggcaagag caactcggtc gccgcataca ctattctcag aatgacttgg

2821 ttgagtactc accagtcaca gaaaagcatc ttacggatgg catgacagta agagaattat

2881 gcagtgctgc cataaccatg agtgataaca ctgcggccaa cttacttctg acaacgatcg

2941 gaggaccgaa ggagctaacc gcttttttgc acaacatggg ggatcatgta actcgccttg

3001 atcgttggga accggagctg aatgaagcca taccaaacga cgagcgtgac accacgatgc

3061 ctgtagcaat ggcaacaacg ttgcgcaaac tattaactgg cgaactactt actctagctt

3121 cccggcaaca attaatagac tggatggagg cggataaagt tgcaggacca cttctgcgct

3181 cggcccttcc ggctggctgg tttattgctg ataaatctgg agccggtgag cgtgggtctc

3241 gcggtatcat tgcagcactg gggccagatg gtaagccctc ccgtatcgta gttatctaca

3301 cgacggggag tcaggcaact atggatgaac gaaatagaca gatcgctgag ataggtgcct

3361 cactgattaa gcattggtaa ctgtcagacc aagtttactc atatatactt tagattgatt

3421 taaaacttca tttttaattt aaaaggatct aggtgaagat cctttttgat aatctcatga

3481 ccaaaatccc ttaacgtgag ttttcgttcc actgagcgtc agaccccgta gaaaagatca

3541 aaggatcttc ttgagatcct ttttttctgc gcgtaatctg ctgcttgcaa acaaaaaaac

3601 caccgctacc agcggtggtt tgtttgccgg atcaagagct accaactctt tttccgaagg

3661 taactggctt cagcagagcg cagataccaa atactgtcct tctagtgtag ccgtagttag

3721 gccaccactt caagaactct gtagcaccgc ctacatacct cgctctgcta atcctgttac

3781 cagtggctgc tgccagtggc gataagtcgt gtcttaccgg gttggactca agacgatagt

3841 taccggataa ggcgcagcgg tcgggctgaa cggggggttc gtgcacacag cccagcttgg

3901 agcgaacgac ctacaccgaa ctgagatacc tacagcgtga gcattgagaa agcgccacgc

3961 ttcccgaagg gagaaaggcg gacaggtatc cggtaagcgg cagggtcgga acaggagagc

4021 gcacgaggga gcttccaggg ggaaacgcct ggtatcttta tagtcctgtc gggtttcgcc

4081 acctctgact tgagcgtcga tttttgtgat gctcgtcagg ggggcggagc ctatggaaaa

4141 acgccagcaa cgcggccttt ttacggttcc tggccttttg ctggcctttt gctcacatgt

4201 tctttcctgc gttatcccct gattctgtgg ataaccgtat taccgccttt gagtgagctg

4261 ataccgctcg ccgcagccga acgaccgagc gcagcgagtc agtgagcgag gaagcggaag

4321 agcgcccaat acgcaaaccg cctctccccg cgcgttggcc gattcattaa tgcagctggc

4381 acgacaggtt tcccgactgg aaagcgggca gtgagcgcaa cgcaattaat gtgagttagc

4441 tcactcatta ggcaccccag gctttacact ttatgcttcc ggctcgtatg ttgtgtggaa

4501 ttgtgagcgg ataacaattt cacacaggaa acagctatga ccatgattac gccaagcttg

4561 ataattcctg cagcccagct taattctttt cgagctcttt atgcttaagt ttacaattta

4621 atattcatac tttaagtatt ttttgtagta tcctagatat tgtgctttaa atgctcaccc

4681 ctcaaagcac cagtaatatt ttcatccact gaaataccat taaattttca aaaaaatact

4741 atgcatataa tgttatacat ataaacataa aacgccatgt aaatcaaaaa atatataaaa

4801 atatgtataa aaataaatat gcactaaata taagctaatt atgcataaaa attaaagtgc

4861 cctttattaa ctagctagtc gtaattattt atatttctat gttataaaaa aatcctcata

4921 taataatata attaatatat gtaatgtttt ttttatttta taattttaat ataaaataat

4981 atgtaaatta attcaaaaaa taaatataat tgttgtgaaa caaaaaacgt aattttttca

5041 tttgccttca aaatttaaat ttattttaat atttcctaaa atatatatac tttgtgtata

5101 aatatataaa aatatatatt tgcttataaa taaataaaaa attttataaa acataggggg

5161 atccatggtt ggttcgctaa actgcatcgt cgctgtgtcc cagaacatgg gcatcggcaa

5221 gaacggggac ctgccctggc caccgctcag gaacgaattt agatatttcc agagaatgac

5281 cacaacctct tcagtagaag gtaaacagaa tctggtgatt atgggtaaga agacctggtt

5341 ctccattcct gagaagaatc gacctttaaa gggtagaatt aatttagttc tcagcagaga

5401 actcaaggaa cctccacaag gagctcattt tctttccaga agtctagatg atgccttaaa

5461 acttactgaa caaccagaat tagcaaataa agtagacatg gtctggatag ttggtggcag

5521 ttctgtttat aaggaagcca tgaatcaccc aggccatctt aaactatttg tgacaaggat

5581 catgcaagac tttgaaagtg acacgttttt tccagaaatt gatttggaga aatataaact

5641 tctgccagaa tacccaggtg ttctctctga tgtccaggag gagaaaggca ttaagtacaa

5701 atttgaagta tatgagaaga atgattaagg atcccgtttt tcttacttat atatttatac

5761 caattgattg tatttataac tgtaaaaatg tgtatgttgt gtgcatattt ttttttgtgc

5821 atgcacatgc atgtaaatag ctaaaattat gaacatttta ttttttgttc agaaaaaaaa

5881 aactttacac acataaaatg gctagtatga atagccatat tttatataaa ttaaatccta

5941 tgaatttatg accatattaa aaatttagat atttatggaa cataatatgt ttgaaacaat

6001 aagacaaaat tattattatt attattattt ttactgttat aattatgttg tctcttcaat

6061 gattcataaa tagttggact tgatttttaa aatgtttata atatgattag catagttaaa

6121 taaaaaaagt tgaaaaatta aaaaaaaaca tataaacaca aatgatgttt tttccttcaa

6181 tttcgatggg taccCCGCGG TGgcggccgc TctcgagGAG TATAGGATAG AACATTTGTT

6241 CATTCTCTTA CTGCTCACAA AAAATATAAA TATAGAACAA ATACATATAT AAAAATATAT

6301 CTACACACAT ATATATACAT GTATGAACAT AAAACATATG TACACAATAT AAATGAACTT

6361 GAGTGGAAAA TTAATGTCAT AAGAATATTG TATATGTATT TTGGCAAATA ATATTAATTA

6421 TTTAAATTGG AGTATAAAAA TCATCCCAAA ATAAACTAAA AATACACACA TTAATAAGAT

6481 TAGTTAAAAA AATATATATT GTTAAATAAA TACCTAGAAT TAAAAAGGGG TCATATATAT

6541 CCCTAATATA TACAATATAA AATATAAAAA CGTATGAAGC GAGTTACGAA ATTGTACTAC

6601 TTTTTTATCC CCTCGTTTCC TGTTAACATT AAATAAACAT GACAAAAAAA AGAAAATAAT

6661 CATAAAAAAT ATTGCAGAAT TTACACTTTT AATCTTTTTA TATATATTAT TTATTTTGCA

6721 AATAAAATAA TAAAAAACGC TTTTACCAAA CCTAATATAA CATGAAAATT GTTATGAGAA

6781 ATAATGTATA AAAAAGGAAA ATACAATTCA AAAATCGTAA ACCTTAAAAA AAGGTATATA

6841 AATATTCACC ATATTAATAA GCAATTCATA AAATGTAAAA GAACAGTGTA TGCGCAATTA

6901 TGAACAAATT ATTACCTAGT TTATTTATCT ACAAACGTAT GCCCACAATT TCAACTATAC

6961 AATGTGTTAT ATGTATAATA AATATTTGTA TACAATATAT ACTTATTAAT ATAATACAAT

7021 ATATATATAT ATATACATAA TTAATATATT TTTAGTGCAT ATAAATGTAC TTATTTAATT

7081 CGTAAAAATA TTGCTAATTA AACCCCAATA TTGTATACAT ATACTAAGGA CATAAATTCC

7141 TCTACGGTTT TATTCACATG CATTAAAAAA ATATAACTTA AGTATATTTT TTCTGTATAT

7201 CCCCAAAAAA AAAAATGTGG TCAAAAAAAA TATTATATAT ATATAATTAA TTTTATAGGA

7261 AAAAAACATT CCCAATAAAA TTAAAAAAAA TTCTCAATTT TTTTAATATT ATTTTCCTTT

7321 TTGGTGATAT TCATATATAG TTAATGAATA TAACATAACA CCAATTTATA ATCCTGCGTT

7381 TACTTTATAT GTAAAGTTTT CATAAAATCA TAATAACTAC GCAGTTATAA TAAGTTAAAA

7441 AGGGAAAATG AAAAGAAAAA AAATATATTT TTCTATATGT GTGTGCATAT ATATTGTATA

7501 TATATATTTA TAATCATAAA CAAAATAAGG CAAAAAAAAA TACCAAAAAA AAATAATAAA

7561 AAATAAAATA AATAAATAAC CTCCAAAAAA ATAGAAAACA ATCCAAACAA GCCAAACATA

7621 CCAAACATAC CAAACTTAGC AATCACAGCT GAAATTAAAA AAATAACAAA CACACCAAAT

7681 CGTGTAAACG CAAAAAATAC CACATAACAA AACACAGTCA AATACCCATA TATATTGTTC

7741 ATACCATATT GAGGTAATTA CAATAAAACA TGTGCTAAAA AATGactagt agtaaaggag

7801 aagaactttt cactggagtt gtcccaattc ttgttgaatt agatggtgat gttaatgggc

7861 acaaattttc tgtcagtgga gagggtgaag gtgatgcaac atacggaaaa cttaccctta

7921 aatttatttg cactactgga aaactacctg ttccatggcc aacacttgtc actactttcg

7981 cgtatggtct tcaatgcttt gcgagatacc cagatcatat gaaacagcat gactttttca

8041 agagtgccat gcccgaaggt tatgtacagg aaagaactat atttttcaaa gatgacggga

8101 actacaagac acgtgctgaa gtcaagtttg aaggtgatac ccttgttaat agaatcgagt

8161 taaaaggtat tgattttaaa gaagatggaa acattcttgg acacaaattg gaatacaact

8221 ataactcaca caatgtatac atcatggcag acaaacaaaa gaatggaatc aaagttaact

8281 tcaaaattag acacaacatt gaagatggaa gcgttcaact agcagaccat tatcaacaaa

8341 atactccaat tggcgatggc cctgtccttt taccagacaa ccattacctg tccacacaat

8401 ctgccctttc gaaagatccc aacgaaaaga gagaccacat ggtccttctt gagtttgtaa

8461 cagctgctgg gattacacat ggcatggatg aactatacaa agccggcgga agcggcggaa

8521 gcTACAATAA TATTTCGAAC ACAAATATAT CCCATATACC TAACAATGAA ATTAAAAAAA

8581 TATCTGTAAC AAATGTAAAT AATTTAAAGA ATATCAACAA TTTAGTTCCA CATTTATCTA

8641 CCATAGATGT TCCAAATCAA ACTACTACTA ATTTTATTAC CCATAATAAT CTTAGTGATA

8701 ATAATATCGA AAATAGTGTC AATAATAATA TAATTTCTGA ACCCCACAAT GATAACAATA

8761 ATGTTAACAC TATTAATAAC TCAAATCGAA CAGCTAGCTA CATAAGTAAT AATAATCAAA

8821 CTCAATTTAC CAATCTTATG AATAATATTT TGAACCAAAA CAATACTACT ACTACTACTA

8881 ATAACAATAA CAACCTTAAT AATGATAATA TGCTATTATA TATTAACAAT ATTCTACCAA

8941 ACCTCTCAAA TATAACAGAT AAAAATACTA TACCATCAAA TATAAATAAT TTACAAGCCA

9001 TTAATAGAAA TAATCCAATA TATAAAAATA AAAACGCCAA TATTAATTTA AATGCTATAT

9061 CAGCCATTAA TAATATTAAC AATCTTAATA GTGCATATAA TATTAACTTA TTAGACAATA

9121 ATCCCTCTTC TAATATATTA TCACCAAACT CAAATAGAGT CTATCAAAAT AAAGGTTTTA

9181 TTAATACAAA CGAACCAACA CATATTAATA GGAACATAAA TCTAGTAACA AATAATATGA

9241 CACACAATAA AAATTTGAAT TCTACAAACC TCCTAAATAA TACTAATCAA ATTAAAATAG

9301 ATATAAATAT GCC

//
