# Supplementary material for: The Plasmodium NOT1-G paralogue is an essential regulator of sexual stage maturation and parasite transmission
Source: PLoS Biol. 2021 Oct 21;19(10):e3001434. doi: 10.1371/journal.pbio.3001434 (PMC8562791; doi:10.1371/journal.pbio.3001434)

Hart *et al.* Supplemental File 2 - Biological Replicate 1

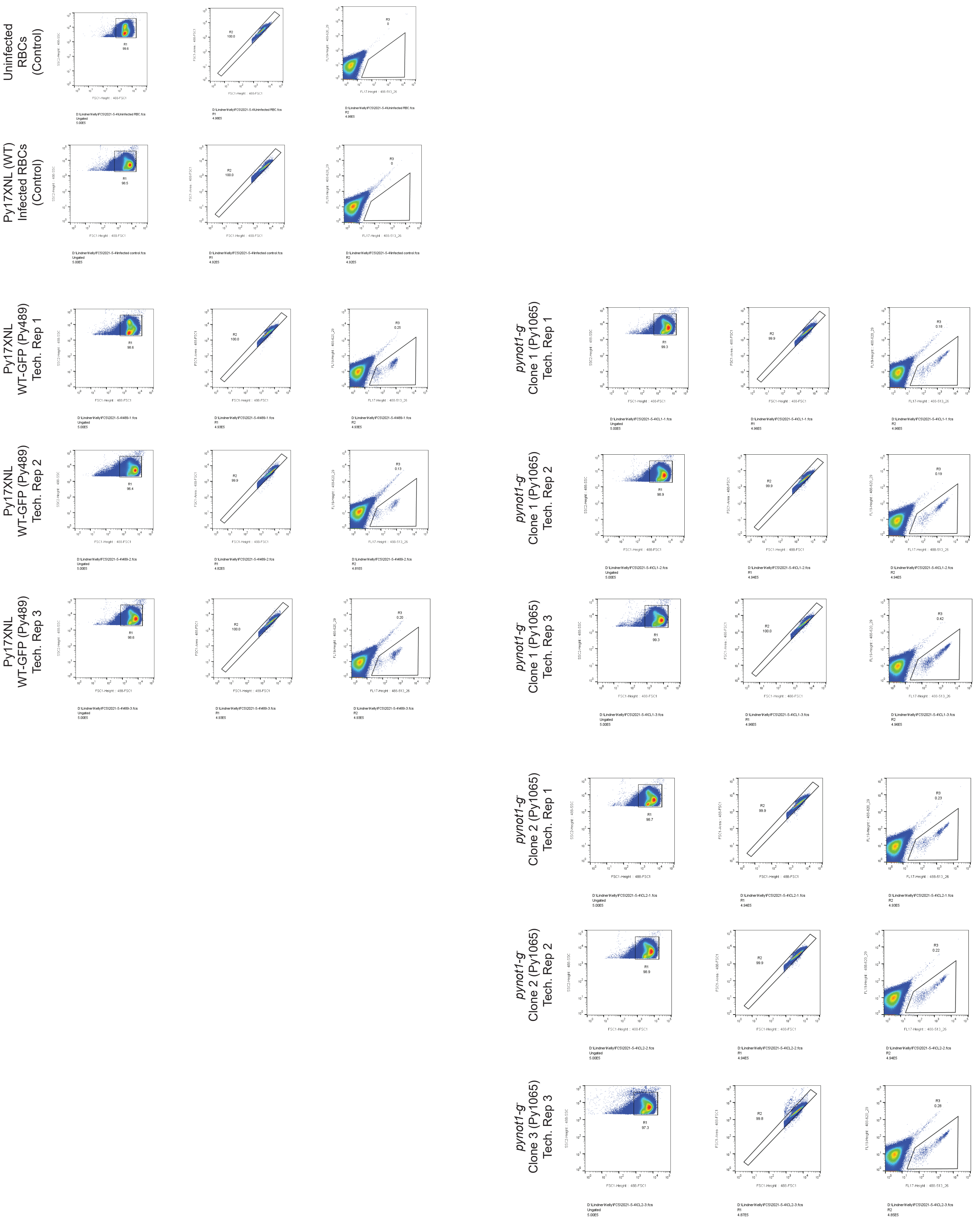

Hart *et al.* Supplemental File 2 - Biological Replicate 2

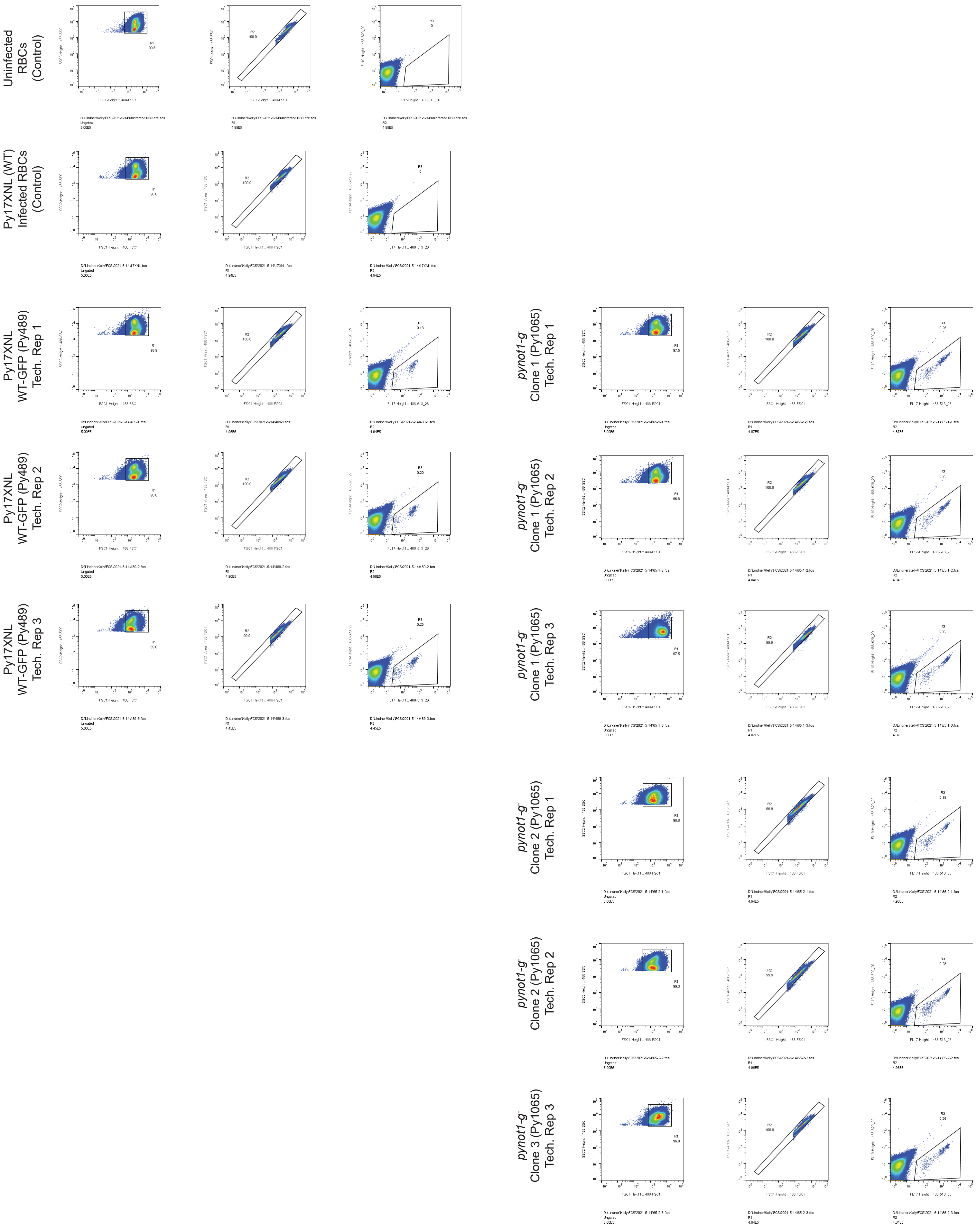

Supplement: S2 File — (PDF) [file pbio.3001434.s012.pdf]
