# Supplementary material for: The Plasmodium NOT1-G paralogue is an essential regulator of sexual stage maturation and parasite transmission
Source: PLoS Biol. 2021 Oct 21;19(10):e3001434. doi: 10.1371/journal.pbio.3001434 (PMC8562791; doi:10.1371/journal.pbio.3001434)
Supplement: Attachment — Submitted filename: 117463_1_rebuttal_2134174_qwprhw.docx [file pbio.3001434.s016.docx]

**Manuscript number:** RC-2021-00852

**Corresponding author(s):** Scott, Lindner

*[Please use this template only if the submitted manuscript should be considered by the affiliate journal as a full revision in response to the points raised by the reviewers.*

*If you wish to submit a preliminary revision with a revision plan, please use our "*[*Revision Plan*](https://docs.google.com/document/u/0/d/1HHp62Tfeb44nd3Gu_3ChB0b8gmMdEog7GjZcZFwHSiQ/edit)*" template.* ***It is important to use the appropriate template to clearly inform the editors of your intentions.****]*

## 1. General Statements [optional]

We are grateful for the constructive and highly supportive reviews provided by our Reviewers. We especially appreciate the efforts they have made to provide suggestions on how to make our revised manuscript even more robust. We have incorporated many of these suggestions into the revised manuscript that will post to Biorxiv and will be submitted to an affiliate journal. We have provided point-by-point responses to each Reviewer below each item (starting with
*Response: …*), along with any changes made in response to that comment/suggestion (starting with *In our revised manuscript, …*).

Finally, we agree with all Reviewers that this work should be of broad interest to the molecular biology, cell biology, and parasitology communities. Our discovery that *Plasmodium* and two related genera have taken the unorthodox approach of duplicating their NOT1 protein, and that *Plasmodium* has dedicated it for its unique transmission strategy, is a fascinating adaptation of the use of this core eukaryotic complex. We believe that those that focus on diverse aspects of RNA biology, including RNA preservation/decay, the maternal to zygotic transition, translational repression, and beyond will find this work to be of interest and relevant to their own research questions.

## 2. Point-by-point description of the revisions

------------------------------------------------------------------------------
Reviewer #1 (Evidence, reproducibility and clarity (Required)):

The manuscript „The Plasmodium NOT1-G paralogue acts as an essential nexus for sexual stage maturation and parasite transmission" investigates the two forms of NOT1 in rodent malaria parasites. The authors found out that the original NOT1 is crucial for gametocyte induction as well as transmission to the mosquito, they therefore renamed it NOT1-G. The paralogous proteins, on the other hand, appears to be crucial for intraerythrocytic growth, since it cannot be knocked out. The authors then investigated NOT1-G in more detail, using standard phenotyping assays. They found a slightly increased gametocytemia and a minor effect on transmission to the mosquito.

*Response: In our submitted manuscript, we do focus on PyNOT1-G because of the exciting role it has for both sexes of gametocytes, which results in a complete defect in transmission to mosquitoes. Our investigations of what domains of PyNOT1-G focused on the most likely suspect: the putative tristetraprolin-binding domain (TTPbd). It was through deletion of this domain that we observed only a minor defect in the prevalence of infection of mosquitoes, indicating that the portion of PyNOT1-G that is required for transmission lies elsewhere (in part or in total). It is also important to correct Reviewer 1’s statement regarding the other (perhaps canonical) PyNOT1. To our surprise, PyNOT1 could be deleted, but resulted in a parasite that has an extreme fitness cost and a very slow growth phenotype. This is in stark contrast to other eukaryotes, where NOT1 is essential.*

Reviewer #1 (Significance (Required)):

If the authors are able to provide convincing data that NOT1-G is indeed important for gametocyte induction and transmission to the mosquito, then the report would be of high significance for the malaria and molecular cell biology fields.

*Response: We have in fact shown this and more in the originally submitted manuscript, and thus we are grateful that Reviewer 1 considers this work to be of high significance in a broad readership (molecular and cell biology, parasitology). In our revised manuscript, we have added text throughout to make these results even more apparent and clear for the reader.*

My expertise: molecular cell biology of gametocytes, translational regulation, parasite transmission


Reviewer #2 (Evidence, reproducibility and clarity (Required)):

**Summary**

The manuscript by Hart et al. builds upon a fascinating finding presented in a previous manuscript by the same authors, in which they show that CCR4 seems to be able to associate with two members of the NOT1 family. In this work, the authors first re-annotate the two NOT1 paralogs in Plasmodium yoelii and then perform an in depth characterization of the role of NOT1-G during gametocytogenesis and early mosquito development. Using gene knockout and different genetic crosses, the authors show that NOT1-G is essential for male gametocyte development and leads to an arrest of development in zygotes arising from female gametocytes. Using RNA-seq the authors show that NOT1-G leads to lower transcript abundances, leading to the hypothesis that NOT1-G might be involved in preserving mRNAs in a larger RNA-binding complex. Lastly, the authors characterize a NOT1-G defining TPP domain and find that it is not essential for either male/female phenotype observed for the whole gene KO.

*Response: We appreciate the concise and accurate summary of these findings.*

**Major comments:**

- Are the key conclusions convincing?

The phenotypic characterization of NOT1-G during gametocytogenesis / early mosquito development is nicely presented and the experiments are well performed. Because a duplication of NOT1 with possibly opposing roles of the paralogs is a very unique feature with broad implication on RNA metabolism, it would have been great to see two select experiments on the molecular level adding evidence that 1) NOT1/NOT1-G are mutually exclusive in a complex with CCR4/CAF1 and 2) NOT1-G acts post-transcriptionally in an antagonistic way to NOT1 (i.e. as a mRNA 'stabilizer' as proposed by the authors).

*Response: We agree that inclusion of those two aspects would make for a more complete story about these two NOT1 paralogues.*

*First, we also think that it is highly likely that NOT1 and NOT1-G are mutually exclusive, as in other eukaryotes NOT1 acts as a scaffold protein upon which effector proteins bind and bridging interactions are made. In our original manuscript, we did not include a mention of our previous attempts to address this question through colocalization and proteomic approaches, as they were largely unsuccessful. Specifically, we generated rabbit polyclonal antisera to PyNOT1-G’s tristetraprolin-binding domain but it did not pass our rigorous quality control (e.g. too much staining persisted in pynot1-g- parasites). Using both asexual and sexual blood stage parasites, we also attempted immunoprecipitation (with and without chemical crosslinking) and proximal labeling approaches via BioID and TurboID but all approaches did not produce rigorous results and thus we did not report them in our original manuscript. However, this question of whether the two NOT1 paralogues were mutually exclusive in complexes was also taken up by the Bozdech Laboratory in their 2020 preprint (Liu et al.) where they were able to capture the P. falciparum NOT1-G and NOT1 proteins (called Not1.1 and Not1.2 in that work). While their proteomic evidence showed that they could capture these bait proteins and that the NOT1 paralogues were not in the same complex, these results should be taken with a grain of salt: all mass spectrometry-based proteomic approaches are limited in that an absence of evidence does not mean that the protein is not present/interacting. Moreover, these efforts only identified a few other proteins that were already known to interact with the CAF1/CCR4/NOT complex, but even so, they did not use statistically rigorous methods in an attempt to quantify these results. In our revised our manuscript, we have included additional text to describe our unsuccessful efforts to do these capture proteomics experiments, and we have expanded our discussion of the Liu et al findings that provide some evidence in support of a mutually exclusive complex.*

*Second, we also hypothesize that PyNOT1-G acts post-transcriptionally to affect mRNA abundance and translation. However, it is important to emphasize that NOT1 proteins typically act as scaffolds, with the recruited effector proteins acting to hasten the degradation and/or to preserve associated transcripts. We believe that studying these effector proteins is the next important effort to undertake. In fact, we hypothesized that these antagonistic effector proteins would be analogous to TTP and ELAV/HuR-family proteins as are found in other eukaryotes, and that the critical interaction with PyNOT1-G would be via its putative TTP-binding domain. It was for that reason that we interrogated the TTP-binding domain itself, and were surprised that its deletion did not phenocopy the complete gene deletion. Ongoing work will be focused on identifying these antagonistic effector proteins that likely are expressed in a stage-enriched manner, and to define how they interact with PyNOT1-G in order to direct specific mRNAs to their fates. Additionally, it would be very important and exciting to directly test if PyNOT1 and PyNOT1-G are functionally opposed. However, this would be exceptionally challenging to study from a technical standpoint. While we were able to delete the pynot1 gene after many repeated attempts, these parasites are very sickly and grow very slowly. Because of this, we believe that assessing direct versus indirect effects of PyNOT1 in these cells would not be feasible or robust. Given this, comparing functions between PyNOT1 and PyNOT1-G could not be done in a conclusive manner. In our revised manuscript, we have expanded our descriptions of the mechanisms by which we believe PyNOT1-G and its complex affects mRNA fates. In particular, we have expanded our Discussion section to incorporate the results that indicate that the TTP-binding domain is not required for the essential functions of PyNOT1-G.*

- Should the authors qualify some of their claims as preliminary or speculative, or remove them altogether?

The authors describe the role of NOT1-G as 'preserving' mRNA. The lower abundance of many transcripts in the NOT1-G knockout suggest this, but experimental proof is not provided (see suggestions below). Maybe rephrase to 'putatively preserved/stabilized' or 'has a potentially stabilizing function'. The same is true for the mutually exclusive association of the two paralogs with CCR4/CAF1. The authors refer to a protein co-IP of CCR4 showing that CCR4 can interact with both NOT1 and NOT1-G, but a reciprocal experiment is lacking.

*Response: In our first publication on the deadenylase members of this complex, we also saw a similar effect on specific mRNAs when pyccr4-1 was deleted: the abundance of specific mRNAs went up in pyccr4-1^-^ parasites. In that work and here in this manuscript, we have carefully decided to apply the word “preserved” to the fate of these mRNAs as it describes in a general way what is happening. In order to robustly state that mRNAs are stabilized by PyNOT1-G (directly or indirectly) would require additional experiments designed to test this (more description on this is provided on a response below). Second, as described above, we agree that doing a reciprocal IP for mass spectrometry-based proteomics would be ideal, we attempted four different approaches to do this to no avail. However, the composite proteomics data that is already available in the literature and via the Liu et al. preprint from the Bozdech Lab all indicate that these interactions occur, and perhaps that NOT1 and NOT1-G are mutually exclusive as expected. In our revised manuscript, we have provided further explanation in the Discussion for our use of the descriptor “preserve” instead of “stabilize”, and as noted above, and we have expanded our Discussion to more comprehensively define the interaction network depicted in Figure 7.*

In both cases, the conclusions of the authors are very likely (e.g. downregulation of many genes as seen by RNA-seq), but the final experimental evidence is not provided and a network such as in Figure 7 is not fully supported. If the authors would like to maintain these statements, then they should be rephrased and made clear or the additional experimental evidence suggested below is necessary.

*Response: We hold that the published proteomic datasets do support such a network, with further support offered from the preliminary proteomic evidence from the Liu et al preprint. Therefore, we have not modified our manuscript beyond the additional text now provided in the Discussion as noted above.*

- Would additional experiments be essential to support the claims of the paper? Request additional experiments only where necessary for the paper as it is, and do not ask authors to open new lines of experimentation.

The essential claim that NOT1-G is important for gametocytogenesis and early mosquito development is well presented and fully supported by the experiments. As for the role of NOT1-G in 'preserving' mRNA, an mRNA half-life experiment would be necessary (or the text should be adjusted as mentioned above). In a short-term in vitro culture, pynot1-g- and WT parasites could be treated with ActD and abundances of select transcripts are measured by RT-qPCR.

*Response: We appreciate that Reviewer 2 considers the rigor of our experiments to be high. Regarding the use of the term “preserve” vs “stabilize”, we agree that to shift from our more general descriptor (preserve) to one that has specific connotations (stabilize) would require additional experimentation. To correctly and most robustly make the claim of stabilization would require work on par with that done by Painter et al. (PMID: 29985403) that uses a thiol-containing nucleotide (4-TU) along with a yeast-derived fusion enzyme (yFCU) to convert it for use by Plasmodium. Previously we have shown that an associated deadenylase (PyCCR4-1) also acted to preserve mRNAs, and moreover that deletion of its gene resulted in no discernable effect upon the poly(A) tail or 3’ UTR of an mRNA that is bound by this complex (p28).*

*While understanding mRNA stability is an exciting area of study, this 4-TU labeling experiment alone warranted a standalone, high impact publication for Painter et al. As this has not been adapted for any rodent-infectious Plasmodium species to date, and as adaptation of this labeling approach took several years for Dr. Painter while in the Llinas Laboratory (personal communication), we believe this work is beyond the scope of this study. Moreover, the additional information that it would provide to understand NOT1-g functions (preserve vs stabilize) would be incremental beyond the major storyline presented in this manuscript. In our revised manuscript, we have added text to ensure that our choice of “preserve” is well defined and explained.*

To support the idea that NOT-1 and NOT1-G associate in a mutually exclusive way or to just show that they act in distinct complexes despite their similar expression patterns, an IFA with a double stained NOT1/NOT-1G cell line could be performed. Alternatively, the authors could perform a protein co-IP using the already existing NOT1/NOT1-G-GFP cell line and show that the proteins don't interact with each other or even have certain distinct interaction partners.

*Response: We agree, and these studies were attempted but were unsuccessful (described in our responses above). In our revised manuscript, we have included this information as noted above.*

- Are the suggested experiments realistic in terms of time and resources? It would help if you could add an estimated cost and time investment for substantial experiments.

All necessary cell lines for a NOT1/NOT1-G co-IP and the ActD experiment are already present. The authors already present a ring to schizont in vitro culture (for ActD) and also have substantial experience in protein co-IP and proteomics.

I am not sure about the cost for a proteomics experiment at the author's institute and I don't want to make a guess on time investment given the still on-going COVID situation.

*Response: We agree that these experiments would be interesting, and would be costly to do at a transcriptome-wide scale and would require substantial time to conduct. We believe that the 4-TU approach noted above is the most rigorous, but is well beyond the scope of this study as it has not yet been adapted to rodent-infectious malaria parasites. As noted above, we have attempted four different proteomics approaches to provide reciprocal evidence for the complex composition which were unsuccessful. In our revised manuscript, we have added text to ensure that our choice of “preserve” is well defined and explained, and have noted the unsuccessful reciprocal proteomics approaches.*

- Are the data and the methods presented in such a way that they can be reproduced?

The MM section is well structured and presented and the supplemental material includes all data.

*Response: Thank you. We want to ensure that our work is clearly described and can be reproduced with the information reported.*

- Are the experiments adequately replicated and statistical analysis adequate?

There is hardly any test of significance presented in the main text of the manuscript (e.g. Figure 3B and 4A). Please show the individual data points for these graphs and make sure the n= and the statistical test is described in the figure legend. If you use the term significant in the text, then just add the p-value behind it. This is also true for the RNA-seq data: Genes are sorted by fold-changes, leaving it unclear if these changes are significant. These data are however presented in Table S1 and could be incorporated in the main text.

*Response: We agree. In our revised manuscript, we have incorporated additional details about the statistical tests used, p-values for noteworthy comparisons, and have included more panels for our comparative RNA-seq datasets (heatmap, PCA, MA plots). We have also made adjustments to our plots to make individual data points more readily observed, especially when error bars may block them (e.g. Figure 3B). And as in the original submission, all of the pertinent values, including fold changes, statistics and more are provided in our comprehensive supplementary files. We have structured the Supplementary Tables to flow from one tab to the next with the filtering/threshold applied noted both in the tab name and in the README tab that is found first among the tabs.*

**Minor comments:**

- Specific experimental issues that are easily addressable.

One idea that is also not discussed but could be added is for example that NOT1-G itself doesn't even have a stabilizing effect itself, but act as a decoy for other components of the CCR4/Caf1 complex, keeping them from associating with NOT1. In the NOT1-G knockout, the decrease in RNA abundance might then be just a result of an 'overactivity' of CCR4/Caf1/NOT1.

*Response: This hypothesis proposed by Reviewer 2, that PyNOT1-G is acting as a decoy or a binding partner sponge, is certainly feasible. For this scenario to be effective, PyNOT1-G would need to be in excess of PyNOT1 and/or would need to be able to bind to the critical effector protein(s) better than does PyNOT1. However, our microscopy data, along with the transcriptomic data presented here and previously published proteomic data would indicate that these two gene products are in approximately balanced proportions and are similarly localized. This does not exclude the possibility that PyNOT1-G could act as a sponge for relevant binding partners. In our revised manuscript, we have raised this possibility as an alternate explanation for the phenotype in the Discussion section.*

- Are prior studies referenced appropriately?

Throughout the manuscript, the authors should make clear what results come from which organism. Just as an example, the genome wide KO screens were performed in P. berghei and P. falciparum, CCR4/CAF1 experiments were performed in P. yoelii, whereas the original DDX6 work was done in P. berghei.

*Response: We agree. In our revised manuscript, we have added additional text to further clarify what data comes from which Plasmodium species.*

- Are the text and figures clear and accurate?

The Introduction is a bit long and partially turns into a minireview of eukaryotic RNA degradation. In the main text on page 13, the authors introduce a model for proteins involved in translational repression. This in not fully accurate, since for many of the proteins in this network, an effect on translation has actually not been shown. This includes NOT1-G characterized in the present work that most likely has an effect on mRNA stability, but for which a role in regulating translation is not presented.

*Response: We believe the length and content of this Introduction is appropriate to provide the context that some readers outside of the parasitology field will need to appreciate these findings. Regarding designations for these proteins as being related to translational repression, we think that the ample proteomic evidence tying them to translationally repressive complexes warrants this. In our revised manuscript, we have made it more clear that these proteins themselves have not been directly implicated in translational repression.*

- Do you have suggestions that would help the authors improve the presentation of their data and conclusions?

Overall the RNA-seq is underrepresented and Figure 5 could easily be expanded by adding several panels that would help the future reader getting a better idea of the data:
1. Summary graphs such as PCA/MDS plots of the different replicates and MA-plots (all of which can be easily generated in DESeq2)
2. Heatmaps comparing the expression patterns of pynot1-g-, pbdozi-, pbcith-, pyalba4- highlighting some key gametocyte genes mentioned in the text
3. Alternatively to 2., a simple Venn Diagram would already be very informative

An informative representation might also be to sort the differentially expressed genes as predominant male and/or female. The P. berghei data by Yeoh et al (PMID: 28923023) could be a starting point.

*Response: We agree. In our revised manuscript, we have expanded Figure 5 to include additional plots that speak the rigor of these datasets. Specifically, we have added a comprehensive heatmap and PCA plots, as well as MA plots as recommended. We have chosen not to include a Venn diagram for the overlap of affected mRNAs across these transgenic parasite lines, as we hold that this information is best provided in the text (high level observations) and the Supplement (details).*


Reviewer #2 (Significance (Required)):

**Describe the nature and significance of the advance (e.g. conceptual, technical, clinical) for the field.**

Technically this manuscript builds on standard methods of the field that are well executed. There is no direct clinical advancement, although one might argue that a unique adaptation of the parasite could always be a novel therapeutic target.
Conceptually this is great advancement for the parasitology field as it is, providing additional evidence for the importance of post-transcriptional regulation for parasite transmission. With the two experiments suggested above and the additional evidence gained from it, this manuscript could also gain great interest to readers outside the field by clearly showing how alternative ways to regulate RNA stability evolved.

*Response: We are grateful for your careful review of our work and for the recommendations that you provided. We have incorporated many of them into the revised manuscript to make it even more rigorous and comprehensive. We also appreciate hearing that this work would be of great interest to a broader community. We feel that this is already the case, as the duplication of NOT1 and the dedication of one paralogue to an essential function is exciting and novel among eukaryotes.*

**Place the work in the context of the existing literature (provide references, where appropriate)**

The work builds on the early reports of the particular RNA metabolism in gametocytes performed in the groups of Andy Waters. Since then, the authors themselves have published a great set of manuscripts extending our knowledge of the proteins involved in gametocytogenesis and nicely place the current work into this framework.

*Response: We appreciate this positive feedback. This is a fascinating topic to study.*

**State what audience might be interested in and influenced by the reported findings.**

The manuscript as it stands is particularly interesting for the parasitology and potentially the evolutionary biology field. For a broader readership for example in the RNA field, the possibly antagonistic roles and mutually exclusive association with CAF1/CCR4 are likely most interesting.

*Response: We agree that this should be interesting to readers beyond our own field, as the duplication and specialization of NOT1, and the finding that the “canonical” PyNOT1 can be deleted, are both of general interest to how eukaryotes have adapted and deployed a highly conserved and essential RNA metabolic complex.*

**Define your field of expertise with a few keywords to help the authors contextualize your point of view. Indicate if there are any parts of the paper that you do not have sufficient expertise to evaluate.**

**Expertise:**

RNA biology, Plasmodium falciparum, Bioinformatics


Reviewer #3 (Evidence, reproducibility and clarity (Required)):

In this manuscript, the authors investigate the requirement of two possible Not1 paralogs for the development of asexual blood stages and for the sexual transmission stages of Plasmodium yoelii. While Not1 is critical for asexual blood stages, its putative paralog, Not1G is important for the development of sexual transmission stages. In the absence of Not1G, male gametes are not formed while female gametes are formed and can be fertilised by wt male gametes. However, the resulting zygote cannot develop further into ookinete. The in vitro genetic cross assay to show this is elegant! A transcriptomic analysis further indicates that the transcriptomes of Not1G deficient parasites are significantly different from their WT counterpart.

*Response: We are thrilled that you found our evidence and approaches to be rigorous and compelling. Thank you.*

**Major comments:**

The discussion section is very nice and the authors describe well what is speculative and should be further confirmed by additional experiments. However, I did find this was not the case in the results section where the authors are proposing conclusions that are not supported by the results. I think the reading of this manuscript would be much more enjoyable if the authors only describe the results shown and move all the discussions to the dedicated section. Below are some examples. The data presented in this manuscript is not showing a nexus, this is a suggestion based on the results of other articles, the word should thus be removed from the title (and kept for a future review!). The last two sentences of the localisation section should be moved to the discussion because they do refer to results not shown in this manuscript. The last sentence of the second paragraph of the zygote development section should also be moved to the discussion. For the transcriptomic analysis there is also no formal comparison with transcriptomes of other previously analysed mutants: the results of the comparisons should either be shown or not discussed in the result section. Finally, the discussions mentioning interactors of the complex should be removed from the result section and moved to the discussion unless the results are formally analysed.

*Response: We again thank you for the complement. In our original manuscript, we opted to provide some limited interpretations and context within the Results section in order to help guide readers along our train-of-thought and line-of-experimentation. While a more traditional split of keeping essentially all discussion and interpretation for the Discussion is a tried-and-true approach, we prefer this more narrative method and have opted to keep these short sections in the Results section.*

I would strongly suggest the author the better present and describe their transcriptomic results. There is only one volcano plot indicating the overall defect in mixed gametocytes in the main figure. Apart from this, the results are only described in the main text or in supplementary tables. It is therefore difficult to understand the subtilities of the analysis. For example, the authors frequently mention dysregulated genes, but without specifying whether it is up or down-regulated in the mutant. To address this issue, I would suggest the authors to better describe their results in the figures. They could show the GO term enrichment analysis they mention and show how they assign GO term or transcripts to male and female parasites. It would also be nice to discuss some of the results a bit more in details. For example, it is not surprising to see a reduction in transcripts that are under the control of AP2-O in retort-arrested ookinetes as the parasite do not reach this stage. It is thus highly speculative to specifically link this observation with ALBA4 without further detailed analysis. On the other hand, it is more surprising to see a decrease in ap2g transcripts, while the authors observe an increased gametocytaemia. Could the authors comment this observation? It may also be nice to better present the comparison between gametocytes and schizonts to possibly speculate on the early requirement of Not1G in committed schizonts.

*Response: We (and Reviewer 2) agree. In our revised manuscript, we have expanded Figure 5 to include additional plots that speak the rigor of these datasets. Specifically, we have added a heatmap, and PCA and MA plots as recommended. We have chosen not to include a Venn diagrams for the overlap of affected mRNAs across these transgenic parasite lines for the reasons stated above in our response to Reviewer 2. Similarly, we have opted to keep the specifics of the GO Term analyses in the Supplement as we believe these should always be taken with a grain of salt (especially high level GO Terms, as many choose to report). Finally, we have expanded our discussion on our observation that pyapiap2-g transcript levels are lower in the pynot1-g- line, despite seeing a slight increase in gametocytemia.*

The conclusion regarding the similar localisation of Not1 and Not1G with other members of the CAF1/CCR4/NOT complex is not really convincing for two reasons. First, there is not colocalization shown and, second, the distribution is not very peculiar so it is difficult to draw any conclusion with this level of resolution. The presence of alpha-tubulin in the nucleus of male gametocytes is also very surprising as it is rather nucleus-excluded in both P. falciparum and P. berghei, could the authors comment this peculiar localisation?

*Response: We agree and disagree here. First, we agree that no colocalization data is presented here to place NOT1-G within the limit of resolution of fluorescence microscopy. What we can (and do) state is that these proteins are all localized to cytosolic puncta, which matches what is observed for essentially all other studied eukaryotes. In further support of this, our published, quantitative proteomic data indicates that the bioinformatically predictable members of the CAF1/CCR4/NOT complex do associate as anticipated. In the same vein, the micrographs presented were not captured by confocal microscopy, and thus the apparent localization of alpha tubulin “in” the nucleus is most likely attributed to being above and/or below the nucleus. Taken together, we do feel that the combined evidence is convincing. As we have already made all of these points in the original manuscript, we have not adjusted the revised manuscript further.*

One of my major frustration when reading this manuscript was that the authors are not trying to discriminate between an early role of Not1G during gametocytogenesis or later in gametogenesis. The fact that the transcriptomes of gametocytes and schizonts seem to show similarities suggests that the phenotype observed during both male gametogenesis or ookinete development are probably linked to early knock-on defects during gametocytogenesis. Could the authors test whether male gametocytes replicate DNA or female activate translation? These are of course non-essential experiments as the authors are careful with their conclusions and mention possible defects during both gametocytogenesis or gametogenesis. Addressing this question may however add significant insights into the requirement for Not1G.

*Response: We are sorry for the frustration. We wrote the manuscript so as to state what we feel we could robustly say, and where we are drawn to speculate, we made that speculation clear. As Reviewer 3 notes, we have not attempted to discriminate between functions that PyNOT1-G may be playing in different stages or substages of development because we do not believe the experiments allow that discrimination. While we could investigate finer and finer aspects of possible defects in both male and female gametocyte development, the most impactful take home messages remain the same. We continue to address questions related to translational repression and its release, and anticipate that PyNOT1-G will play a substantial and essential role in this. As Reviewer 3 noted, we have already discussed these possibilities in the original manuscript, and thus have not added anything further about this in our revised manuscript.*

**Minor comments:**

Please use page and line numbering for your next submissions!
Please describe what "bioinformatics" was used.
I would show the nice localisation in oocyst and sporozoite in the main section.
The conclusions drawn from the genetic cross seem to come from a single biological replicate, if this is the case please indicate it clearly.

*Response: We apologize for these oversights. In our revised manuscript, we have provided page and line numbering, have expanded on what bioinformatic processes were done in the manuscript, and have made it more clear that the genetic crosses come from multiple biological replicates (biological triplicate for the transmission-based genetic cross, biological duplicate for the* in vitro *culture genetic cross). However, we have opted to retain the oocyst and sporozoite IFA data in the Supplement, as the rest of the story is focused on blood stage and early mosquito stage.*


Reviewer #3 (Significance (Required)):

This manuscript highlights the requirement of a Not1 paralog in the transmission stages of a Plasmodium parasite. More specifically it describes a new player in the control of RNA biology during this process where our knowledge is scarce. It will be a valuable manuscript for molecular parasitologists interested in transmission or RNA biology.

*Response: We agree and are grateful that our colleagues find this study to be a valuable addition in our efforts to understand how malaria parasites have adapted classic eukaryotic mechanisms to suit their purposes.*

Our expertise is largely in molecular and cellular parasitology.
