# Supplementary material for: The Plasmodium NOT1-G paralogue is an essential regulator of sexual stage maturation and parasite transmission
Source: PLoS Biol. 2021 Oct 21;19(10):e3001434. doi: 10.1371/journal.pbio.3001434 (PMC8562791; doi:10.1371/journal.pbio.3001434)
Supplement: Attachment — Submitted filename: Hart et al PLOS Biology Response to Reviewers (rev2).docx [file pbio.3001434.s018.docx]

**Response to Reviewers, Revision 2, Hart *et al*. 2021**

We appreciate this additional feedback and suggestions from the Reviewers and Academic Editor, and we have revised our manuscript to address these comments. We have provided point-by-point responses to each Reviewer and Editor remark below each item in italics (starting with *Response: …*).

*****************************************************

REVIEWS:

Reviewer #2: Sebastian Baumgarten. Plasmodium RNA biology.

Reviewer #3: Molecular parasitology.

Reviewer #2: The authors have addressed all my previous comments and I am very much looking forward to seeing this work published.

Two minor comments: Please add the datapoints to the bar graph in Figure 4A and make sure that the quality of the figures is ok (which could also be the manuscript review system)

*Response: We have converted the bar graph (Figure 4A) to a graph of individual values for each biological replicate with the SEM provided as error bars. We have also corrected the resolution issue of Figure 5.*

Reviewer #3: This new version has been significantly improved compared with the previous one.

I however think that:

-	including discussions in the result section is a way to over interpret the results;

*Response: We maintain that the limited descriptions and discussion of the data within the results section helps non-expert readers to understand the logical flow of the experimentation as present, and thus we have maintained it as is.*

-	the visual representation of the transcriptomic results is mainly showing the number of regulated genes but not highlighting the biology behind the dots;

*Response: We have made substantial changes to Figure 5 to provide even more information regarding the biology behind the data presented. Specifically, we have 1) omitted the redundant correlation heatmap and maintained the more informative PCA plot, 2) added a heatmap of expression of 50 selected transcripts for gametocytes and schizonts (new Figure 5B) that further demonstrate the clustering and effects of PyNOT1-G upon specific transcripts, 3) have added additional descriptions in the labels of the MA plots and Volcano plots to help the reader to understand the data trends, and 4) have provided CirGO plots (new Figure 5GH) for both gametocytes and schizonts that further highlight the effects upon biological processes that are impacted by PyNOT1-G. The complete GO term lists remain in the supplement so that interested readers have all information available to them.*

*We have added the following text regarding the CirGO plots:*

*Lines 252-254: “CirGO plots of the gene ontology (GO) terms (biological processes) associated with the differentially expressed transcripts illustrate this further, with several specific terms present that are related to these same processes, structures, and features for both gametocytes and asexual blood stage schizonts (Figure 5GH).”*

*Lines 535-536: “Gene ontology terms for the differentially expressed transcripts were summarized using REVIGO and were plotted using CirGO, with GO terms that are single clusters labelled as null [56,57].”*

-	the authors over-interpret the localization of Not1-G and do not reach the limit of fluorescence microscopy, as stated in their response. At this resolution and in the absence of co-localisation, many proteins unrelated to the CAF1/CCR4/NOT complex will show a similar localization pattern. Importantly, the authors would have to demonstrate that the apparent nuclear localization of alpha-tubulin in male gametocytes is linked to non-confocal imaging. This is, at least, not the case in our hands.

*Response: We have* *replaced the image panels for male gametocytes for both PyNOT1::GFP and PyNOT1-G::GFP with higher quality images (Figure 2). Alpha tubulin is a common marker for* Plasmodium *male gametocytes (e.g., high abundance in male gametocytes) since its first description by Rawlings et al. Molecular and Biochemical Parasitology 1992, and we and others have routinely used it as a counterstain for male gametocytes (Hart et al. PLoS Pathogens 2019, Bowman and Finger et al. Malaria Journal 2020, Hitz et al. Scientific Reports 2020, etc.).*

*We have revised the manuscript by providing the following text regarding the expression and localization patterns for PyNOT1 and PyNOT1-G in Lines 139-141: “Both NOT1 paralogues were expressed and localized at the same times in the life cycle, and the expression pattern for PyNOT1 and PyNOT1-G is cytosolic and at least partially punctate. This is consistent with the localization of other members of the CAF1/CCR4/NOT complex in Plasmodium, model eukaryotes, and human cells [21, 22].*

COMMENTS FROM THE ACCADEMIC EDITOR:

The reviewer is not satisfied with the visual presentation of the rnaseq data. GO analysis is covered in the text and the full dataset is available in the supplementary tables but the reviewer requested a figure for the GO, something like CirGO would be very easy to make and would reduce complexity and clarify how specific to the described genes and genesets the dysregulation is, ie whether there were lots of other GO categories also dysregulated. I am a little surprised at the authors' response because the GO analysis seems to nicely support their hypotheses. I support the reviewer here as readers should be able to assess how valid the conclusions re GO enrichment were without consulting supplementary tables. I don’t think that including a simple CirGO or similar plot would overwhelm fig 5 which is currently carrying 2 essentially redundant panels that use orthologous approaches to demonstrate reproducibility of the replicates. These are the PCA plot and correlation heatmap of the replicates. The reviewer initially asked for heatmaps of expression of the genes discussed in the text, not the heatmap of the correlation between replicates provided by the authors. Although it is nice to include evidence of the reproducibility of the rnaseq. I would suggest moving the pca or the correlation heatmap to the supplement and adding the heatmap of gene expression that was requested by the reviewer as well as a visual representation of the disregulated GOs.

*Response: We have made all these suggested changes in this revision. We have retained the PCA plot, added a heatmap of expression of selected transcripts, and have added CirGO plots. Please see our response above to Reviewer 3 for further details.*

The RNASeq data in Fig 5 is also lacking a clear labelling of the bland altman/MA plots in fig 5 c and d, these are not labelled adequately nor explained in the legend, log fold change of what? Presumably its KD/wildtype but this needs to be explained in the legend or figure. Similarly the volcano plots in 5 e and d are not explained, again I presume its KO/wildtype but this needs to be explained in the legend or the figure.

*Response: We have made all these suggested changes in this revision. Figure 5 now has the additional labels provided to denote these fold changes are pynot1-g^-^ / WT. Please see our response to Reviewer 3 for further details.*

The RNAseq is described in lines 253 – 268 but the authors describe fold change but not p values or FDR, this is provided in the supplementary tables but they should explicitly state the threshold for significance for the fold changes they describe in the text.

*Response: We have added the p-values used in the previous studies with pbdozi^-^, pbcith^-^, pyalba4^-^ (p < 0.05). As FDRs were not reported for those studies, we have not included that information here. We have included the FDR thresholds used in this study at appropriate points throughout the manuscript.*

The reviewer is unconvinced by the fluorescence microscopy of not-1. I share their reservations about the cytoplasmic pattern being indicative of an association with caf1 and ccr4 but I agree with the authors that the staining is partially punctate. I suggest they moderate their interpretation to state that the staining pattern of not-1 is cytoplasmic and partially punctate consistent with other cytoplasmic proteins including the previously observed patterns of ccr4 and caf1. As the authors themselves note the co-IP data is what shows an interaction between not1 and the ccr4 complex components, the imaging is not very informative and doesn’t need to be over-interpreted.

It’s unclear to me whether the reviewer is concerned that the alpha tubulin staining is non-specific and therefore the parasite may not be a male gametocyte, or whether they are concerned that the quality of the fluorescence microscopy is inadequate because of the depth of field captured. The former then this is a genuine concern that should be addressed by staining more cells with anti alpha tubulin and showing that only male gametocytes are stained, preferably also staining with an additional male gam specific antibody. Failing that presumably the authors have some negative control asexual parasites they stained at the same that they could show. If it is the latter concern then I think it is a technical issue that doesn’t really affect the interpretation of the data, ie the not 1 stain is clearly outside the nucleus so the parasite has not1 outside the nucleus.

*Response: We have addressed both possibilities by providing higher quality images for the male gametocytes in question here, which more closely align with what has been published by our group and by others. We have also moderated our descriptions of these data (see below). Alpha tubulin is a common marker for Plasmodium male gametocytes (e.g., high abundance in male gametocytes) since its first description by Rawlings et al. Molecular and Biochemical Parasitology 1992, and we and others have routinely used it as a counterstain for male gametocytes (Hart et al. PLoS Pathogens 2019, Bowman and Finger et al. Malaria Journal 2020, Hitz et al. Scientific Reports 2020, etc.).*

*As also noted in response to Reviewer 3, we have included new text on Lines 139-141 that revises our description of the localization of PyNOT1 and PyNOT1-G. We now state the following:*

*“Both NOT1 paralogues were expressed and localized at the same times in the life cycle, and the expression pattern for PyNOT1 and PyNOT1-G is cytosolic and at least partially punctate. This is consistent with the localization of other members of the CAF1/CCR4/NOT complex in Plasmodium, model eukaryotes, and human cells [21, 22].”*
